# Generation of the Camptothecin Scaffold by a Flavin‐Catalyzed Photooxidative Skeletal Reorganization

**DOI:** 10.1002/anie.4569029

**Published:** 2026-07-01

**Authors:** Shenyu Liu, Zeliang Zhang, Benedikt Seligmann, Van Hung Bui, Tuan‐Anh Minh Nguyen, Thu‐Thuy T. Dang, Jakob Franke

**Affiliations:** ^1^ Centre of Biomolecular Drug Research Leibniz University Hannover Hannover Germany; ^2^ Institute of Botany Leibniz University Hannover Hannover Germany; ^3^ Department of Chemistry Irving K. Barber Faculty of Science University of British Columbia Kelowna British Columbia Canada

**Keywords:** biomimetic, biosynthesis, monoterpene indole alkaloid, photooxidation, pumiloside

## Abstract

The plant alkaloid camptothecin is a high‐value precursor for the semi‐synthesis of multiple anticancer drugs. The key transformation during its biosynthetic pathway is a yet enigmatic skeletal reorganization from a 6/5/6 tetrahydro‐β‐carboline ring system to the 6/6/5 pyrroloquinoline scaffold, which is characteristic of camptothecin‐related alkaloids. Here, we show that this scaffold transition can be efficiently catalyzed by flavin cofactors such as flavin mononucleotide in a photooxidative process. As part of this complex transformation, we verified the existence of a previously postulated macrocyclic ketolactam intermediate. Based on the mild conditions—oxygen, light, and flavins—we show that this conversion can also take place in leaves of *Nicotiana benthamiana*, a plant which does not normally produce camptothecin or related alkaloids. Our optimized biomimetic photochemical reaction conditions enable a short, mild, and efficient semi‐synthesis of the alkaloids (3*S*)‐pumiloside, (3*R*)‐pumiloside, and vincosamide ketolactam, also known as turpiniside, without any protecting groups. Thus, our work improves our understanding of this key skeletal reorganization step forming the camptothecin scaffold in nature and provides streamlined photocatalytic access to camptothecin‐related alkaloids.

## Graphical abstract

The biosynthetic pathway of the anticancer drug precursor camptothecin involves an enigmatic skeletal reorganization from a 6/5/6 tetrahydro‐β‐carboline to a 6/6/5 pyrroloquinoline ring system. Here, we show that this transformation can be achieved by photooxidation with flavin cofactors, such as flavin mononucleotide (FMN) as catalysts. This biomimetic photoreaction thereby provides short, efficient, and mild access to the camptothecin scaffold.

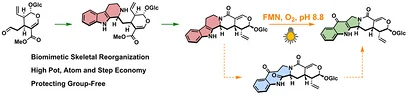

## 1 Introduction

**FIGURE 1:**
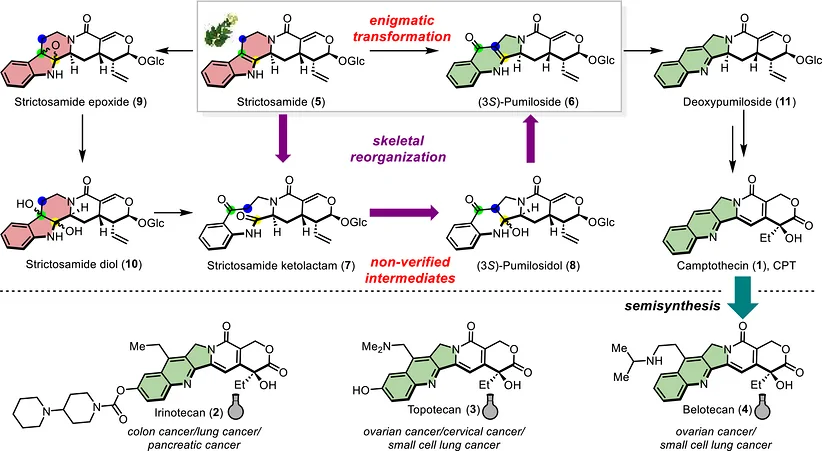
The proposed biosynthesis of the anticancer drug precursor camptothecin (**1**) involves an enigmatic skeletal reorganization step from a 6/5/6 tetrahydro‐β‐carboline ring system to the 6/6/5 pyrroloquinoline scaffold, which is characteristic for camptothecin (**1**).

The plant alkaloid camptothecin (**1**) is the precursor to several clinically used anticancer drugs, for example, irinotecan (**2**), topotecan (**3**), and belotecan (**4**). A unique structural feature of camptothecin (**1**) is that—although it belongs biosynthetically to the class of monoterpene indole alkaloids—it does not contain the classical 6/5/6 tetrahydro‐β‐carboline ring system of monoterpene indole alkaloids anymore, but rather a 6/6/5 pyrroloquinoline scaffold. Based on chemical work [1], isolation of potential intermediates [2, 3, 4, 5, 6], and isotope labelling experiments [7, 8], it has been proposed that the main pyrroloquinoline scaffold of camptothecin (**1**) is generated by an enigmatic skeletal reorganization step from the key intermediate strictosamide (**5**) to (3*S*)‐pumiloside (**6**) (Figure 1). So far, the mechanism for this pivotal conversion of strictosamide (**5**) to (3*S*)‐pumiloside (**6**) has remained hypothetical. It is postulated that this transformation occurs via a ketolactam intermediate (**7**), featuring a 9‐membered macrocycle [5, 7]. This ketolactam **7** could then undergo an intramolecular aldol reaction to (3*S*)‐pumilosidol (**8**) and after elimination of water form (3*S*)‐pumiloside (**6**) [7]. The B/C ring cleavage of strictosamide (**5**) could occur by monooxygenation to strictosamide epoxide (**9**). Hydrolysis of this epoxide **9** would then lead to strictosamide diol (**10**). A second oxidation would then result in diol cleavage to the putative ketolactam intermediate **7**. While two cytochrome P450 monooxygenases and an epoxide hydrolase have been reported to be involved in strictosamide (**5**) epoxidation and hydrolysis [9, 10, 11], these studies have not yet established a clear link between strictosamide (**5**) and pumiloside (**6**). A better understanding of the chemical logic of this key transformation of camptothecin (**1**) biosynthesis would therefore not only help to elucidate the enzymatic basis of this reaction, but also facilitate synthetic access to pumiloside (**6**), deoxypumiloside (**11**), camptothecin (**1**), and related anticancer drugs.

It was reported before that strictosamide (**5**) is prone to photochemical conversion to (3*S*)‐pumiloside (**6**) in the presence of oxygen by an unknown mechanism; however, the reported reaction was very sluggish, requiring up to seven days at room temperature or refluxing [12, 13]. On the other hand, it was shown that stoichiometric amounts of flavins lead to ring expansion of fungal indole diterpenes [14]. Stimulated by these findings, we wanted to explore whether flavin catalysis might facilitate the conversion of strictosamide (**5**) to (3*S*)‐pumiloside (**6**). We here now report that, indeed, flavins act as efficient catalysts for a photooxidative skeletal reorganization that is dependent on light and oxygen. We further verify the existence of a previously postulated ketolactam intermediate and demonstrate that this reaction can also take place in non‐camptothecin producing plants including *Nicotiana benthamiana*. The discovery of optimized photochemical reaction conditions enables a short, mild, and efficient semi‐synthesis of (3*S*)‐pumiloside and its C‐3 epimer. Our findings therefore shed light on the scaffold‐defining key transformation of the anticancer drug precursor camptothecin (**1**) and will facilitate access to camptothecin‐related alkaloids by efficient synthetic processes in the future.

## 2 Results and Discussion

### 2.1 Conversion of Strictosamide (**5**) to (3*S*)‐Pumiloside (**6**) by Flavins

**FIGURE 2:**
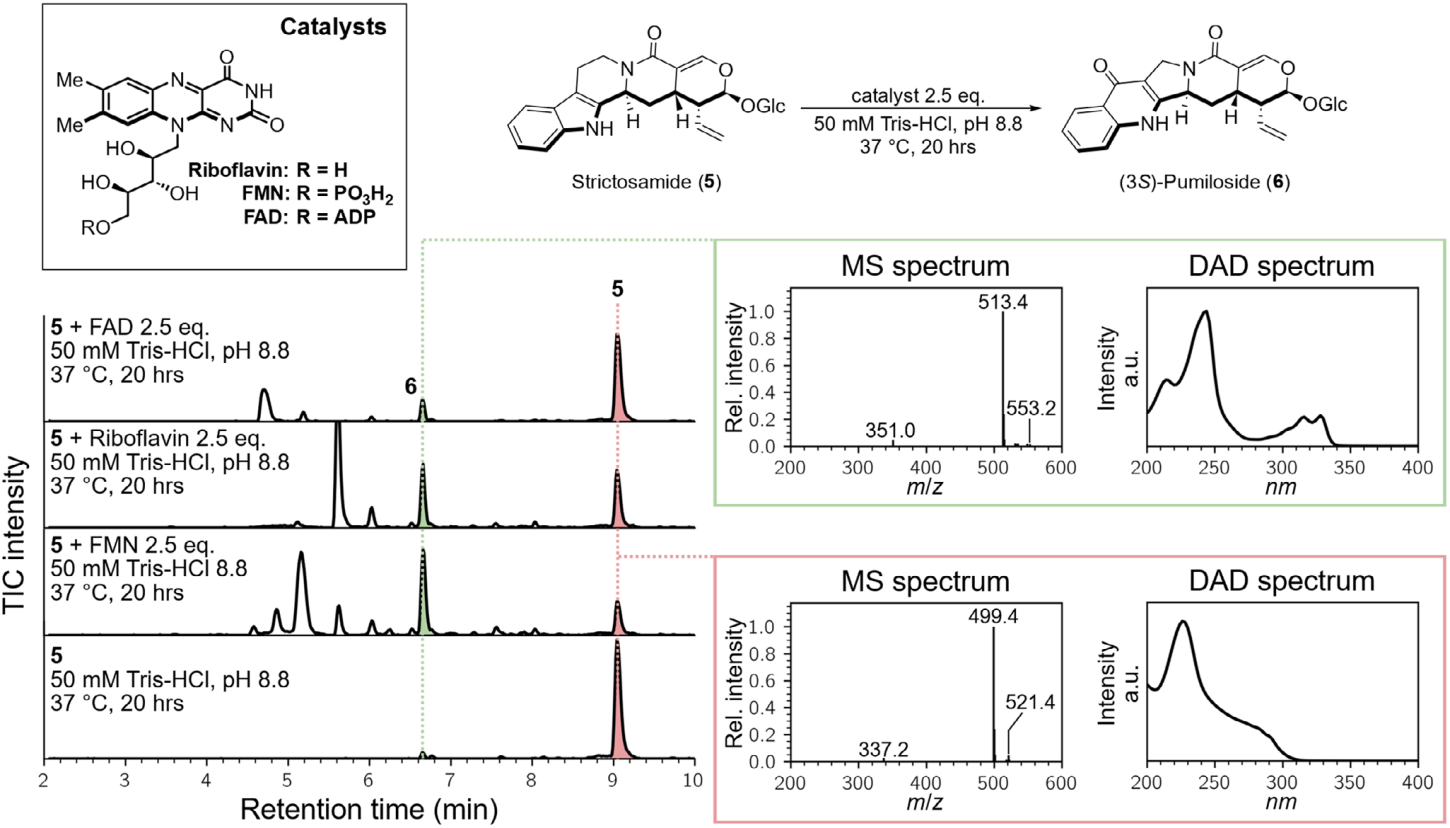
Flavin‐dependent conversion of strictosamide (**5**) to (3*S*)‐pumiloside (**6**). The UV spectrum of (3*S*)‐pumiloside (**6**) agreed with literature data [2]. The identity of (3*S*)‐pumiloside (**6**) was additionally confirmed by NMR spectroscopy. The major additional peaks are related to the flavin catalysts (Figure S1). DAD, diode array detector; FAD, flavin adenine dinucleotide; FMN, flavin mononucleotide; MS, mass spectrometry; TIC, total ion chromatogram.

As stoichiometric amounts of flavins have been reported to lead to ring expansion of fungal indole diterpenes [14], we wanted to explore if flavin cofactors, such as flavin mononucleotide (FMN), flavin adenine dinucleotide (FAD), or riboflavin could mediate the conversion of strictosamide (**5**) to (3*S*)‐pumiloside (**6**) in a similar manner. Following the experimental conditions from this previous work [14], we treated purified strictosamide (**5**) with 2.5 eq. of flavin cofactors (Figure 2). Indeed, LCMS chromatograms showed a clear peak that occurred consistently in all samples containing strictosamide (**5**) and a flavin; in the absence of flavins, only traces of this compound were detectable. Compared to strictosamide (**5**), the UV spectrum of this new peak showed a substantial change including a bathochromic shift from 275 to 325 nm, suggesting the presence of an expanded chromophore. This UV spectrum was in excellent agreement with reported data for (3*S*)‐pumiloside (**6**) [2]; also, the mass spectrum showed a molecular ion with *m*/*z* 513 which would fit to (3*S*)‐pumiloside (**6**). Isolation and NMR‐based structure elucidation of this compound confirmed that it was indeed (3*S*)‐pumiloside (**6**) (Table S1). Taken together, this experiment revealed that flavins also play a role for the skeletal reorganization of strictosamide (**5**) to (3*S*)‐pumiloside (**6**), yet mechanistic clarification is needed to optimize the reaction.

### 2.2 Flavin as a Catalyst for a Photooxidation

**FIGURE 3:**
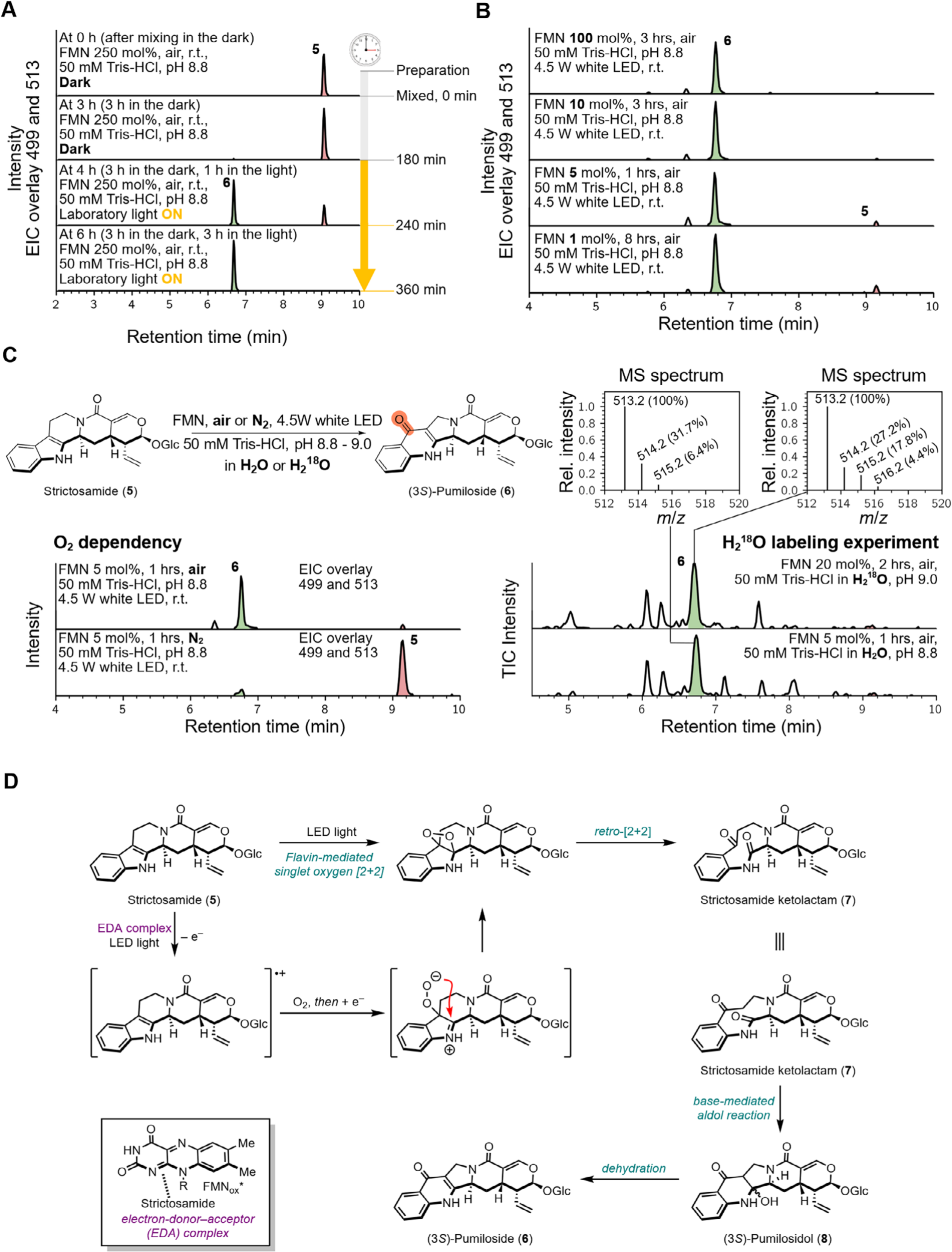
The conversion of strictosamide (**5**) to (3*S*)‐pumiloside (**6**) depends on light, is catalytic and oxygen‐dependent. (A) Light dependence of the flavin‐dependent conversion of strictosamide (**5**). (B) Course of the photoreaction with catalytic amounts of FMN. (C) Mechanistic experiments to test the dependence on O_2_ and the incorporation of oxygen from H_2_^18^O. (D) Proposed mechanistic routes for the photooxidative conversion of strictosamide (**5**) to (3*S*)‐pumiloside (**6**). EDA, electron‐donor−acceptor.

It was previously reported that strictosamide (**5**) can be slowly converted to (3*S*)‐pumiloside (**6**) mediated by light [12, 13]. We, therefore wanted to test if our observed (3*S*)‐pumiloside (**6**) formation might have been influenced by background light that was not controlled in our initial experiments. To verify this, we treated strictosamide (**5**) with an excess of FMN (2.5 eq.) in a light‐dependent manner (Figure 3A). In the dark, no reaction was observed over 3 h. In contrast, light from the fume hood was sufficient to lead to rapid and complete conversion of strictosamide (**5**) to (3*S*)‐pumiloside (**6**) within 3 h. These results indicate that not only flavin but also light is crucial for this conversion. In comparison to the previously reported sluggish light‐dependent conversion of strictosamide (**5**) to (3*S*)‐pumiloside (**6**), which required multiple days [12, 13], our results show that the presence of flavin can drastically accelerate this reaction.

We suspected that the mechanistic basis for the requirement of both light and flavin might be that flavin acts as a catalyst for a photooxidation of strictosamide (**5**). To support this idea, we first wanted to verify that this reaction is truly catalytic and does not require stoichiometric amounts of flavin as used for the ring expansion of fungal indole diterpenes [14]. For higher light efficiency, we replaced the fume hood light with a 4.5 W white LED lamp. Indeed, 5 mol% FMN with a reaction time of 1 h was sufficient for almost complete conversion of strictosamide (**5**) to (3*S*)‐pumiloside (**6**), representing a major improvement over the previously reported non‐catalytic reaction conditions requiring multiple days [12, 13]. For fungal indole diterpenes, it was reported that the oxygen atoms introduced by this flavin‐dependent reaction are derived from water rather than molecular oxygen [14]. We therefore tested if the reaction is dependent on molecular oxygen; in a nitrogen atmosphere, only very small amounts of (3*S*)‐pumiloside (**6**) were observed (Figure 3C). On the other hand, when performing the reaction in H_2_^18^O instead of H_2_^16^O, the M+2 peak at *m*/*z* 515 was only weakly increased from 6.4% to 17.8%. This weak but nonetheless clearly noticeable incorporation of oxygen from water might be explained by reversible hydration of the keto intermediate (Figure S2) or alternatively by the co‐existence of a second minor mechanistic pathway for this transformation (Figure S3), similar to what has been proposed for fungal indole diterpenes [14]. Nonetheless, based on our results and on photochemical studies, we propose that flavin either acts as a photosensitizer to mediate a [2+2] cycloaddition with singlet oxygen [15, 16] or alternatively forms an electron‐donor−acceptor (EDA) complex with the substrate strictosamide (**5**) to trigger a photooxidation via single‐electron transfer (Figure 3D) [17, 18, 19]. Both routes would lead to an 1,2‐dioxetane intermediate, which could undergo a retro‐[2+2] reaction to strictosamide ketolactam (**7**), the same intermediate that has also been proposed for the monooxygenation‐based pathway [5, 7]. To test if singlet oxygen is involved, we carried out the reaction in D_2_O instead of H_2_O, as D_2_O is known to increase the lifetime of singlet oxygen [20]. Indeed, faster substrate consumption was observed in D_2_O compared to H_2_O (Figure S4). Furthermore, we investigated if we could detect formation of an EDA complex by UV–vis spectroscopy (Figure S5); however, no new absorption band that might indicate the presence of an EDA complex was observed in the 500–600 nm region compared to the sum of the individual components. Lastly, we tested if other photosensitizers, such as rose Bengal, methylene blue, chlorophyll, or tetraphenylporphyrin would be able to catalyze this reaction; indeed, with all four tested catalysts, the substrate strictosamide (**5**) was completely or almost completely converted to (3*S*)‐pumiloside (**6**) (Figure S6). In the absence of a catalyst, only very minor levels of the substrate were converted to (3*S*)‐pumiloside (**6**) upon irradiation.

Taken together, our data show that FMN—as well as other photosensitizers—acts as a catalyst for the photooxidative skeletal reorganization of strictosamide (**5**) to (3*S*)‐pumiloside (**6**).

### 2.3 Verification of a Dedicated Ketolactam Intermediate

**FIGURE 4:**
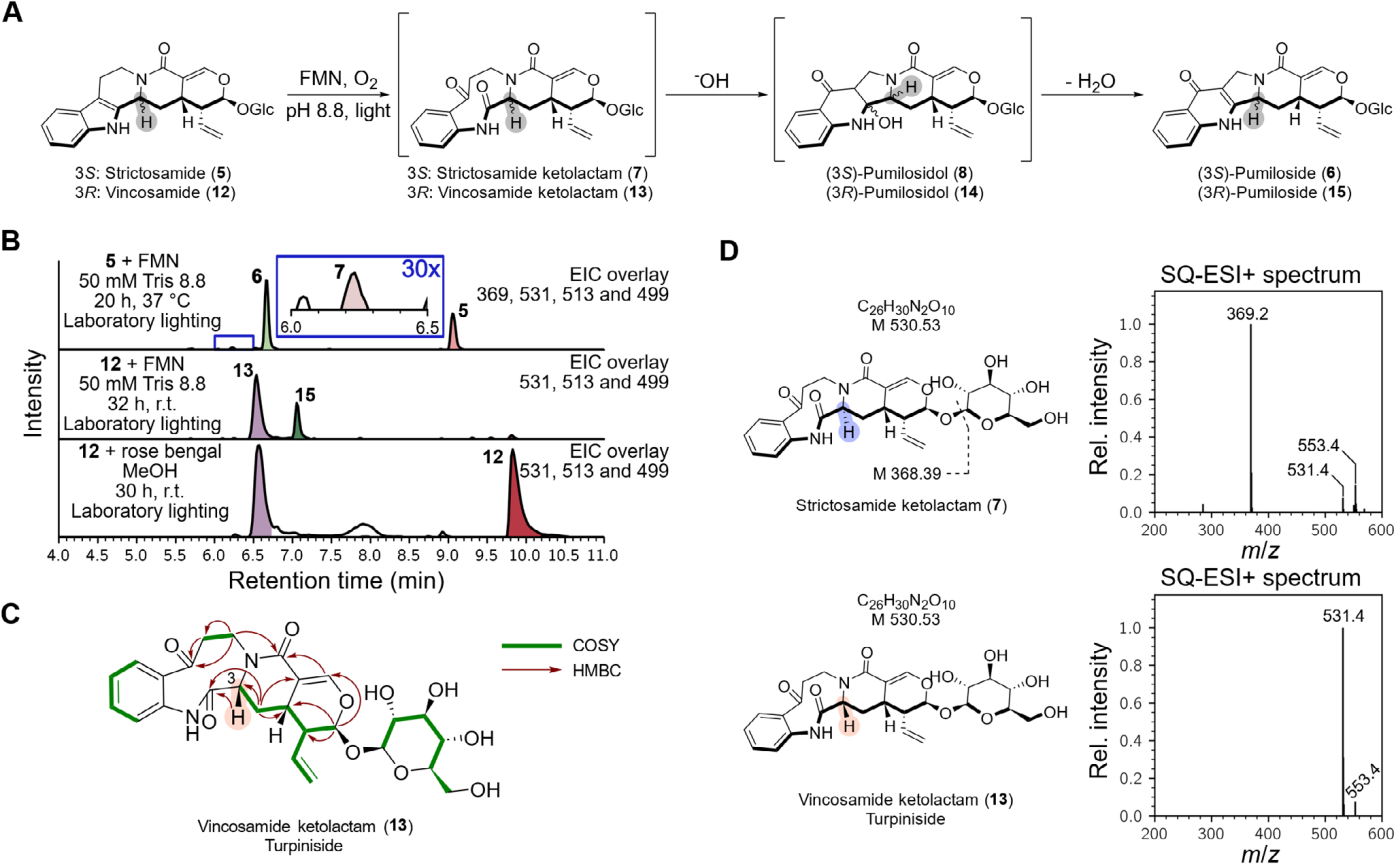
Verification of dedicated ketolactam intermediates. (A) Scheme showing putative ketolactam intermediates (**7** and **13**) and ketoaminal intermediates (**8** and **14**) en route to (3*S*)‐pumiloside (**6**) and (3*R*)‐pumiloside (**15**). (B) Occurrence of a peak with *m*/*z* 531 potentially corresponding to ketolactam or ketoaminal intermediates during the photooxidation of strictosamide (**5**). The inset shows the 30x zoomed‐in region highlighted by the blue box. (C) Key NMR correlations of isolated intermediate vincosamide ketolactam (**13**), also known as turpiniside [21]. All attempts to also isolate intermediate strictosamide ketolactam (**7**) resulted in spontaneous conversion to (3*S*)‐pumiloside (**6**). (D) Comparison of MS spectra (electrospray ionization) of strictosamide ketolactam (**7**) and vincosamide ketolactam (**13**). A detailed comparison of MS/MS spectra is shown in Figure S10 and Tables S5, S6. SQ‐ESI+, single quadrupole‐electrospray ionization (positive mode).

Previous proposals suggested the formation of the dedicated ketolactam intermediate **7** en route to (3*S*)‐pumiloside (**6**), but such a discrete intermediate from this transformation has never been isolated, potentially due to its high reactivity. We therefore sought to provide structural support for this intermediate, which would help to further understand the mechanistic course. In our chromatograms, we could detect a peak with *m*/*z* 531, which would match either strictosamide ketolactam (**7**) or its cyclic ketoaminal isomer (3*S*)‐pumilosidol (**8**) (Figure 4A). Efforts to isolate this compound, however, were fruitless, as spontaneous conversion to (3*S*)‐pumiloside (**6**) occurred during all purification attempts. Hoping to stabilize this intermediate, we also tested the effect of pH on the conversion of strictosamide (**5**) to (3*S*)‐pumiloside (**6**) (Figure S7). While we noted a clear pH dependence of the conversion efficiency with a maximum at pH 8.8, no discrete intermediate accumulated at sub‐optimal pH values.

As halting the conversion of strictosamide (**5**) to (3*S*)‐pumiloside (**6**) at the intermediate stage was not successful, we considered alternative strategies. We speculated that the stereochemistry at C‐3 might influence the preferred conformation of the 9‐membered macrocyclic ring of a ketolactam intermediate and thereby affect the efficiency of the intramolecular aldol condensation. In theory, vincosamide (**12**), the *3R* stereoisomer of strictosamide (**5**), should be able to react via vincosamide ketolactam (**13**) and (3*R*)‐pumilosidol (**14**) to (3*R*)‐pumiloside (**15**) in a process analogous to the skeletal reorganization of strictosamide (**5**). Indeed, when we treated vincosamide (**12**) under the same photochemical conditions as before, we observed conversion to (3*R*)‐pumiloside (**15**), demonstrating that the reaction conditions were in principle independent of the C‐3 stereochemistry (Figures 4B, S8). The identity of (3*R*)‐pumiloside (**15**) was confirmed by NMR spectroscopy (Table S2). However, the peak corresponding to one of the putative reaction intermediates **13** or **14** was substantially larger than for the epimeric substrate. We envisioned that avoiding base might also help to hamper the aldol reaction at the ketolactam stage. To our delight, replacing FMN with the known photocatalyst rose Bengal [22] in the absence of base resulted in complete halt of the downstream intramolecular aldol condensation (Figure 4B), yielding intermediate **13** as the single major product. Isolation and structure elucidation of **13** verified that this was indeed vincosamide ketolactam (Figures 4C, S9). We noted that vincosamide ketolactam (**13**) is actually a known natural product, termed turpiniside [21]. The producing plant *Turpinia arguta* (family Staphyleaceae) is not a known producer of camptothecin (**1**) or any related pyrroloquinoline alkaloid. NMR data of our isolated compound **13** were in excellent agreement with published data for turpiniside [21] (Tables S3, S4). Lastly, as we could not isolate pure strictosamide ketolactam (**7**) due to its high inherent propensity to undergo an intramolecular aldol reaction, we compared MS spectra (Figure 4D) and MS/MS spectra (Figure S10, Table S5, S6) of this compound with NMR‐verified vincosamide ketolactam (**13**). While both compounds showed the same fragments, putative strictosamide ketolactam (**7**) exhibited a much stronger loss of the glycosyl residue by in‐source fragmentation in the MS spectrum and at low collision energies (Figure S10A). At higher collision energies, both compounds exhibited almost identical MS/MS spectra (Figure S10D–F). Thus, our data confirms the existence of vincosamide ketolactam (**13**) as a dedicated intermediate for this skeletal reorganization reaction and supports the analogous formation of the epimeric intermediate strictosamide ketolactam (**7**).

To further support that basic conditions indeed trigger the intramolecular aldol reaction of vincosamide ketolactam (**13**) to (3*R*)‐pumiloside (**15**), we incubated isolated **13** under different conditions. At pH 8.8, vincosamide ketolactam (**13**) was partially converted to (3*R*)‐pumiloside (**15**) within 2 h, whereas the reaction proceeded to completion within this time at pH 10–11 (Figure S11A). Under acidic conditions, no conversion was observed (Figure S11A). Building on this requirement for basic conditions, we adjusted our protocol for the photooxidation of strictosamide (**5**); indeed, final basification to pH 10–11 helped to convert any remaining intermediates to pumiloside (**6**) (Figure S11B).

### 2.4 Formation of (3*S*)‐Pumiloside *in Planta*

**FIGURE 5:**
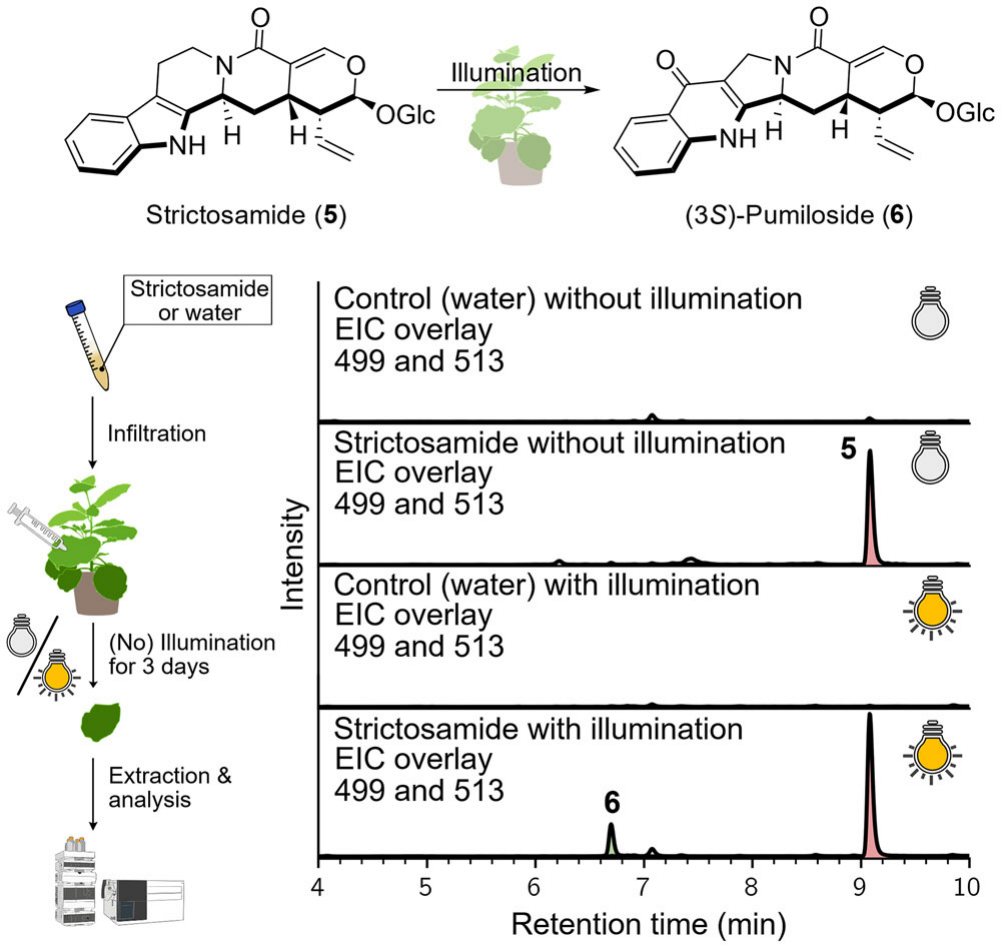
Light‐dependent formation of (3*S*)‐pumiloside (**6**) in leaves of the model plant *N. benthamiana*, which does not produce monoterpene indole alkaloids. Only strictosamide (**5**), but no additional FMN was infiltrated.

The conditions for the photochemical conversion of strictosamide (**5**) to (3*S*)‐pumiloside (**6**)—flavins, light, and oxygen—are also expected to occur within plant cells. We therefore wondered if this reaction could also take place *in planta*, particularly in a plant that is very unlikely to host enzymes that could catalyze this conversion specifically. For this purpose, we selected the common model plant *N. benthamiana* (family Solanaceae) [23], which does not produce any monoterpene indole alkaloid. We infiltrated strictosamide (**5**) into leaves of *N. benthamiana* and either kept the plants in the dark or illuminated them under standard phytochamber conditions for three days using a 16/8 h day/night cycle (Figure 5). In the dark, only traces of (3*S*)‐pumiloside (**6**) were observed. In contrast, for *N. benthamiana* plants that were exposed to light, a clear peak corresponding to (3*S*)‐pumiloside (**6**) was observed. This conversion of strictosamide (**5**) to (3*S*)‐pumiloside (**6**) also occurred in leaf disks of three additional non‐camptothecin‐producing plants (Figure S12) and in *Catharanthus roseus* petals (Figure S13), which are not considered a major native site of alkaloid and iridoid biosynthesis [24]. In vitro assays with crude enzyme fractions obtained from *N. benthamiana* leaves demonstrated that this conversion can occur non‐enzymatically in a light‐dependent manner (Figure S14). Taken together, the reaction conditions described here for this non‐enzymatic photoreaction of strictosamide (**5**) are also relevant under physiological conditions.

### 2.5 A Short, Mild, and Efficient Semisynthetic Route

**FIGURE 6:**
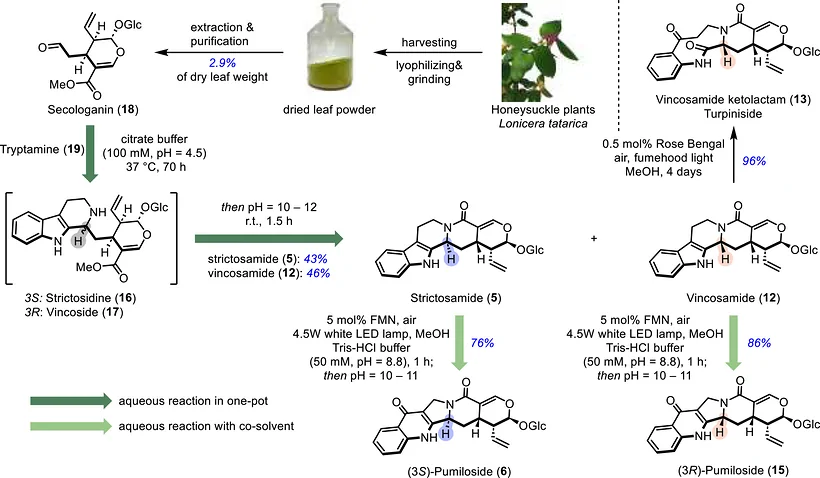
Short, mild, and efficient semi‐synthesis of (3*S*)‐pumiloside (**6**), (3*R*)‐pumiloside (**15**), and vincosamide ketolactam (turpiniside) (**13**) without protecting groups.

Our discovery of the flavin‐catalyzed photooxidative conversion of strictosamide (**5**) to (3*S*)‐pumiloside (**6**) and of vincosamide (**12**) to turpiniside (**13**) and (3*R*)‐pumiloside (**15**) could enable a short and very mild semisynthetic route to these alkaloids (Figure 6). However, strictosamide (**5**) is not readily available as a starting material in larger quantities without substantial costs. To overcome this bottleneck, we envisioned that strictosamide (**5**) and vincosamide (**12**) might be produced readily from strictosidine (**16**) and vincoside (**17**), which themselves could be produced non‐enzymatically from secologanin (**18**) and tryptamine (**19**) [25]. Secologanin (**18**) is the major iridoid found in leaves of Tatarian honeysuckle (*Lonicera tatarica*) [26, 27] and can easily be isolated from the plant material [28]. Treating secologanin (**18**) with tryptamine (**19**) resulted in a ratio of ca. 1:1 of the two C‐3 epimers strictosidine (**16**) and vincoside (**17**), which are difficult to separate. To our delight, direct conversion of this epimeric mixture by simple pH adjustment in the same vessel generated the corresponding amides strictosamide (**5**) and vincosamide (**12**), which were much easier to separate by column chromatography. Thereby, strictosamide (**5**) and vincosamide (**12**) were obtained in 43% and 46% yield, respectively, by this one‐pot reaction over two steps. Then, photooxidative conversion of strictosamide (**5**) and vincosamide (**12**) gave rise to (3*S*)‐pumiloside (**6**), (3*R*)‐pumiloside (**15**), and vincosamide ketolactam (turpiniside) (**13**) in 76%, 86%, and 96% yield. All reactions are carried out at room temperature or 37 °C, and most reactions employ aqueous conditions. As such, our work now enables a short, mild, and efficient semisynthetic route without any protecting groups to these important key intermediates of camptothecin (**1**) biosynthesis.

## 3 Discussion

In this work, we report that flavins catalyze the photooxidative skeletal reorganization of strictosamide (**5**) to (3*S*)‐pumiloside (**6**) and of vincosamide (**12**) to (3*R*)‐pumiloside (**15**) as a key scaffold‐forming step for camptothecin biosynthesis. In contrast to previous proposals that suggested a monooxygenation mechanism via strictosamide epoxide (**9**) [7, 8], our findings support an alternative route that is based on dioxygenation and would thereby skip the previously proposed intermediates strictosamide epoxide (**9**) and strictosamide diol (**10**) completely. In the past, several indole ring opening reactions and other oxidative transformations have been reported in organic synthesis [29, 30, 31, 32, 33, 34, 35]. However, these employed either other oxidants or photocatalysts other than flavins, such as chlorophyll [29], copper [36] or ruthenium [32, 37]. While it is known that flavins can act as photocatalysts [15, 16], this feature is only rarely utilized in biosynthetic and biomimetic pathways [38, 39]. During revision of this work, the photocatalytic reactivity of strictosamide (**5**) with flavin was also independently reported by others [40, 41]. It remains to be seen if this reactivity of strictosamide (**5**) and vincosamide (**12**) is also captured for enzymatic catalysis by a flavin‐dependent dioxygenase in nature.

Our data show that—in principle—this photoreaction can also take place in a plant such as *N*. *benthamiana* that is unlikely to possess a specific enzyme for this conversion. Thus, for camptothecin biosynthesis, it is possible that this non‐enzymatic reaction might contribute at least partially to this key step. However, the primary site of camptothecin biosynthesis in *Camptotheca acuminata* is the stem [42], where light might not penetrate to deeper tissue layers. Likewise, in *Ophiorrhiza pumila*, (3*S*)‐pumiloside (**6**) and related downstream camptothecin intermediates were also found in stem and roots [8]. Further experimentation will be needed to clarify if a dedicated enzyme can convert strictosamide (**5**) into (3*S*)‐pumiloside (**6**) or vincosamide (**12**) into (3*R*)‐pumiloside (**15**). Indeed, there are a few other examples of biosynthetic transformations that can occur spontaneously, but were later found to be supported by enzymes nonetheless [43, 44].

Due to their easy accessibility and chemical versatility, flavins are excellent tools for green and biomimetic synthesis including photocatalysis [45, 46, 47, 48, 49]. Our work underlines this potential by describing a short, mild and efficient semisynthetic route to (3*S*)‐pumiloside (**6**), (3*R*)‐pumiloside (**15**), and vincosamide ketolactam, also known as turpiniside (**13**). The alkaloid turpiniside (**13**), isolated from *T. arguta* [21], has so far not been accessible by any synthetic approach. For (3*S*)‐pumiloside (**6**), a non‐catalytic photochemical conversion from strictosamide (**5**) has already been reported [12, 13]. Our synthetic route improves this procedure not only by starting from the much more readily accessible precursor secologanin (**18**) [26, 27], but more importantly by drastically reducing the reaction times from multiple days to 1 h thanks to the catalytic activity of FMN. Despite the complex chemical structures of the substrates, the photooxidations described here proceed with excellent chemoselectivity even without protecting groups. The complete semisynthetic route exhibits high pot, atom and step economy. Hence, our work opens new and efficient avenues toward photooxidative generation of the camptothecin scaffold (**1**).

## 4 Conclusion

In summary, our discovery of a biomimetic flavin‐catalyzed photooxidative skeletal reorganization process sheds new light on the key scaffold‐defining transformation of camptothecin (**1**) biosynthesis. As our work implicates a dioxygenation‐based route to (3*S*)‐pumiloside (**6**) or (3*R*)‐pumiloside (**15**) under conditions prevalent in plant cells, our results reshape the current picture of the camptothecin pathway. Therefore, our work may help to fully elucidate the biosynthetic pathway of camptothecin (**1**) in the future. Furthermore, the mild semisynthetic reaction conditions reported here facilitate photocatalytic access to the pyrroloquinoline ring system of camptothecin (**1**) and could thus lead to the development of greener synthetic routes toward anticancer drugs in the future.

## Supporting Information

## Author Contributions

**Shenyu Liu**: writing – review and editing, investigation, conceptualization, methodology, and visualization. **Zeliang Zhang**: writing – review and editing, investigation, methodology, validation, and visualization. **Benedikt Seligmann**: visualization. **Van Hung Bui**: investigation. **Tuan‐Anh Minh Nguyen**: investigation. **Thu‐Thuy T. Dang**: writing – review and editing, funding acquisition, supervision, and project administration. **Jakob Franke**: writing – original draft, writing – review and editing, supervision, project administration, conceptualization, and funding acquisition.

## Conflicts of Interest

The authors declare no conflicts of interest.

## Supporting information




**Supporting File**: Supporting figures and tables, experimental procedures and compound characterization data is provided. The authors have cited additional references within the Supporting Information [42, 50, 51, 52].

## Data Availability

The data that supports the findings of this study are available in the supporting information of this article.

## References

[1] C. R. Hutchinson , A. H. Heckendorf , J. L. Straughn , P. E. Daddona , and D. E. Cane , “Biosynthesis of Camptothecin. 3. Definition of Strictosamide as the Penultimate Biosynthetic Precursor Assisted by Carbon‐13 and Deuterium NMR Spectroscopy,” Journal of the American Chemical Society 101 (1979): 3358–3369, 10.1021/ja00506a037.

[2] Z. Zhang , H. N. ElSohly , M. R. Jacob , D. S. Pasco , L. A. Walker , and A. M. Clark , “New Indole Alkaloids From the Bark of Nauclea Orientalis,” Journal of Natural Products 64 (2001): 1001–1005, 10.1021/np010042g. 11520214

[3] X. Pu , C.‐R. Zhang , H.‐C. Gao , et al., “Biosynthesis‐inspired Mining and Identification of Untapped Alkaloids in Camptotheca Acuminate for Enzyme Discovery Using Ultra‐high Performance Liquid Chromatography Coupled With Quadrupole‐Time of Flight‐Mass Spectrometry,” Journal of Chromatography A 1620 (2020): 461036, 10.1016/j.chroma.2020.461036. 32201039

[4] N. Aimi , M. Nishimura , A. Miwa , H. Hoshino , S. Sakai , and J. Haginiwa , “Pumiloside and Deoxypumiloside; Plausible Intermediates of Camptothecin Biosynthesis,” Tetrahedron Letters 30 (1989): 4991–4994, 10.1016/S0040-4039(01)80563-3.

[5] B. K. Carte , C. DeBrosse , D. Eggleston , et al., “Isolation and Characterization of a Presumed Biosynthetic Precursor of Camptothecin From Extracts of *Camptotheca Acuminata* ,” Tetrahedron 46 (1990): 2747–2760, 10.1016/S0040-4020(01)88369-1.

[6] M. Kitajima , S. Masumoto , H. Takayama , and N. Aimi , “Isolation and Partial Synthesis of 3(*R*)‐ and 3(*S*)‐deoxypumiloside; Structural Revision of the Key Metabolite From the Camptothecin Producing Plant, *Ophiorrhiza Pumila* ,” Tetrahedron LETTERS 38 (1997): 4255–4258, 10.1016/S0040-4039(97)00858-7.

[7] R. Sadre , M. Magallanes‐Lundback , S. Pradhan , et al., “Metabolite Diversity in Alkaloid Biosynthesis: A Multilane (Diastereomer) Highway for Camptothecin Synthesis in Camptotheca Acuminata,” Plant Cell 28 (2016): 1926–1944, 10.1105/tpc.16.00193. 27432874 PMC5006703

[8] M. Yang , Q. Wang , Y. Liu , et al., “Divergent Camptothecin Biosynthetic Pathway in Ophiorrhiza Pumila,” BMC Biology 19 (2021): 122, 10.1186/s12915-021-01051-y. 34134716 PMC8207662

[9] T. Zhang , Y. Wang , S. Wu , et al., “Chemoproteomics Reveals the Epoxidase Enzyme for the Biosynthesis of Camptothecin in Ophiorrhiza Pumila,” Journal of Integrative Plant Biology 66 (2024): 1044–1047, 10.1111/jipb.13594. 38095243

[10] X. Pu , M. Wang , M. Chen , et al., “Proteomics‐Guided Mining and Characterization of Epoxidase Involved in Camptothecin Biosynthesis From *Camptotheca Acuminata* ,” ACS Chemical Biology (2023): 1772–1785, 10.1021/acschembio.3c00222, acschembio.3c00222. 37523250

[11] X. Pu , X.‐Y. Lin , J.‐W. He , et al., “Multiomics‐Guided Mining and Characterization of Epoxide Hydrolase Involved in Camptothecin Biosynthesis From *Camptotheca acuminata* ,” Bioorganic Chemistry 153 (2024): 107980, 10.1016/j.bioorg.2024.107980. 39577154

[12] L. I. U. Wenjun , L. I. Zhaoliang , M. Zhaoqing , et al., “Photochemical Degradation of the Strictosamide†,” Chemical Journal of Chinese Universities 35 (2014): 1710.

[13] X. Wei , W. Zhenzhong , D. Gang , et al., (Jiangsu Kanion Pharmaceutical Co., Ltd.), *Pumiloside Preparation Method*, CN105273013A (2016), https://worldwide.espacenet.com/patent/search/family/055142906/publication/CN105273013A?q=pn%3DCN105273013A.

[14] Y. Dai , X.‐L. Xie , H.‐F. Dai , and S.‐M. Li , “Fungal 2,18‐Dioxo‐2,18‐seco Indole Diterpenes by Nonenzymatic Flavin‐Catalyzed Oxidative Ring Expansion and Oxygen Incorporation From Solvent Water,” Organic Letters 25 (2023): 4092–4097, 10.1021/acs.orglett.3c01320. 37249271

[15] B. König , S. Kümmel , E. Svobodová , and R. Cibulka , “Flavin Photocatalysis,” Physical Sciences Reviews 3 (2018): 20170168, 10.1515/psr-2017-0168.

[16] P. F. Heelis , “The Photophysical and Photochemical Properties of Flavins (isoalloxazines),” Chemical Society Reviews 11 (1982): 15–39, 10.1039/cs9821100015.

[17] J. Zhu , Q. Zhang , T. Gu , et al., “Atom‐Economic Enantioselective Photoenzymatic Radical Hydroalkylation via Single‐Electron Oxidation of Carbanions,” Nature Catalysis 8 (2025): 1188–1197, 10.1038/s41929-025-01434-2.

[18] A. K. Wortman and C. R. J. Stephenson , “EDA Photochemistry: Mechanistic Investigations and Future Opportunities,” Chemistry 9 (2023): 2390–2415, 10.1016/j.chempr.2023.06.013. PMC10588808 37873033

[19] G. E. M. Crisenza , D. Mazzarella , and P. Melchiorre , “Synthetic Methods Driven by the Photoactivity of Electron Donor–Acceptor Complexes,” Journal of the American Chemical Society 142 (2020): 5461–5476, 10.1021/jacs.0c01416. 32134647 PMC7099579

[20] P. R. Ogilby , “Singlet Oxygen: There Is Indeed Something New Under the Sun,” Chemical Society Reviews 39 (2010): 3181–3209, 10.1039/b926014p. 20571680

[21] M. Wu , P. Wu , H. Xie , G. Wu , and X. Wei , “Monoterpenoid Indole Alkaloids Mediating DNA Strand Scission From Turpinia arguta,” Planta Medica 77 (2011): 284–286, 10.1055/s-0030-1250239. 20717879

[22] S. Sharma and A. Sharma , “Recent Advances in Photocatalytic Manipulations of Rose Bengal in Organic Synthesis,” Organic & Biomolecular Chemistry 17 (2019): 4384–4405, 10.1039/C9OB00092E. 30951059

[23] J. Bally , H. Jung , C. Mortimer , et al., “The Rise and Rise of *Nicotiana Benthamiana*: A Plant for all Reasons,” Annual review of phytopathology 56 (2018): 405–426, 10.1146/annurev-phyto-080417-050141. 30149789

[24] A. H. Vu , M. Kang , J. Wurlitzer , et al., “Quantitative Single‐Cell Mass Spectrometry Provides a Highly Resolved Analysis of Natural Product Biosynthesis Partitioning in Plants,” Journal of the American Chemical Society 146 (2024): 23891–23900, 10.1021/jacs.4c06336. 39138868 PMC11363012

[25] J. Franke , J. Kim , J. P. Hamilton , et al., “Gene Discovery in *Gelsemium* Highlights Conserved Gene Clusters in Monoterpene Indole Alkaloid Biosynthesis,” Chembiochem 20 (2019): 83–87, 10.1002/cbic.201800592. 30300974

[26] H. Zhu , Y. Cai , S. Ma , et al., “Privileged Biorenewable Secologanin‐Based Diversity‐Oriented Synthesis for Pseudo‐Natural Alkaloids: Uncovering Novel Neuroprotective and Antimalarial Frameworks,” Chemsuschem 14 (2021): 5320–5327, 10.1002/cssc.202101868. 34636473

[27] A. Hermans‐Lokkerbol and R. Verpoorte , “Purification of Secologanin From Lonicera Tatarica Extracts Using RLCC,” Planta Medica 53 (1987): 546–548, 10.1055/s-2006-962808. 17269097

[28] B. Seligmann , S. Liu , M. Huynh , T.‐T. T. Dang , and J. Franke , “Production of the Anticancer Drug Intermediate Strictosidinic Acid in Engineered Yeast,” Journal of Biotechnology 415 (2026): 1–12, 10.1016/j.jbiotec.2026.03.017. 41856293

[29] S. Banu , S. Choudhari , G. Patel , and P. P. Yadav , “Auto‐tandem PET and EnT Photocatalysis by Crude Chlorophyll Under Visible Light Towards the Oxidative Functionalization of Indoles,” Green Chemistry 23 (2021): 3039–3047, 10.1039/D1GC00138H.

[30] W. Adam , M. Ahrweiler , K. Peters , and B. Schmiedeskamp , “Oxidation of N‐Acylindoles by Dimethyldioxirane and Singlet Oxygen: Substituent Effects on Thermally Persistent Indole Epoxides and Dioxetanes,” Journal of Organic Chemistry 59 (1994): 2733–2739, 10.1021/jo00089a016.

[31] W. Adam , M. Ahrweiler , M. Sauter , and B. Schmiedeskamp , “Oxidation of Indoles by Singlet Oxygen and Dimethyldioxirane: Isolation of Indole Dioxetanes and Epoxides by Stabilization Through Nitrogen Acylation,” Tetrahedron Letters 34 (1993): 5247–5250, 10.1016/S0040-4039(00)73964-5.

[32] J. Ye , J. Wu , T. Lv , G. Wu , Y. Gao , and H. Chen , “Oxidative Rearrangement Coupling Reaction for the Functionalization of Tetrahydro‐β‐Carbolines With Aromatic Amines,” Angewandte Chemie International Edition 56 (2017): 14968–14972, 10.1002/anie.201708893. 28961354

[33] M. Ihara , K. Noguchi , K. Fukumoto , and T. Kametani , “Conversion of Indoles Into Quinolines Through the n‐1‐c‐2 Fission by Singlet‐Oxygen as a Model Experiment of Biomimetic Synthesis of Quinine Alkaloids,” Tetrahedron 41 (1985): 2109–2114, 10.1016/S0040-4020(01)96581-0.

[34] G. Srikanth , A. Ravi , A. Sebastian , et al., “Diastereoselective Synthesis of Camptothecin‐Like Scaffolds: Construction of a New Class of Pseudo‐Natural Products,” European Journal of Organic Chemistry 26 (2023): e202300080, 10.1002/ejoc.202300080.

[35] S. Liu , J. S. Scotti , and S. A. Kozmin , “Emulating the Logic of Monoterpenoid Alkaloid Biogenesis to Access a Skeletally Diverse Chemical Library,” Journal of Organic Chemistry 78 (2013): 8645–8654, 10.1021/jo401262v. 23937288 PMC3842850

[36] J. Shi , R.‐A. Wang , W. Wu , et al., “Copper‐Catalyzed Aerobic Selective Oxidation of Tetrahydrocarbolines,” Organic Letters 24 (2022): 3358–3362, 10.1021/acs.orglett.2c01059. 35503733

[37] C. S. Lancefield , L. Zhou , T. Lébl , A. M. Z. Slawin , and N. J. Westwood , “The Synthesis of Melohenine B and a Related Natural Product,” Organic Letters 14 (2012): 6166–6169, 10.1021/ol302859j. 23214465

[38] D. Sorigué , K. Hadjidemetriou , S. Blangy , et al., “Mechanism and Dynamics of Fatty Acid Photodecarboxylase,” Science 372 (2021): eabd5687, 10.1126/science.abd5687. 33833098

[39] A. Walter , W. Kuttenlochner , W. Eisenreich , C. Yao , M. Groll , and G. Storch , “Studies of α′,β′‐Epoxyketone Synthesis by Small‐Molecule Flavins and Flavoenzymes,” Angewandte Chemie International Edition 64 (2025): e202512568, 10.1002/anie.202512568. 41084917 PMC12624316

[40] X. Li , Z. Chen , W. Xu , et al., “Enzymatic Dehydrogenation and Flavin‐Mediated Photocatalytic Skeletal Rearrangement of Strictosamide, a Key Intermediate in Camptothecin Biosynthesis,” Plant Journal 126 (2026): e70823, 10.1111/tpj.70823. 41915929

[41] X. Sun , C. Li , J. Liu , R. Chen , D. Chen , and J. Dai , “Nonenzymatic Skeletal Rearrangement of 6/5/6 to 6/6/5 Ring System in the Biosynthesis of camptothecin,” Bioorganic Chemistry 178 (2026): 109950, 10.1016/j.bioorg.2026.109950. 42105527

[42] V.‐H. Bui , A. H. Vu , C. R. Buell , C. Li , L. Caputi , and T.‐T. T. Dang , bioRxiv preprint (2025), 10.1101/2025.06.24.661346.

[43] M. Dastmalchi , X. Chen , J. M. Hagel , et al., “Neopinone Isomerase Is Involved in Codeine and Morphine Biosynthesis in Opium Poppy,” Nature Chemical Biology 15 (2019): 384–390, 10.1038/s41589-019-0247-0. 30886433

[44] R. Vanholme , L. Sundin , K. C. Seetso , et al., “COSY Catalyses *Trans—cis* Isomerization and Lactonization in the Biosynthesis of Coumarins,” Nature Plants 5 (2019): 1066–1075, 10.1038/s41477-019-0510-0. 31501530

[45] V. Srivastava , P. K. Singh , A. Srivastava , and P. P. Singh , “Synthetic Applications of Flavin Photocatalysis: A Review,” RSC Advances 11 (2021): 14251–14259, 10.1039/D1RA00925G.

[46] M. Desage‐El Murr , “Nature Is the Cure: Engineering Natural Redox Cofactors for Biomimetic and Bioinspired Catalysis,” Chemcatchem 12 (2020): 53–62, 10.1002/cctc.201901642.

[47] W. Harrison , X. Huang , and H. Zhao , “Photobiocatalysis for Abiological Transformations,” Accounts of Chemical Research 55 (2022): 1087–1096, 10.1021/acs.accounts.1c00719. 35353478

[48] H. Iida , Y. Imada , and S.‐I. Murahashi , “Biomimetic Flavin‐Catalysed Reactions for Organic Synthesis,” Organic & Biomolecular Chemistry 13 (2015): 7599–7613, 10.1039/C5OB00854A. 26077635

[49] M. Toplak and R. Teufel , “Three Rings to Rule Them All: How Versatile Flavoenzymes Orchestrate the Structural Diversification of Natural Products,” Biochemistry 61 (2022): 47–56, 10.1021/acs.biochem.1c00763. 34962769 PMC8772269

[50] L. Chuang and J. Franke , Engineering Natural Product Biosynthesis Methods and Protocols, ed. E. Skellam , (Springer US, 2022), 395–420.

[51] R. S. Nett and E. S. Sattely , “Total Biosynthesis of the Tubulin‐Binding Alkaloid Colchicine,” Journal of the American Chemical Society 143 (2021): 19454–19465, 10.1021/jacs.1c08659. 34780686 PMC10824269

[52] V.‐H. Bui , T.‐A. M. Nguyen , T. Kim , et al., “Discovery of Corynanthe Synthases in Camptotheca acuminata: Medium‐Chain Dehydrogenase/Reductase‐Based Gateway to Corynanthe‐Type Compound Diversification,” Planta 262 (2025): 80, 10.1007/s00425-025-04792-0. 40804552

