# Supplementary material for: Generation of the Camptothecin Scaffold by a Flavin‐Catalyzed Photooxidative Skeletal Reorganization
Source: Angew Chem Int Ed Engl. 2026 Jul 1;65(36):e4569029. doi: 10.1002/anie.4569029 (PMC13528516; doi:10.1002/anie.4569029)
Supplement: Supplementary file 1 [file ANIE-65-e4569029-s001.pdf]

Supporting Information

Generation of the Camptothecin Scaffold by a Flavin-Catalyzed Photooxidative Skeletal Reorganization

Shenyu Liu,[a]† Zeliang Zhang,[a]† Benedikt Seligmann,[a][b] Van Hung Bui,[c] Tuan-Anh Minh Nguyen,[c] Thu-Thuy T. Dang,[c] and Jakob Franke*[a][b]

Table of Contents

Chemicals and reagents 6

General chemical methods 6

Optimization of photochemical conversion of strictosamide (5) to (3*S*)-pumiloside (6) 7

General conditions for optimization of the photoreaction of strictosamide (5) 8

O_2-dependence experiment 8

Isotope labelling experiments with $H_2{}^{18}O$ 8

Analysis of kinetic solvent isotope effects in D_2O compared to H_2O 8

UV-Vis spectroscopy 9

Testing photoreactions of strictosamide (5) with other catalysts 9

Analysis of pH dependency of intramolecular aldol reaction 9

Optimized photochemical conversion of strictosamide (5) to (3*S*)-pumiloside (6) 11

Optimized photochemical conversion of vincosamide (12) to (3*R*)-pumiloside (15) 12

Photochemical synthesis of vincosamide ketolactam / turpiniside (13) 13

Infiltration of strictosamide (5) into *N. benthamiana* leaves 14

Leaf disk experiments 14

Infiltration of *Catharanthus roseus* flower petals 14

Preparation of crude protein extracts from *Nicotiana benthamiana* 15

In vitro enzyme assays with *Nicotiana benthamiana* crude soluble protein extracts 16

Synthesis of strictosamide (5) and vincosamide (12) 17

List of Supporting Figures

Figure S1. Major background peaks of the flavin-dependent conversion of strictosamide (5) to (3*S*)-pumiloside (6) are related to the cofactors. 18
Figure S2. Possible reversible hydration of the intermediate strictosamide ketolactam (7) as an explanation for the partial incorporation of oxygen from water during isotopic labelling experiments of strictosamide (5) in $H_2{}^{18}O$. 19
Figure S3. Possible alternative mechanism for the conversion of strictosamide (5) to (3*S*)-pumiloside (6) based on incorporation of oxygen from water analogous to the proposal by Dai *et al.* for fungal indole diterpenes.[14] 20
Figure S4. Kinetic solvent isotope effect for the photooxidation of vincosamide (12) in D_2O (green curve) compared to H_2O (blue curve). 21
Figure S5. UV-Vis absorption spectra of vincosamide (12) and FMN. 22
Figure S6. Additional photosensitizers besides flavins can catalyze the conversion of strictosamide (5) to (3*S*)-pumiloside (6). 23
Figure S7. Testing the pH dependency of the conversion of strictosamide (5) to pumiloside (6). 24
Figure S8. Time course of substrate depletion and product formation during the photooxidative conversion of vincosamide (12) to (3*R*)-pumiloside (15). 25
Figure S9. Isotopic labelling experiment of vincosamide (12) in $H_2{}^{18}O$. 26
Figure S10. Comparison of MS/MS fragmentation of C-3 epimers strictosamide ketolactam (7) (top) and vincosamide ketolactam (13) (bottom) at different collision energies (CEs). 27
Figure S11. Dependence of the intramolecular aldol condensation of ketolactam intermediates on basic conditions. 28
Figure S12. Conversion of strictosamide (5) to (3*S*)-pumiloside (6) in leaf disks of camptothecin-free plants. 29
Figure S13. *In planta* conversion of strictosamide (5) to (3*S*)-pumiloside (6) in *N. benthamiana* leaves and *Catharanthus roseus* petals. 30
Figure S14. Conversion of strictosamide (5) to (3*S*)-pumiloside (6) with soluble leaf protein extracts from *N. benthamiana* compared to controls. 31
Figure S15. ^1H spectrum of (3*S*)-pumiloside (6) (DMSO-d_6, 500 MHz, 298 K). 33
Figure S16. ^{13}C spectrum of (3*S*)-pumiloside (6) (DMSO-d_6, 126 MHz, 298 K). 33
Figure S17. HSQC spectrum of (3*S*)-pumiloside (6) (DMSO-d_6, 500 MHz, 298 K). 34
Figure S18. HMBC spectrum of (3*S*)-pumiloside (6) (DMSO-d_6, 500 MHz, 298 K). 34
Figure S19. COSY spectrum of (3*S*)-pumiloside (6) (DMSO-d6, 500 MHz, 298 K). 35
Figure S20. ^1H spectrum of (3*S*)-pumiloside (6) (MeOD-d_4, 500 MHz, 298 K). 36
Figure S21. ^{13}C spectrum of (3*S*)-pumiloside (6) (MeOD-d_4, 126 MHz, 298 K). 36
Figure S22. HSQC spectrum of (3*S*)-pumiloside (6) (MeOD-d_4, 500 MHz, 298 K). 37
Figure S23. HMBC spectrum of (3*S*)-pumiloside (6) (MeOD-d_4, 500 MHz, 298 K). 37
Figure S24. COSY spectrum of (3*S*)-pumiloside (6) (MeOD-d_4, 500 MHz, 298 K). 38
Figure S25. ^1H spectrum of (3*R*)-pumiloside (15) (DMSO-d_6, 600 MHz, 298 K). 39
Figure S26. ^{13}C spectrum of (3*R*)-pumiloside (15) (DMSO-d_6, 151 MHz, 298 K). 39
Figure S27. HSQC spectrum of (3*R*)-pumiloside (15) (DMSO-d_6, 600 MHz, 298 K). 40
Figure S28. HMBC spectrum of (3*R*)-pumiloside (15) (DMSO-d_6, 600 MHz, 298 K). 40
Figure S29. COSY spectrum of (3*R*)-pumiloside (15) (DMSO-d_6, 600 MHz, 298 K). 41
Figure S30. ^1H spectrum of (3*R*)-pumiloside (15) (MeOD-d_4, 400 MHz, 298 K). 42
Figure S31. ^{13}C spectrum of (3*R*)-pumiloside (15) (MeOD-d_4, 101 MHz, 298 K). 42
Figure S32. HSQC spectrum of (3*R*)-pumiloside (15) (MeOD-d_4, 400 MHz, 298 K). 43
Figure S33. HMBC spectrum of (3*R*)-pumiloside (15) (MeOD-d_4, 400 MHz, 298 K). 43
Figure S34. COSY spectrum of (3*R*)-pumiloside (15) (MeOD-d_4, 400 MHz, 298 K). 44
Figure S35. ^1H spectrum of vincosamide ketolactam (13) (DMSO-d_6, 600 MHz, 298 K). 45
Figure S36. ^{13}C spectrum of vincosamide ketolactam (13) (DMSO-d_6, 151 MHz, 298 K). 45
Figure S37. HSQC spectrum of vincosamide ketolactam (13) (DMSO-d_6, 600 MHz, 298 K). 46
Figure S38. HMBC spectrum of vincosamide ketolactam (13) (DMSO-d_6, 600 MHz, 298 K). 46
Figure S39. COSY spectrum of vincosamide ketolactam (13) (DMSO-d_6, 600 MHz, 298 K). 47
Figure S40. ^1H spectrum of vincosamide ketolactam (13) (MeOD-d_4, 600 MHz, 298 K). 48

Figure S41. ^{13}C spectrum of vincosamide ketolactam (13) (MeOD-d_4, 151 MHz, 298 K). 48
Figure S42. HSQC spectrum of vincosamide ketolactam (13) (MeOD-d_4, 600 MHz, 298 K). 49
Figure S43. HMBC spectrum of vincosamide ketolactam (13) (MeOD-d_4, 600 MHz, 298 K). 49
Figure S44. COSY spectrum of vincosamide ketolactam (13) (MeOD-d_4, 600 MHz, 298 K). 50
Figure S45. ^{1}H spectrum of strictosamide (5) (DMSO-d_6, 600 MHz, 298 K). 51
Figure S46. ^{13}C spectrum of strictosamide (5) (DMSO-d_6, 151 MHz, 298 K). 51
Figure S47. ^{1}H spectrum of vincosamide (12) (DMSO-d_6, 400 MHz, 298 K). 52
Figure S48. ^{13}C spectrum of vincosamide (12) (DMSO-d_6, 101 MHz, 298 K). 52

List of Supporting Tables

Table S1. ^{1}H and ^{13}C NMR assignments of (3*S*)-pumiloside (6) (DMSO-d_6, 500 MHz, 298 K). 53
Table S2. ^{1}H and ^{13}C NMR assignments of (3*R*)-pumiloside (15) (DMSO-d_6, 600 MHz, 298 K). 54
Table S3. ^{1}H and ^{13}C NMR assignments of vincosamide ketolactam (13) (DMSO-d_6, 600 MHz, 298 K, or MeOD-d_4, 600 MHz, 298 K). 55
Table S4. ^{1}H and ^{13}C NMR assignment comparison between vincosamide ketolactam (13) and reported data for turpiniside[21] (MeOD-d_4). 56
Table S5. Comparison of MS/MS fragments of the C-3 epimers strictosamide ketolactam (7) and vincosamide ketolactam (13) under multiple collision energies with literature.[7] 57
Table S6. High-resolution MS/MS data of the C-3 epimers strictosamide ketolactam (7) and vincosamide ketolactam (13) under multiple collision energies. 58
Table S7. ^{1}H and ^{13}C NMR assignments of strictosamide (5) (DMSO-d_6, 600 MHz, 298 K). 59
Table S8. ^{1}H and ^{13}C NMR assignments of vincosamide (12) (DMSO-d_6, 400 MHz, 298 K). 60

Supplementary Methods

Chemicals and reagents

FMN ($FMN\text{-}Na\cdot 2H_2O$, ≥73 to ≤79%), FAD (> 94%) and riboflavin (98%) were purchased from Thermo Fisher Scientific, Tokyo Chemical Industry Co., Ltd., and Thermo Fisher Scientific, respectively. Chlorophyll (containing gum arabic and lactose as impurities) was purchased from Tokyo Chemical Industry Co., Ltd., and purified through extraction from H_2O/CH_2Cl_2 (1:1 v/v). Rose bengal was purchased from Thermo Fisher Scientific. Methylene blue was purchased from Carl Roth. *meso*-Tetraphenylporphyrin was purchased from Thermo Fisher Scientific. Deuterium oxide (99.9%) and $H_2{}^{18}O$ (97%) were purchased from Deutero GmbH. Secologanin (**18**) was isolated from leaves of *Lonicera tatarica* as described previously.[28] Strictosidine (**16**) was also produced as described previously.[28] Chemicals not mentioned specifically were purchased from Fisher Scientific, Carl Roth, or Sigma Aldrich.

General chemical methods

Analytical and semipreparative LC-MS analyses were performed on an Agilent Infinity II 1260 system consisting of a G7167A autosampler, G7116A column thermostat, G7111B quaternary pump, G7110B make-up pump, G7115A diode array detector, G1364F fraction collector, and G6125B single quadrupole mass spectrometer equipped with an ESI source (positive mode, 4000 V, 12 L/min drying gas, 350 °C gas temperature). Samples were separated on a C18 column (Poroshell 120 EC-C18, 100 × 4.6 mm, 2.7 µm). The column temperature was set to 50 °C. As mobile phase, solvent A (water with 0.1% (v/v) formic acid) and solvent B (acetonitrile with 0.1% (v/v) formic acid) were used. The following gradient at a flow rate of 1.0 mL/min was used: 0-1 min, 5% B; 1-11 min, 5-55% B; 11-12 min, 55-90% B; 12-14 min, 90% B; 14-14.5 min, 90-5% B; 14.5-16 min, 5% B. Mass spectra were obtained in scan mode in a range of *m*/*z* 200-600 at a fragmentor voltage of 100 V.

Automated flash chromatography was performed on a Biotage Isolera One with the stationary phases, solvents and gradients described in the corresponding method sections.

NMR spectra were recorded using Bruker Ultrashield 400, Ultrashield 500 or Ascend 600 MHz spectrometers operating at 400, 500 and 600 MHz for ^1H NMR and at 101, 126 and 151 MHz for ^{13}C NMR. Methanol-d_4, or DMSO-d_6 were used as solvents. Chemical shifts were referenced relative to the residual solvent signals (Methanol-d_4: δ_H = 3.31 ppm, δ_C = 49.00 ppm; DMSO-d_6: δ_H = 2.50 ppm, δ_C = 39.52 ppm) and expressed in δ values (ppm), with coupling constants reported in Hz. Analysis was conducted with TopSpin (Version 4.4.1) or MestReNova (Version 14.2).

HRMS measurements were carried out on a Waters Acquity UPLC coupled to a Waters QTof Premier mass spectrometer.

HR-MS/MS measurements were performed on an Orbitrap Q Exactive Plus mass spectrometer (Thermo Fisher) at a resolution of 35,000 (operated in full MS mode), AGC target and maximum injection time were set to 3e6 and 50 ms, respectively, and at a resolution of 17,500 (operated in PRM mode), AGC target and maximum injection time were set to 2e5 and 100 ms, respectively. CEs were set as described in individual experiments. The heated ESI (electrospray-ionization) source was operated at 0 eV CID, sheath gas flow 65, auxiliary gas flow 20, sweep gas flow 4, spray voltage 3.5 kV, capillary temperature 320 °C, S-lens RF level 50.0 and aux gas heater 500 ˚C.

Optimization of photochemical conversion of strictosamide (5) to (3*S*)-pumiloside (6)

Photochemical reactions were performed in a 1.5-mL microcentrifuge tube without lid. Strictosamide (**5**) was dissolved in MeOH to a 10 mM stock solution. FMN and FAD were prepared in buffer at 10 mM, and riboflavin was prepared in DMSO at 10 mM.

Flavin cofactor assays

Strictosamide (**5**) (4 µL, 10 mM stock, 4 nmol, 1.0 equiv.) was combined with different flavin cofactors (FMN, FAD, or riboflavin; 10 µL, 10 mM stock, 10 nmol, 2.5 equiv.) in 50 mM Tris buffer (pH 8.8, 186 µL). Reactions were incubated at 37 °C with shaking at 500 rpm for 20 h and analyzed by LC-MS.

pH dependency assays

Strictosamide (**5**) (4 µL, 10 mM stock, 4 nmol, 1.0 equiv.) and FMN solution (40 µL, 10 mM stock, 40 nmol, 10.0 equiv.) were added to 50 mM Tris buffers at pH 7.5, 8.0, or 8.8, or 50 mM CHES buffer at pH 9.5 (156 µL). Reaction mixtures were incubated at 37 °C with shaking at 500 rpm for 20 h and analyzed by LC-MS.

Light dependency assays

Strictosamide (**5**) (8 µL, 10 mM stock, 8 nmol, 1.0 equiv.) and FMN solution (20 µL, 10 mM stock, 20 nmol, 2.5 equiv.) were added to 50 mM Tris buffers at pH 8.8 (372 µL) in the dark. Reaction mixtures were first incubated at 37 °C with shaking at 500 rpm for 3 h in the dark. The mixtures were placed under fume hood illumination for next 3 h. After all substrates were mixed, samples were taken at hourly periods and analyzed by LC-MS.

General conditions for optimization of the photoreaction of strictosamide (5)

Strictosamide (**5**) (100 µL, 10 mmol/L in MeOH, 1 µmol, 1.0 equiv.) and FMN (15 µmol/mL in 50 mM Tris-HCl buffer pH = 8.8) were dissolved in Tris-HCl buffer (pH = 8.8, 900 µL) in a 10 mL glass vial. Then, the reaction was stirred at room temperature under illumination by a 4.5 W white LED light. 10 µL of the reaction solution was taken for LC-MS analysis every hour until the reaction was completed.

O_2-dependence experiment

Strictosamide (**5**) (100 µL, 10 mmol/L in MeOH, 1 µmol, 1.0 equiv.) and FMN (3.3 µL, 15 µmol/mL in 50 mM Tris-HCl buffer pH = 8.8, 5 mol%) were dissolved in Tris-HCl buffer (pH = 8.8, 900 µL) in a 10 mL Schlenk flask. The resulting mixture was treated with freeze-vacuum-thaw cycles to remove oxygen and then maintained in an atmosphere of N_2. Then, the reaction was stirred at room temperature under illumination by a 4.5 W white LED light for 2 h. 10 µL of the reaction solution was taken for LC-MS analysis.

Isotope labelling experiments with $H_2{}^{18}O$

To prepare a suitable $H_2{}^{18}O$-containing buffer, 10.2 mg Tris and 2.5 mg Tris-HCl were dissolved in 1.5 mL of $H_2{}^{18}O$. The pH was then measured using a pH indicator paper and the volume was brought up to 2 mL with $H_2{}^{18}O$.

Strictosamide (**5**) (1.0 mg, 2.0 µmol, 1.0 equiv.) and FMN (0.2 mg, 20 mol%) were dissolved in MeOH (0.2 mL). Then, Tris-HCl buffer (pH = 9, 0.3 mL) prepared from $H_2{}^{18}O$ was added. The resulting mixture was stirred at room temperature under illumination by a 4.5 W white LED light for 2 h. 10 µL of the reaction solution was taken for LC-MS analysis.

Alternatively, vincosamide (**12**) (1.0 mg, 2.0 µmol, 1.0 equiv.) and FMN (0.2 mg, 20 mol%) were dissolved in MeOH (0.2 mL). Then, Tris-HCl buffer (pH = 9, 0.3 mL) prepared from $H_2{}^{18}O$ was added. The resulting mixture was stirred at room temperature under illumination by a 4.5 W white LED light for 2 h. 10 µL of the reaction solution was taken for LC-MS analysis.

Analysis of kinetic solvent isotope effects in D_2O compared to H_2O

Vincosamide (**12**) (100 µL, 10 mmol/L in MeOH, 1 µmol, 1.0 equiv.) and FMN (15 µmol/mL in 50 mM Tris-HCl buffer pH = 8.8, prepared with either D_2O or H_2O) were dissolved in 50 mM Tris-HCl buffer (pH = 8.8, 900 µL, prepared with either D_2O or H_2O) in a 10 mL glass

vial. Then, the reaction was stirred at room temperature under illumination by a 4.5 W white LED light. 10 µL of the reaction solution was taken for LC-MS analysis at 0 min, 5 min, 10 min, 20 min, 30 min, 40 min, 50 min and 60 min. The reactions were performed in triplicate.

UV-Vis spectroscopy

UV-Vis absorption spectra were recorded with a Jasco spectrophotometer V-750 from 300 nm to 650 nm. First, a blank measurement was carried out with only Tris buffer (50 mM, pH = 8.8)/MeOH (4:1 v/v) in a cuvette. Then, FMN-Na (0.1 mM in Tris buffer (50 mM, pH = 8.8)/MeOH (4:1 v/v)) and vincosamide (**12**) (0.5 mM in 50 mM Tris buffer(pH = 8.8)/MeOH (4:1 v/v)) were measured separately. Lastly, a mixture of FMN-Na (0.1 mM) and vincosamide (0.5 mM) in Tris buffer (50 mM, pH = 8.8)/MeOH (4:1 v/v) was recorded.

Testing photoreactions of strictosamide (5) with other catalysts

Strictosamide (**5**) (100 µL, 10 mmol/L in MeOH, 1 µmol, 1.0 equiv.) and rose bengal or methylene blue (1 mmol/L in 50 mM Tris-HCl buffer pH = 8.8, 50 µL, 5 mol%) were dissolved in Tris-HCl buffer (pH = 8.8, 900 µL) in a 10 mL glass vial. Then, the reaction was stirred at room temperature under illumination by a 4.5 W white LED light for 30 min. The reaction mixture was subsequently adjusted to pH 10 – 11 by adding aq. NaOH (10 M) and stirred for 1 h. 10 µL of the reaction solution was taken for LC-MS analysis.
Alternatively, strictosamide (**5**) (100 µL, 10 mmol/L in MeOH, 1 µmol, 1.0 equiv.) and *meso*-tetraphenylporphyrin (1 mmol/L in tetrahydrofuran, 50 µL, 5 mol%) or chlorophyll (1 mmol/L in CH_2Cl_2, 200 µL, 20 mol%) were dissolved in CH_2Cl_2 (900 µL) in a 10 mL glass vial. Then, the reaction was stirred at room temperature under illumination by a 4.5 W white LED light for 2 h. The reaction mixture was subsequently adjusted to pH 10 – 11 by adding aq. NaOH (10 M) and stirred for 1 h. 10 µL of the reaction solution was taken for LC-MS analysis.

Analysis of pH dependency of intramolecular aldol reaction

Vincosamide ketolactam (**13**) (100 µL, 3 mmol/L in buffer (50 mM Tris-HCl, pH = 8.8)/MeOH, 2:1 v/v) was dissolved in 50 mM Tris-HCl buffer (pH = 8.8, 900 µL). Then, the reaction mixture was acidified by adding either HCO_2H or aq. HCl (6 M) to pH 2 – 3, or alternatively basified by adding aq. NaOH (10 M) to pH 10 – 11. The resulting mixtures were stirred at room temperature for 2 h and then used for LC-MS analysis.

Strictosamide (**5**) (100 µL, 10 mmol/L in MeOH, 1 µmol, 1.0 equiv.) and FMN (3.3 µL, 15 µmol/mL in 50 mM Tris-HCl buffer pH = 8.8, 5 mol%) were dissolved in Tris-HCl buffer (pH = 8.8, 900 µL) in a 10 mL glass vial. Then, the reaction was stirred at room temperature under illumination by a 4.5 W white LED light for 1 h. After that, the reaction mixture was adjusted to pH 10 – 11 by adding aq. NaOH (10 M) and stirred for 1 h. 10 µL of the reaction solution was taken for LC-MS analysis.

Optimized photochemical conversion of strictosamide (5) to (3*S*)-pumiloside (6)

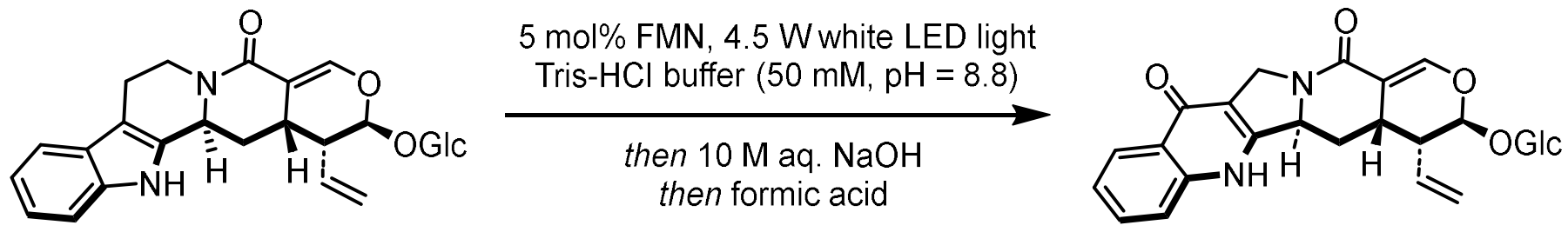

Strictosamide (**5**) → (3*S*)-Pumiloside (**6**)

Strictosamide (**5**) (5 mg, 0.01 mmol, 1.0 equiv.) and FMN (33 µL, 15 µmol/mL in 50 mM Tris-HCl buffer pH = 8.8, 5 mol%) were dissolved in Tris-HCl buffer (pH = 8.8, 2 mL) and MeOH (0.5 mL). The resulting mixture was stirred at room temperature under illumination by a 4.5 W white LED light for 1 h. Then, the mixture was adjusted to pH 10 – 11 by adding aq. NaOH (10 M) and stirred for 30 min. Subsequently, formic acid was added dropwise to neutralize the mixture. Afterwards, the mixture was concentrated and redissolved in MeOH/H_2O (1 mL, 1/1 v/v) for semipreparative LC-MS. The conditions and gradients for compound isolation were:

Kinetex 5 µm C18 100 Å,<br>250 × 10.0 mm	A: H_2O+0.1% FA<br>B: MeCN+0.1% FA<br>Flowrate: 5 mL/min	15-25% B 0-10 min<br>25-95% B 10-10.5 min<br>95-95% B 10.5-12.5 min<br>95-15% B 12.5-12.6 min<br>15% B 12.6-15 min	3.8 mg,<br>76%

HR-ESI-MS: found $[M+H]^+$ 513.1884 (calcd. for $C_{26}H_{29}O_9N_2^+$, 513.1868)

NMR data for (3*S*)-pumiloside (**6**) is shown in Figure S15-Figure S24 and Table S1.

Optimized photochemical conversion of vincosamide (12) to (3*R*)-pumiloside (15)

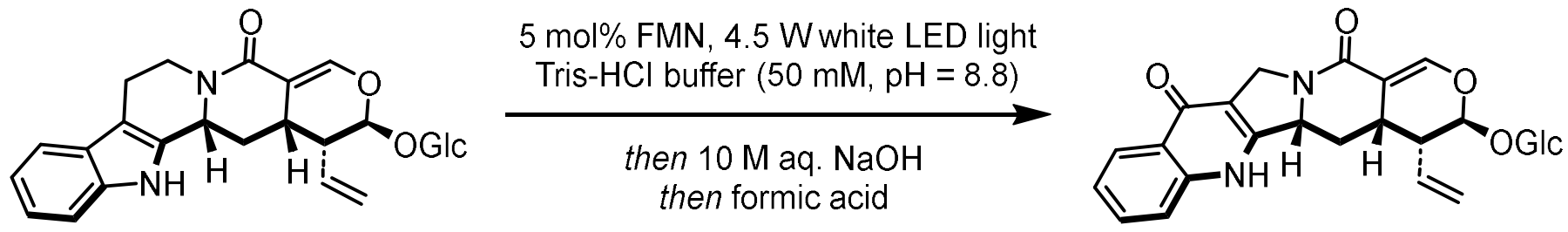

Vincosamide (**12**) (5 mg, 0.01 mmol, 1.0 equiv.) and FMN (33 µL, 15 µmol/mL in 50 mM Tris-HCl buffer pH = 8.8, 5 mol%) were dissolved in Tris-HCl buffer (pH = 8.8, 2 mL) and MeOH (1.0 mL). The resulting mixture was stirred at room temperature under illumination by a 4.5 W white LED light for 1 h. Then, the mixture was adjusted to pH 10 – 11 by adding aq. NaOH (10 M) and stirred for 30 min. Subsequently, formic acid was added dropwise to neutralize the mixture. Afterwards, the mixture was concentrated and redissolved in MeOH/H_2O (1 mL, 1/1 v/v) for semipreparative LC-MS. The conditions and gradients for compound isolation were:

Kinetex 5 µm C18 100 Å, 250 × 10.0 mm	A: H_2O+0.1% FA<br>B: MeCN+0.1% FA<br>Flowrate: 5 mL/min	15-25% B 0-10 min<br>25-95% B 10-10.5 min<br>95-95% B 10.5-12.5 min<br>95-15% B 12.5-12.6 min<br>15% B 12.6-15 min	4.3 mg, 86%

HR-ESI-MS: found $[M+H]^+$ 513.1867 (calcd. for $C_{26}H_{29}O_9N_2^+$, 513.1868)

NMR data for (3*R*)-pumiloside (**15**) is shown in Figure S25-Figure S34 and Table S2.

Photochemical synthesis of vincosamide ketolactam / turpiniside (13)

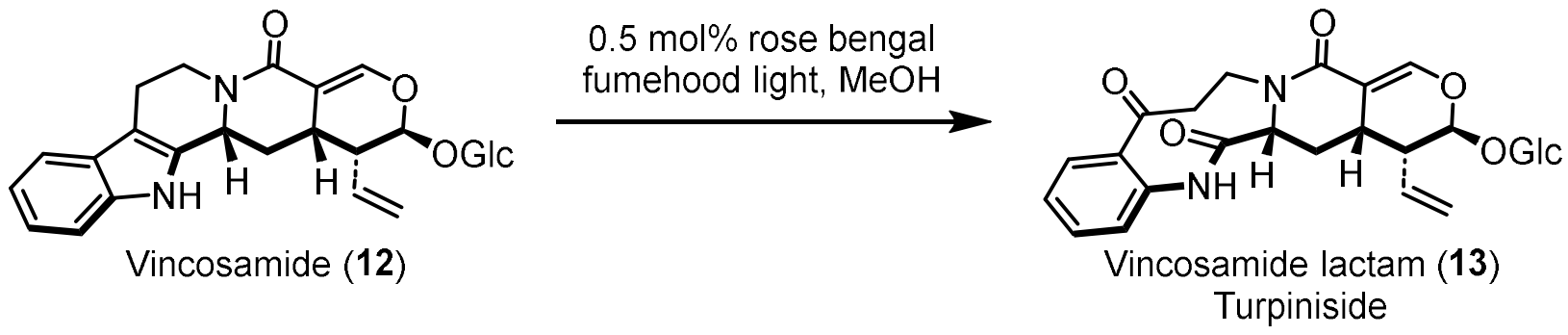

Vincosamide (**12**) (5 mg, 0.01 mmol, 1.0 equiv.) and rose bengal (50 µL of a 1 mM solution in MeOH, 0.05 µmol, 0.005 equiv.) were dissolved in MeOH (500 µL). The resulting mixture was stirred at room temperature under illumination from the fume hood for 4 days. Vincosamide ketolactam / turpiniside (**13**) was obtained by semipreparative LC-MS. The conditions and gradients for compound isolation were:

Kinetex 5 µm C18 100 Å, 250 × 10.0 mm	A: H_2O+0.1% FA<br>B: MeCN+0.1% FA<br>Flowrate: 5 mL/min	15-23% B 0-8 min<br>23-95% B 8-8.5 min<br>95-95% B 8.5-10.5 min<br>95-15% B 10.5-10.6 min<br>15% B 10.6-13 min	4.8 mg, 96%

HR-ESI-MS: found $[M+H]^+$ 531.1979 (calcd. for $C_{26}H_{31}O_{10}N_2^+$, 531.1973)

NMR data for vincosamide ketolactam / turpiniside (**13**) is shown in Figure S35-Figure S44 and Table S3-Table S4.

Infiltration of strictosamide (5) into *N. benthamiana* leaves

Nicotiana benthamiana LAB plants were grown from seeds in a greenhouse under 11–16 h of light per day at 21–23 °C as previously described.[50] Leaves of 4–5-week-old plants were infiltrated with strictosamide (**5**) solution (100 μM in H_2O containing 1% DMSO) or control solution (H_2O with 1% DMSO) using a 1 mL syringe. Infiltrated leaves were harvested 3 days post infiltration. Five leaf disks per treatment were collected using a cork borer (no. 5, 10-mm diameter), lyophilized, and extracted with 800 μL MeOH. Extracts were analyzed by LC-MS as described above.

Leaf disk experiments

Leaf disk infiltration assays were performed with leaf disks of *Nicotiana benthamiana* and the monoterpene indole alkaloid-producing species *Mitragyna speciosa*, *Gelsemium sempervirens*, and *Rauvolfia serpentina*. Five leaf disks per treatment were collected using a 10-mm cork borer and incubated in 48-well plates containing 500 μL strictosamide (**5**) solution (100 μM in H_2O with 1% DMSO) or control solution (H_2O with 1% DMSO). Leaf disks were harvested 3 days after incubation, lyophilized, and extracted with 500 μL MeOH. Extracts were analyzed by LC-MS as described previously.[52] EICs corresponding to m/z 499 and m/z 513 were monitored for strictosamide (**5**) and (3*S*)-pumiloside (**6**), respectively.

Infiltration of *Catharanthus roseus* flower petals

In planta conversion of strictosamide (**5**) to (3*S*)-pumiloside (**6**) was evaluated in *Catharanthus roseus* petals. For petal infiltration, a small hole was made with a needle before infiltration. *C. roseus* petals were infiltrated with strictosamide (**5**) solution (100 μM in H_2O with 1% DMSO) or control solution (H_2O with 1% DMSO) using a 1 mL syringe. Tissues were harvested 24 h after infiltration, lyophilized, and extracted with MeOH at a 1:10 (w/v) ratio. Extracts were analyzed by LC-MS as described previously.[42] EICs corresponding to m/z 499 and m/z 513 were monitored for strictosamide (**5**) and (3*S*)-pumiloside (**6**), respectively.

Preparation of crude protein extracts from *Nicotiana benthamiana*

The extraction of crude soluble leaf proteins from *Nicotiana benthamiana* was performed according to a previous report by Nett and Sattely.[51] Mature leaves of 10 *Nicotiana benthamiana* plants (4 weeks old) were harvested and homogenized using a mortar and pestle with liquid N_2 and a small scoop of silica and polyvinylpolypyrrolidone. The tissue powder was suspended in 150 mL ice-cooled Tris buffer (100 mM Tris·HCl pH 7.5, 10% glycerol, 1 mM phenylmethylsulfonyl fluoride, 10 mM β-mercaptoethanol and 3 tablets of EDTA-free protease inhibitor cocktail tablets). The suspension was incubated at 4 °C for 40 mins. The solid residue was removed by filtration with Miracloth. The filtrate was first pre-centrifuged at 4,000 x *g* for 10 min at 4 °C to remove the majority of insoluble material and further centrifuged at 30,000 x *g* for 15 min at 4 °C. The supernatant was removed and aliquoted into pre-cooled microcentrifuge tubes and stored at −80 °C prior to use.

<u>Determination of enzyme concentration</u>

1 mL of crude soluble leaf protein extracts was added into a pre-weighed 2-mL microcentrifuge tube followed by 1 mL methanol. The solution was vigorous vortexed and centrifuged at 21,300 x *g* for 15 min. The supernatant was carefully removed and the pellet was dried under fine vacuum. The pellet was weighed and used for calculation of enzyme concentration.

<u>Preparation of boiled enzyme extract</u>

1 mL of crude soluble leaf protein extract was boiled at 100 °C for 20 mins in a 2-mL microcentrifuge tube. The boiled protein extract was cooled in an ice bath prior to use.

<u>Preparation of filtered enzyme extract</u>

16 mL crude soluble leaf protein extract was evenly split into four samples of 4 mL each and loaded on centrifugal concentrators (Vivaspin Turbo 4, MWCO 5 kDa, PES). The concentrators were centrifuged at 4,000 x *g* for 60 min. The filtrates were collected, combined and stored at −80 °C prior to use.

***In vitro* enzyme assays with *Nicotiana benthamiana* crude soluble protein extracts**

In vitro enzyme assays were performed in 50 mM Tris-HCl pH 7.5 buffer in a final volume of 100 µL. Reactions contained 0.5 µg/µL of crude protein, and 100 µM of strictosamide (**5**). Strictosamide (**5**) was pre-dissolved in a solution of methanol and 50 mM Tris-HCl pH 7.5 buffer (1:1, v/v) at 10 mM stock concentration. The reactions were incubated at 30 °C, 500 rpm for 16 hours under a plant growth lamp or in the dark. The reactions were quenched by adding 100 µL MeOH and vigorously vortexed. After vortexing, the reactions were centrifuged at 21,300 x *g* at room temperature for 15 min to remove the precipitated proteins. The supernatants were filtered through a 0.22 µm PTFE syringe filter into LC–MS vials and diluted with four extra volumes of methanol supplemented with 0.1% of total volume of formic acid for LC-MS analysis. LC–MS analysis was performed using a Thermo Scientific UltiMate 3000 ultra-HPLC system coupled to a Bruker Impact II ultra–high-resolution QToF mass spectrometer (Bruker Daltonics). Samples were separated on a C18 column (Phenomenex Kinetex XB-C18, 100 × 2.1 mm, 2.6 µm). The column temperature was set to 40 °C. As mobile phase, solvent A (water with 0.1% (v/v) formic acid) and solvent B (acetonitrile with 0.1% (v/v) formic acid) were used. The following gradient at a flow rate of 0.6 mL/min was used: 0-1 min, 10% B; 1-6 min, 10-50% B; 6-6.5 min, 50-95% B; 6.5-7.5 min, 95% B; 7.5-8 min, 95-10% B; 8-10 min, 5% B. Mass spectra were obtained in scan mode in a range of *m*/*z* 200-800.

Synthesis of strictosamide (5) and vincosamide (12)

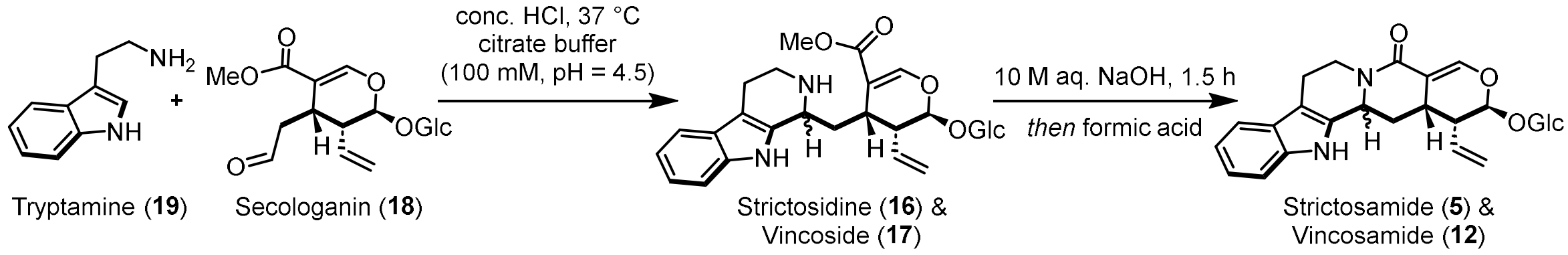

Secologanin (**18**) (200 mg, 0.438 mmol, 1.0 equiv.) and tryptamine (**19**) (70.2 mg, 0.438 μmol, 1.0 equiv.) were dissolved in citrate buffer (100 mM, pH = 4.5, 2 mL), and concentrated HCl (43 μL) was added. The reaction mixture was stirred at 37 °C for 70 h. The reaction mixture was then basified to pH 10 – 12 using aq. NaOH (10 M) and stirred at room temperature for an additional 1.5 h, then formic acid was added dropwise to neutralize the mixture to pH ~7. The reaction mixture was concentrated under reduced pressure and redissolved in MeOH for isolation. The conditions and gradients for isolation were:

Biotage	Sfär C18 D Duo<br>100 Å<br>30 μm, 12 g	A: H_2O+0.1% FA<br>B: MeCN+0.1% FA<br>Flowrate: 12 mL/min	10-10% B (3 CV)<br>10-25% B (1 CV)<br>25-40% B (8 CV)<br>40-90% B (1 CV)<br>90-90% B (2 CV)	strictosamide:<br>94.5 mg, 43%;<br>vincosamide:<br>101.3 mg,<br>46%

HR-ESI-MS: Strictosamide found $[M+H]^+$ 499.2073 (calcd. for $C_{26}H_{31}O_8N_2^+$, 499.2075)

HR-ESI-MS: Vincosamide found $[M+Na]^+$ 521.1879 (calcd. for $C_{26}H_{30}O_8N_2Na^+$, 521.1894)

NMR data for strictosamide (**5**) is shown in Figure S45-Figure S46 and Table S7.

NMR data for vincosamide (**12**) is shown in Figure S47-Figure S48 and Table S8.

Supporting Figures

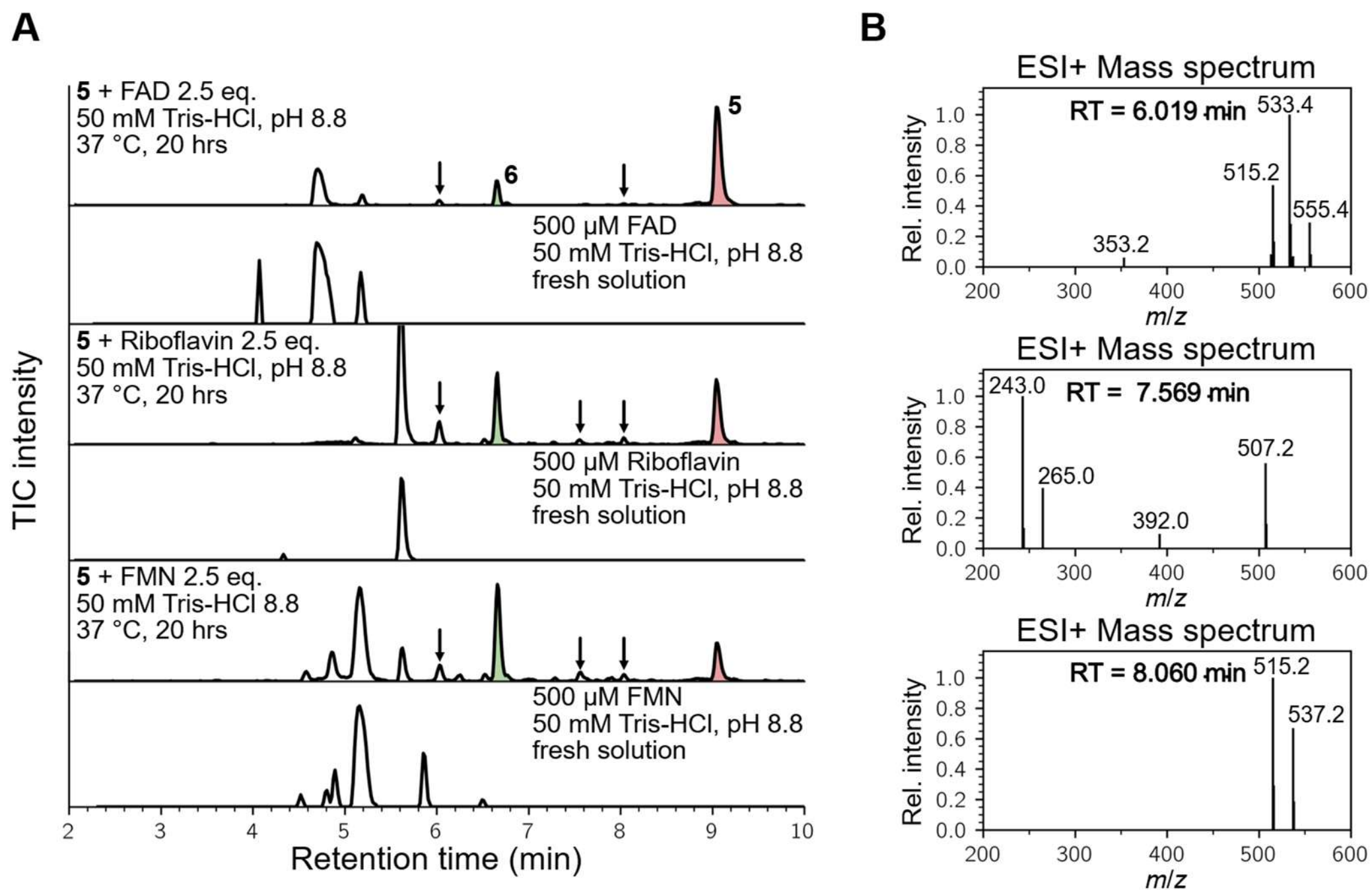

Figure S1. Major background peaks of the flavin-dependent conversion of strictosamide (**5**) to (3*S*)-pumiloside (**6**) are related to the cofactors.

A) Comparison of chromatograms shown in Figure 2 to fresh solutions of the cofactors flavin mononucleotide (FMN), riboflavin, and flavin adenine dinucleotide (FAD) without added substrate. Minor peaks indicated with arrows were consistently observed during flavin-dependent conversions of strictosamide (**5**).

B) Electrospray ionization (ESI) mass spectra (positive mode) of minor peaks indicated with arrows in panel A.

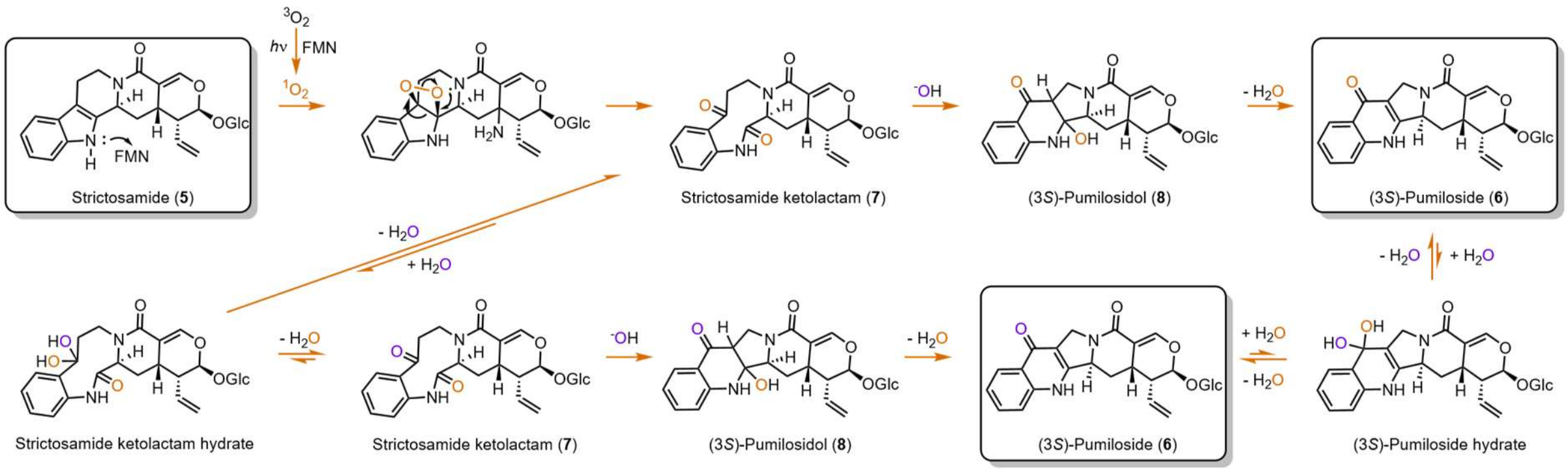

Figure S2. Possible reversible hydration of the intermediate strictosamide ketolactam (**7**) as an explanation for the partial incorporation of oxygen from water during isotopic labelling experiments of strictosamide (**5**) in $H_2{}^{18}O$.

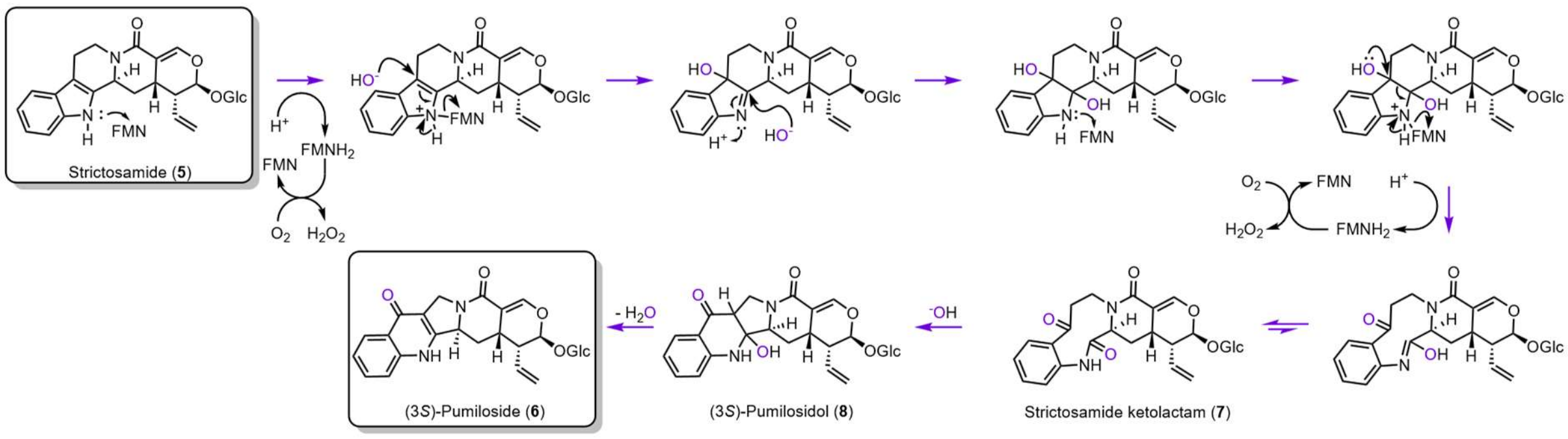

Figure S3. Possible alternative mechanism for the conversion of strictosamide (**5**) to (3*S*)-pumiloside (**6**) based on incorporation of oxygen from water analogous to the proposal by Dai *et al.* for fungal indole diterpenes.[14]

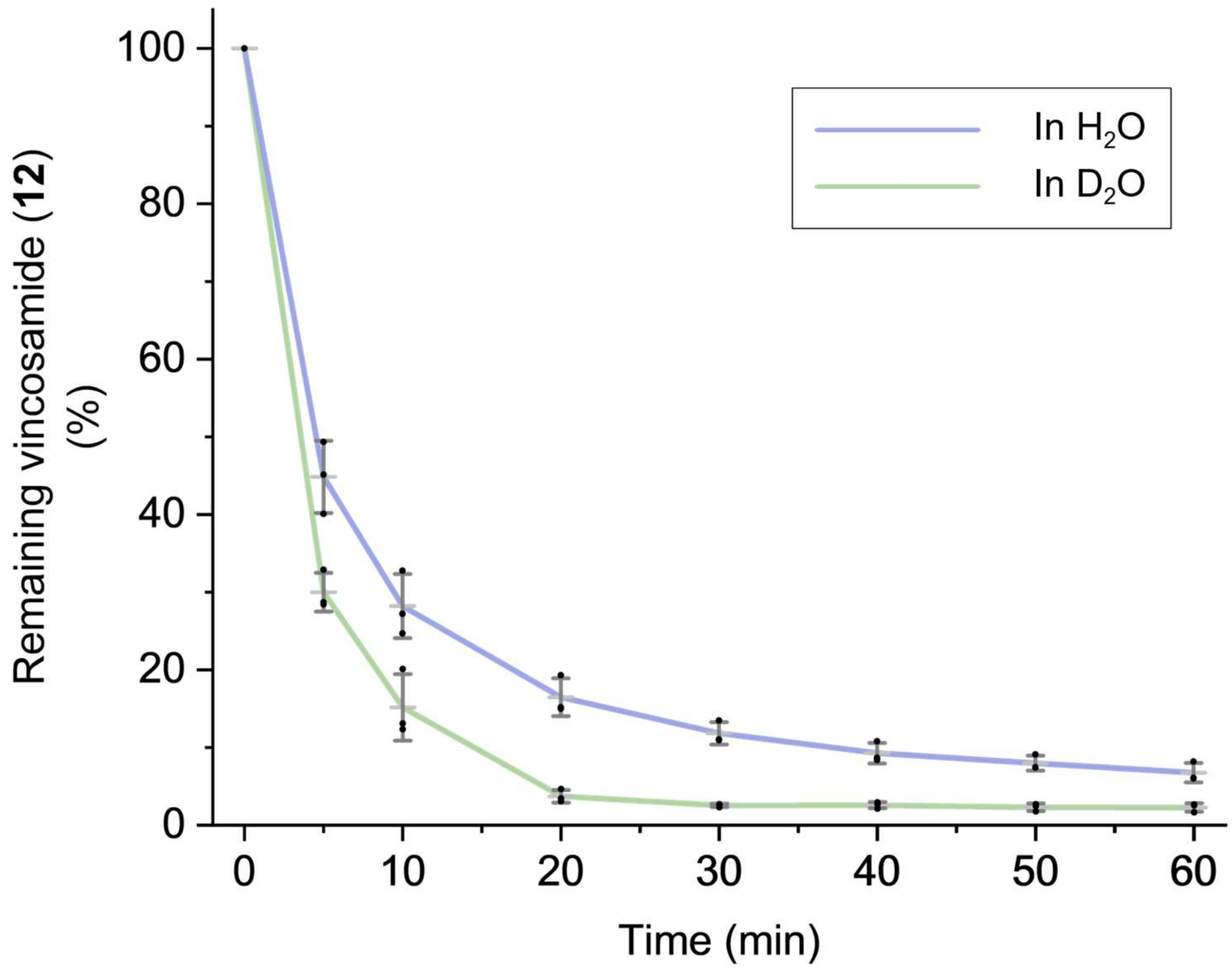

Figure S4. Kinetic solvent isotope effect for the photooxidation of vincosamide (**12**) in D_2O (green curve) compared to H_2O (blue curve).

Vincosamide (**12**) levels were calculated using the peak area from LC-MS analysis relative to the sum of the peak areas of vincosamide (**12**), vincosamide ketolactam (**13**) and (3*R*)-pumiloside (**15**). Data show mean values ± standard deviation and data points of three independently set up reactions.

For strictosamide (**5**) as a substrate, a similar, but weaker acceleration effect was observed.

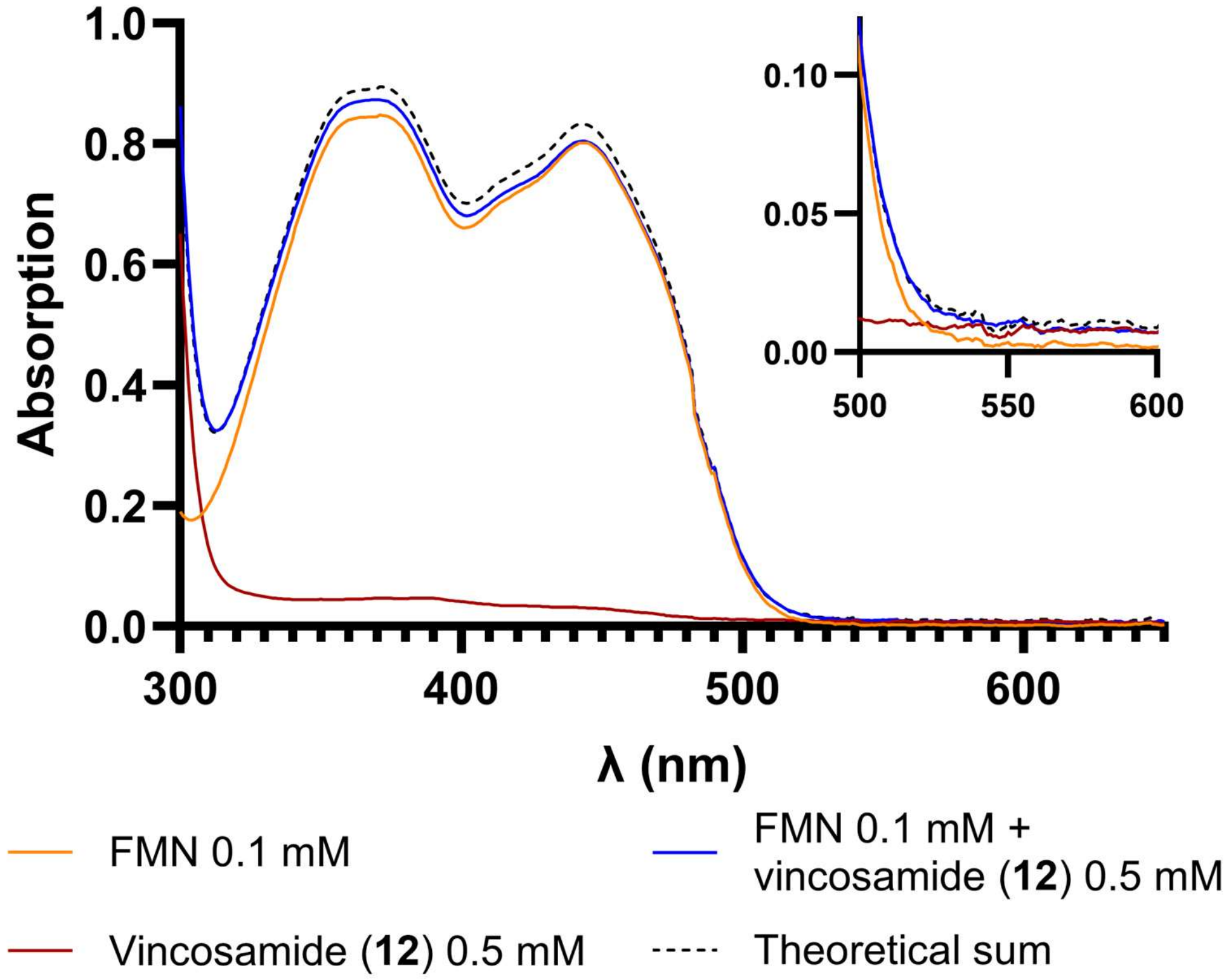

Figure S5. UV-Vis absorption spectra of vincosamide (**12**) and FMN.

No difference between the combined measurement of vincosamide (**12**) and FMN (blue line) versus the theoretical sum of the individual components (dashed black line) that would be indicative of an EDA complex was observed in the 500-600 nm region (inset).

Similar results were observed for strictosamide (**5**) as a substrate.

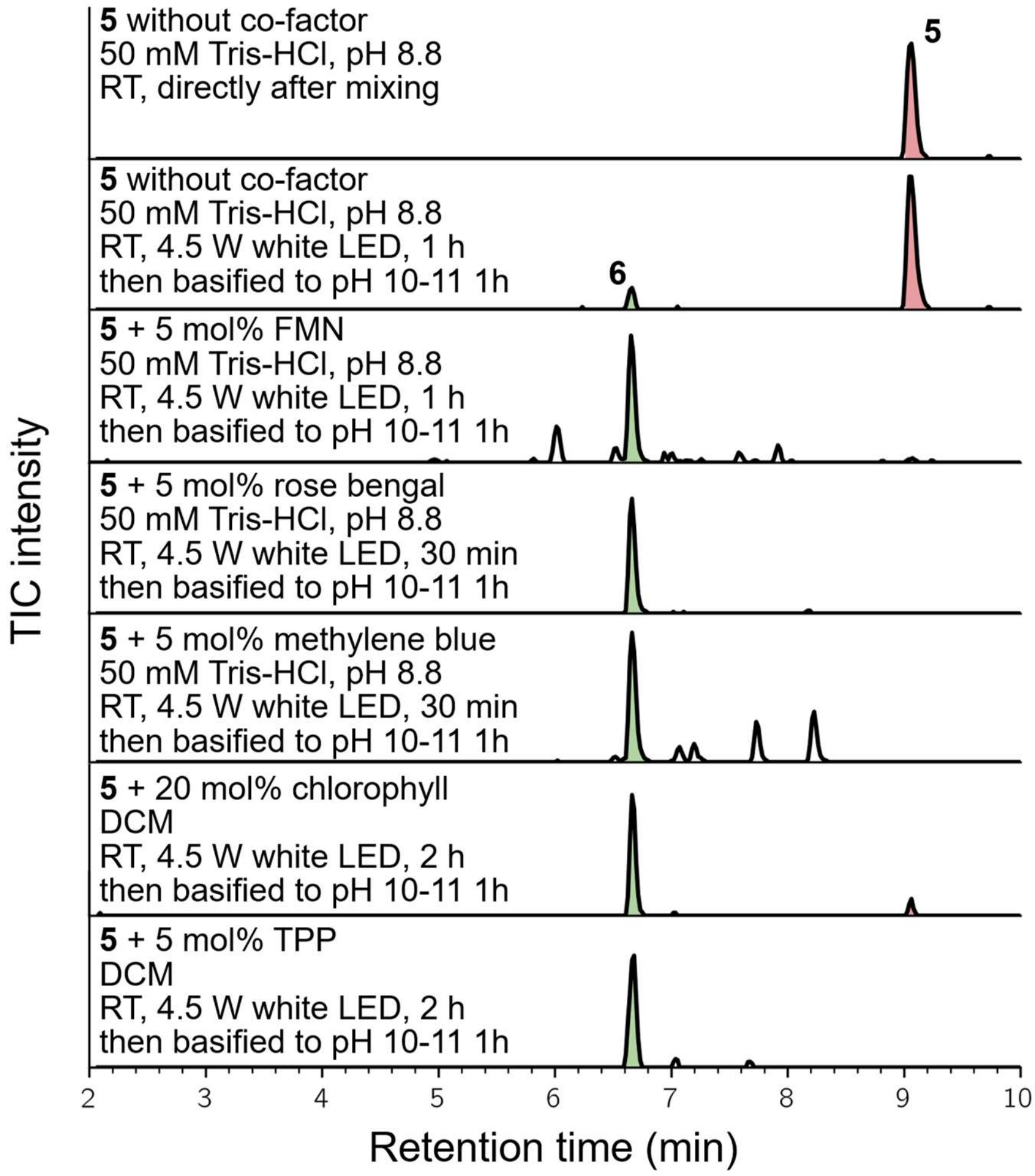

Figure S6. Additional photosensitizers besides flavins can catalyze the conversion of strictosamide (**5**) to (3*S*)-pumiloside (**6**).

In addition to flavin mononucleotide (FMN), rose bengal, methylene blue, chlorophyll and *meso*-tetraphenylporphyrin (TPP) were tested. Data shown are total ion chromatograms from LC-MS analysis.

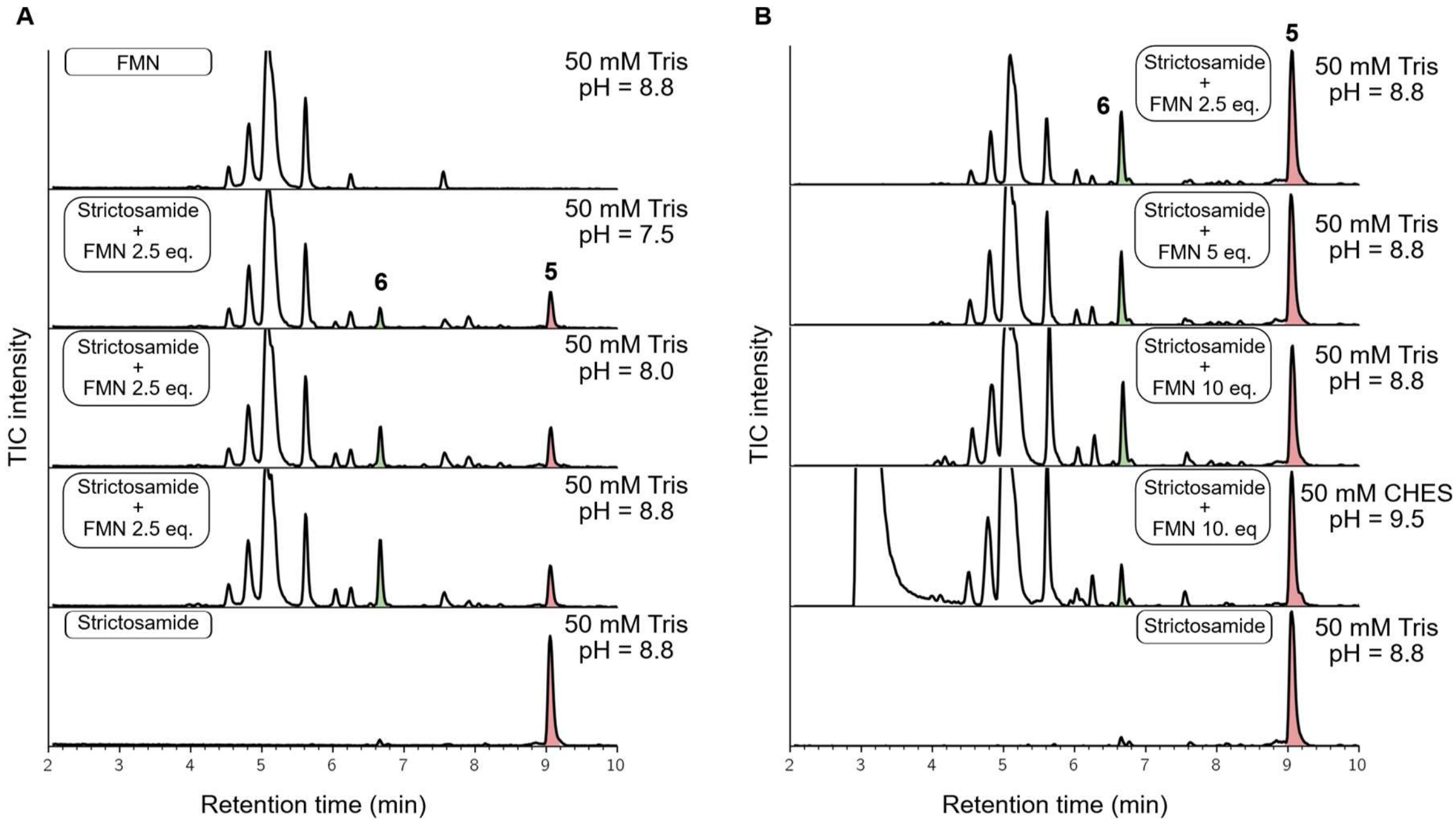

Figure S7. Testing the pH dependency of the conversion of strictosamide (**5**) to pumiloside (**6**).

A) Strictosamide (**5**) (0.2 mmol), FMN (0.5 mmol), pH from 7.5 to pH 8.8, at 37 °C, 500 rpm, 20 hrs.

B) Strictosamide (**5**) (0.2 mmol), FMN (0.5 to 2 mmol), pH 8.8 or pH 9.5, at 37 °C, 500 rpm, 20 hrs.

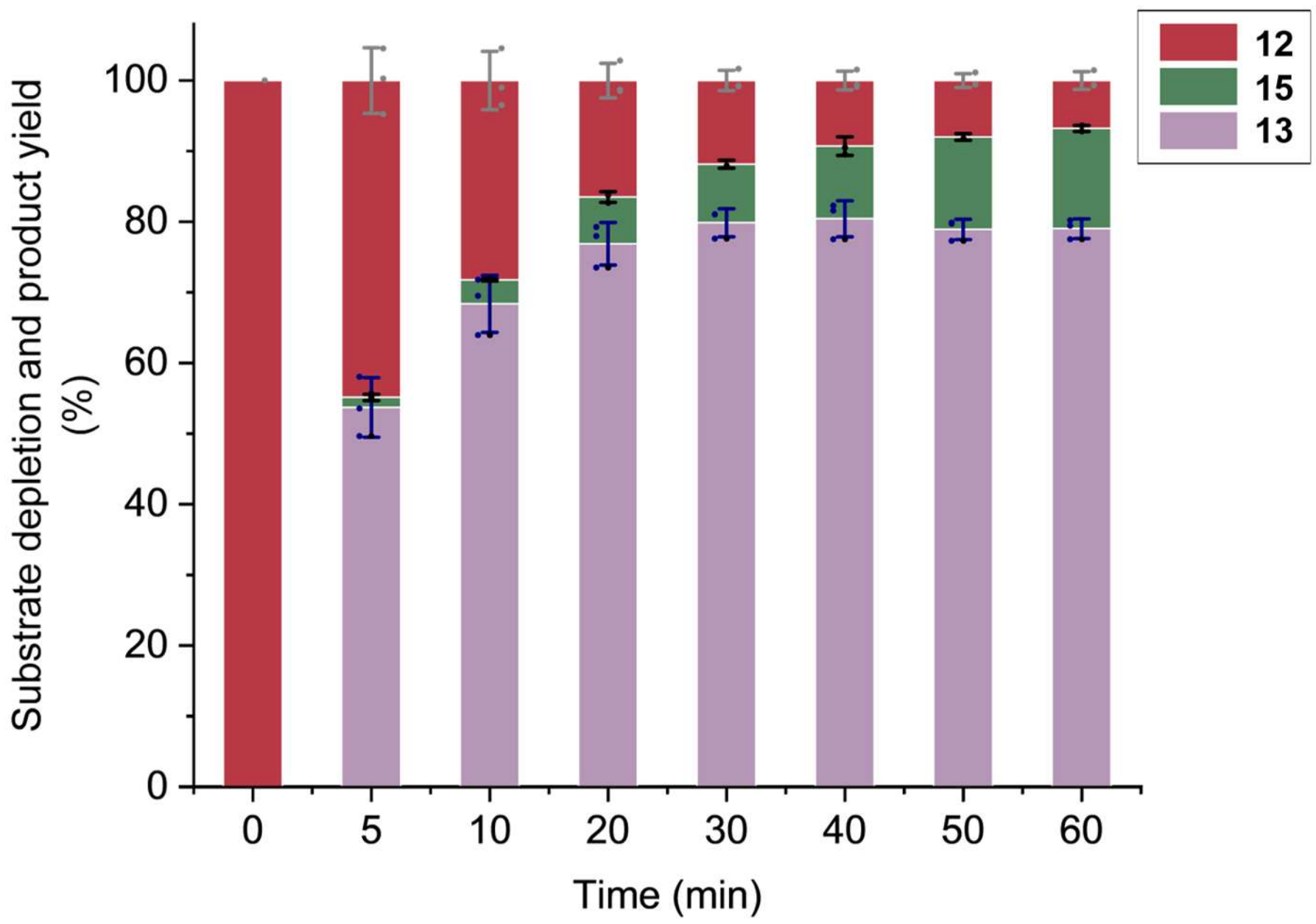

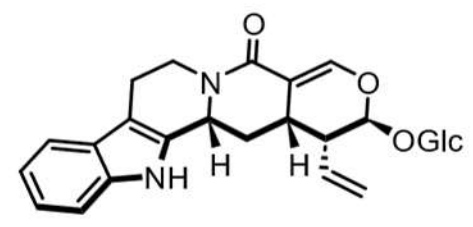

Vincosamide (**12**)

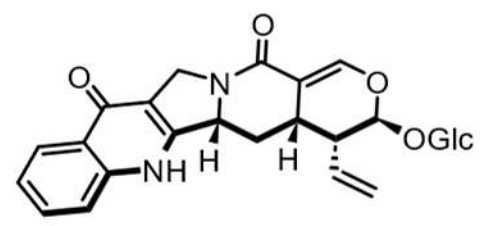

(3*R*)-Pumiloside (**15**)

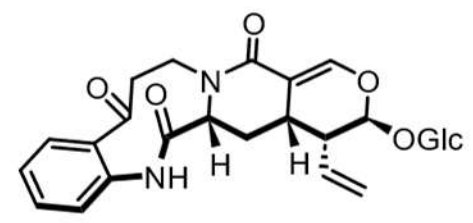

Vincosamide ketolactam (**13**)

Figure S8. Time course of substrate depletion and product formation during the photooxidative conversion of vincosamide (**12**) to (3*R*)-pumiloside (**15**).

Vincosamide (**12**), vincosamide ketolactam (**13**) and (3*R*)-pumiloside (**15**) levels were calculated using the respective peak areas from LC-MS analysis relative to the sum of the three peak areas. Data show mean values ± standard deviation and data points of three independently set up reactions.

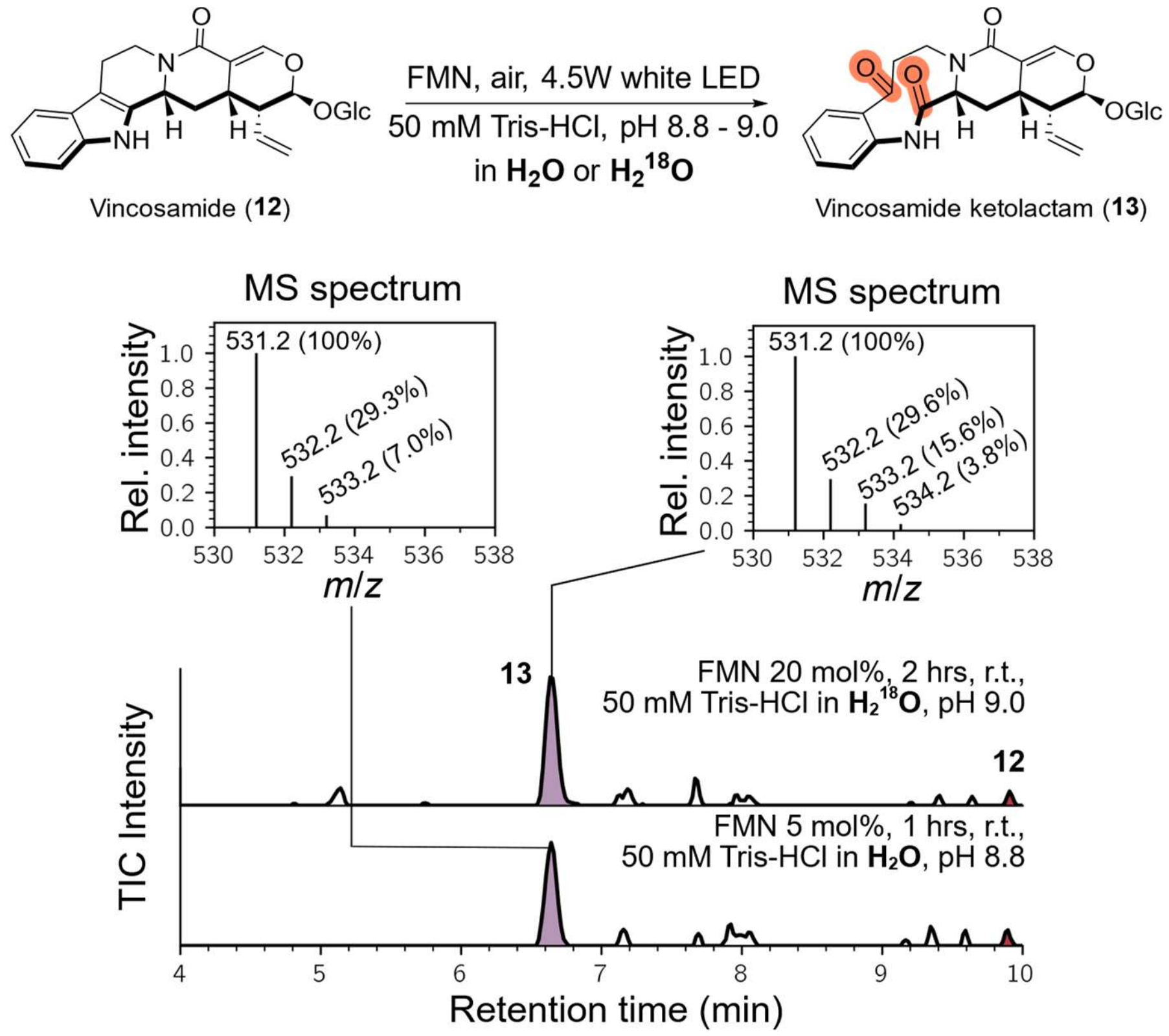

Figure S9. Isotopic labelling experiment of vincosamide (**12**) in $H_2{}^{18}O$.

MS, mass spectrometry.

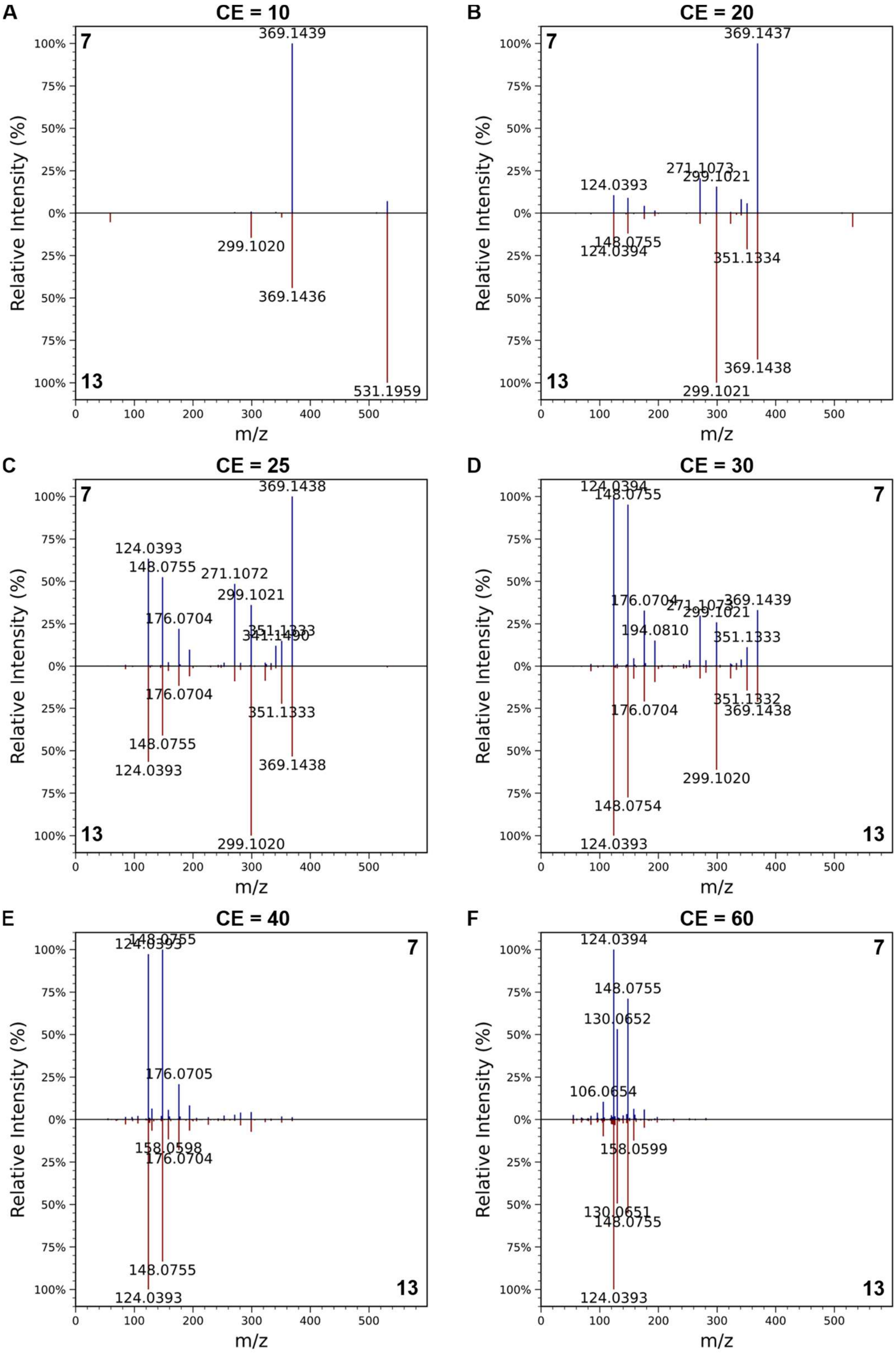

Figure S10. Comparison of MS/MS fragmentation of C-3 epimers strictosamide ketolactam (**7**) (top) and vincosamide ketolactam (**13**) (bottom) at different collision energies (CEs).

Precursor ion = 531 (calc. [M+H$^+$] 531.1979, $C_{26}H_{31}N_2O_{10}^+$). A) CE = 10 eV; B) 20 eV; C) 25 eV; D) 30 eV; E) 40 eV; F) 50 eV. High-resolution *m*/*z* values with a relative intensity above 10% are shown.

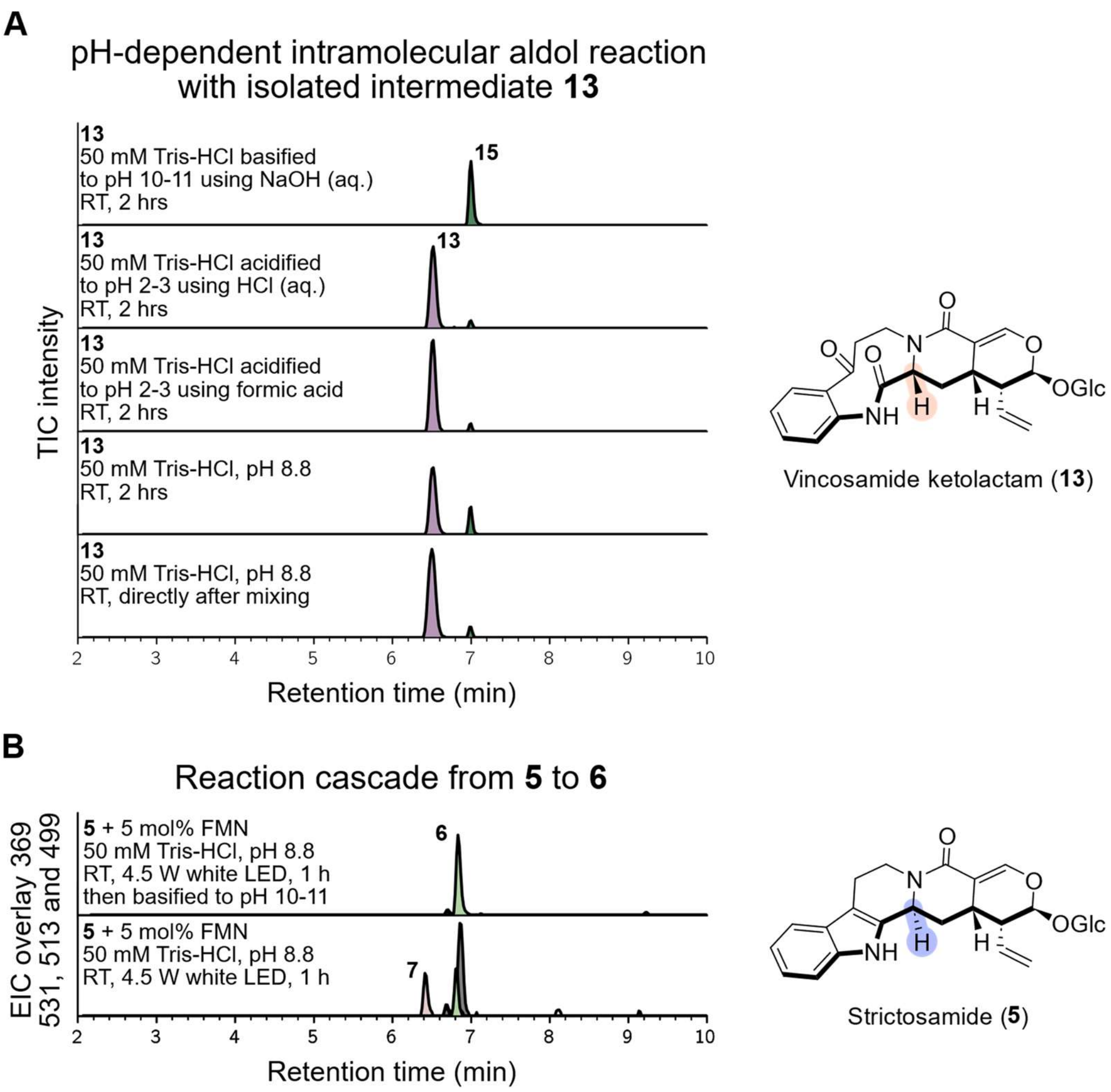

Figure S11. Dependence of the intramolecular aldol condensation of ketolactam intermediates on basic conditions.

A) LC-MS total ion chromatograms of isolated vincosamide ketolactam (**13**) treated at different pH values. Only at pH 10-11 complete conversion to (3*R*)-pumiloside (**15**) was observed. No aldol reaction was observed under acidic conditions.

B) Final basification of the photooxidation reaction mixture of strictosamide (**5**) helps to drive the aldol condensation step to pumiloside (**6**) to completion.

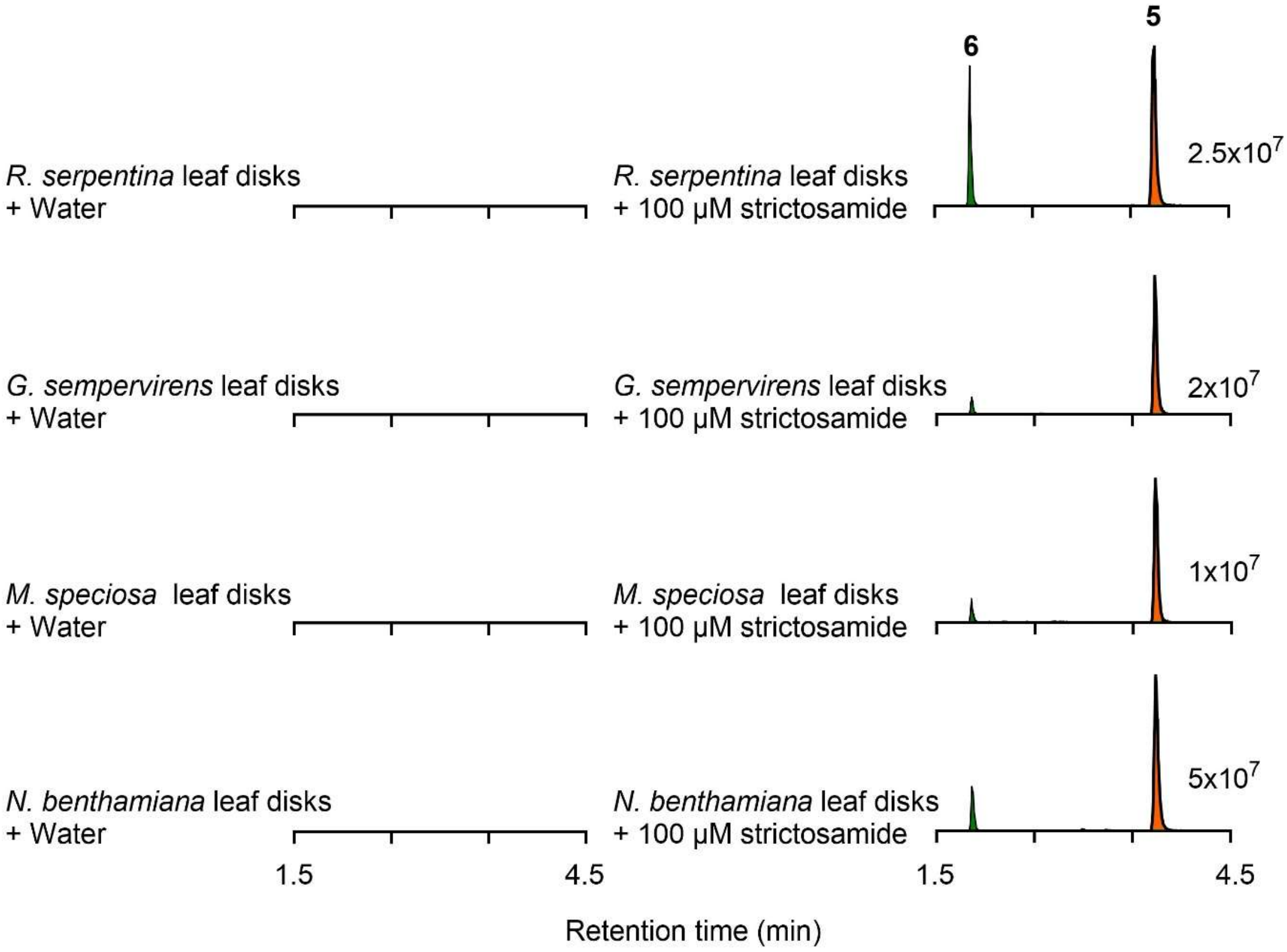

Figure S12. Conversion of strictosamide (**5**) to (3*S*)-pumiloside (**6**) in leaf disks of camptothecin-free plants.

Leaf disk infiltration assays (72 h) were performed to assess the formation of (3*S*)-pumiloside (**6**) from strictosamide (**5**) in the camptothecin-free species *Mitragyna speciosa*, *Gelsemium sempervirens*, and *Rauvolfia serpentina* in comparison to the model plant *N. benthamiana*. Extracted ion chromatogram (EIC) overlays corresponding to *m*/*z* 499 (strictosamide, **5**) and *m*/*z* 513 ((3*S*)-pumiloside, **6**) are shown.

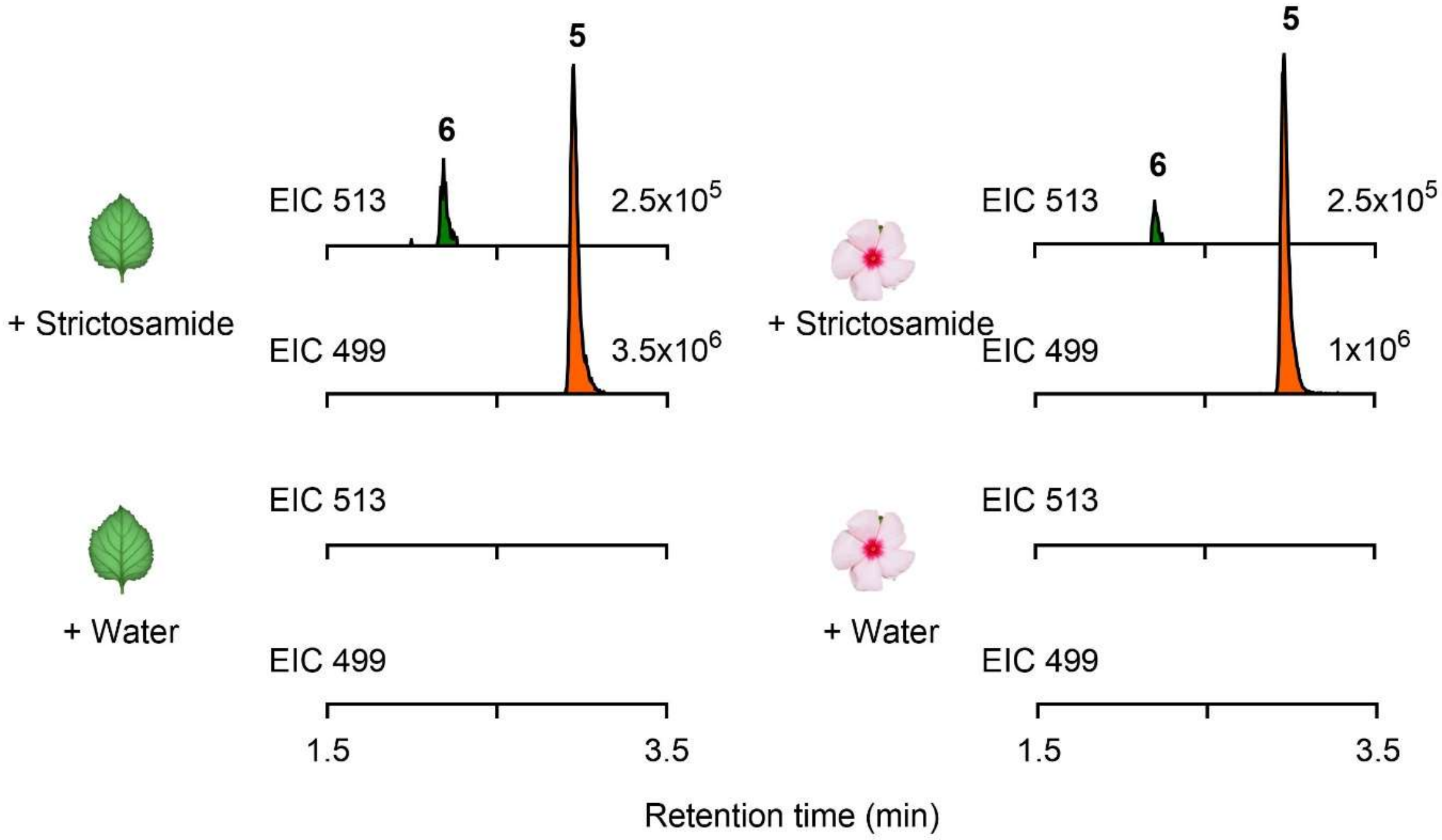

Figure S13. *In planta* conversion of strictosamide (**5**) to (3*S*)-pumiloside (**6**) in *N. benthamiana* leaves and *Catharanthus roseus* petals.

Results were evaluated 24 h after infiltration. This experiment was conducted independently in a different laboratory compared to the results for *N. benthamiana* shown in Figure 5.

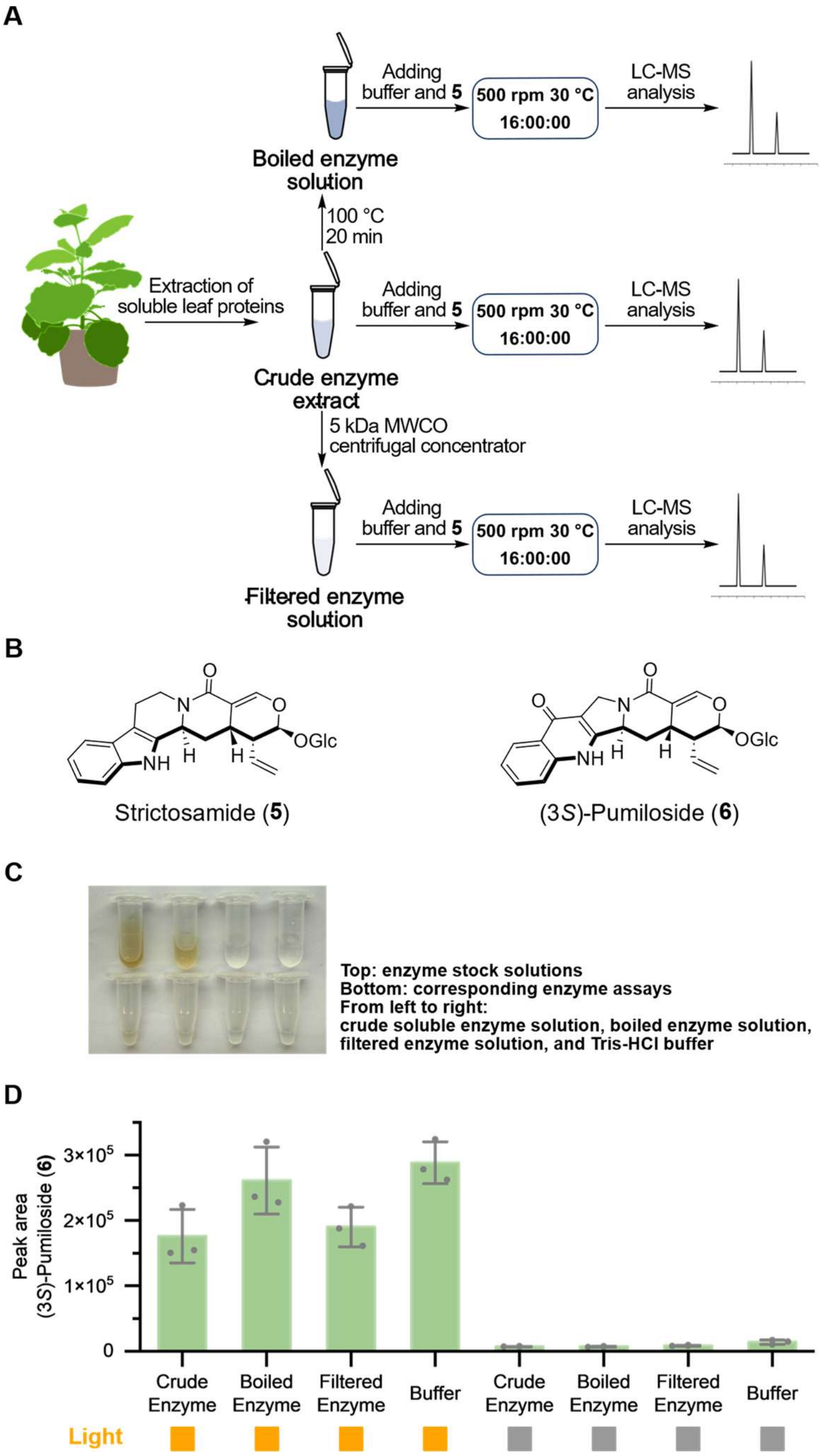

Figure S14. Conversion of strictosamide (**5**) to (3*S*)-pumiloside (**6**) with soluble leaf protein extracts from *N. benthamiana* compared to controls.

A) Experimental workflow. A crude extract of soluble proteins was obtained from *N. benthamiana* leaves; as controls, this enzyme extract was either boiled or filtered with a 5 kDa molecular weight cut-off (MWCO) centrifugal concentrator. Alternatively, buffer was used as an additional control.

B) Structures of strictosamide (**5**) and (3*S*)-pumiloside (**6**).

C) Photograph of different protein samples used in this experiment. The crude protein extract and the boiled extract exhibited a yellow-brown coloration, whereas the filtrate and buffer were colorless.

D) Comparable levels of (3*S*)-pumiloside (**6**) were obtained for the crude enzyme extract and all controls, suggesting that no endogenous soluble enzyme from *N. benthamiana* is essential for this reaction. As only soluble proteins were extracted, it remains possible that an endogenous membrane-bound enzyme can carry out this reaction *in planta*. The bar plot shows mean values ± standard deviation and data points of three reactions.

Nicotiana_benthamiana icon by Connor-Tansley https://github.com/Ctansley is licensed under CC-BY 4.0 Unported https://creativecommons.org/licenses/by/4.0/.

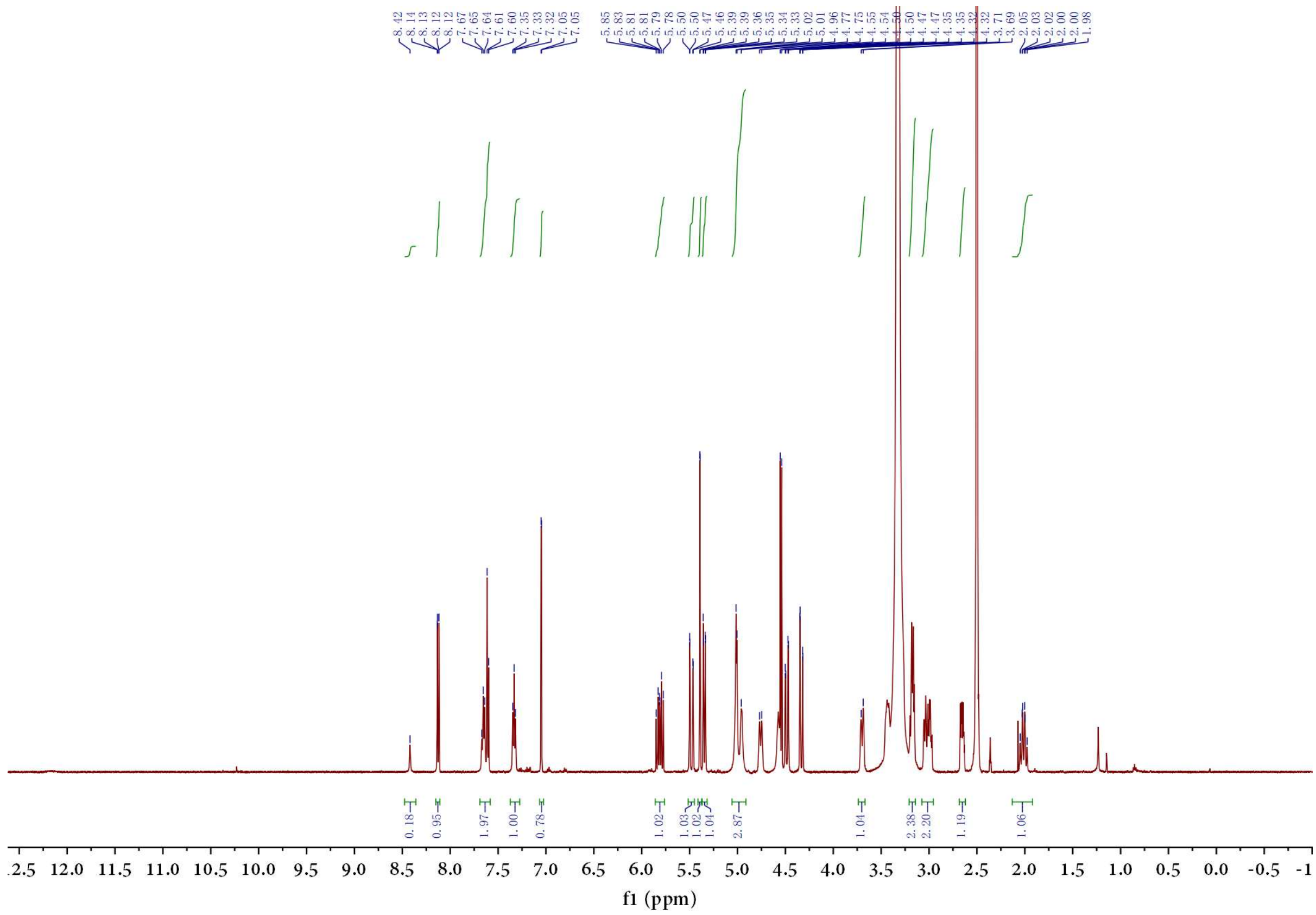

Figure S15. ^{1}H spectrum of (3*S*)-pumiloside (**6**) (DMSO-d_6, 500 MHz, 298 K).

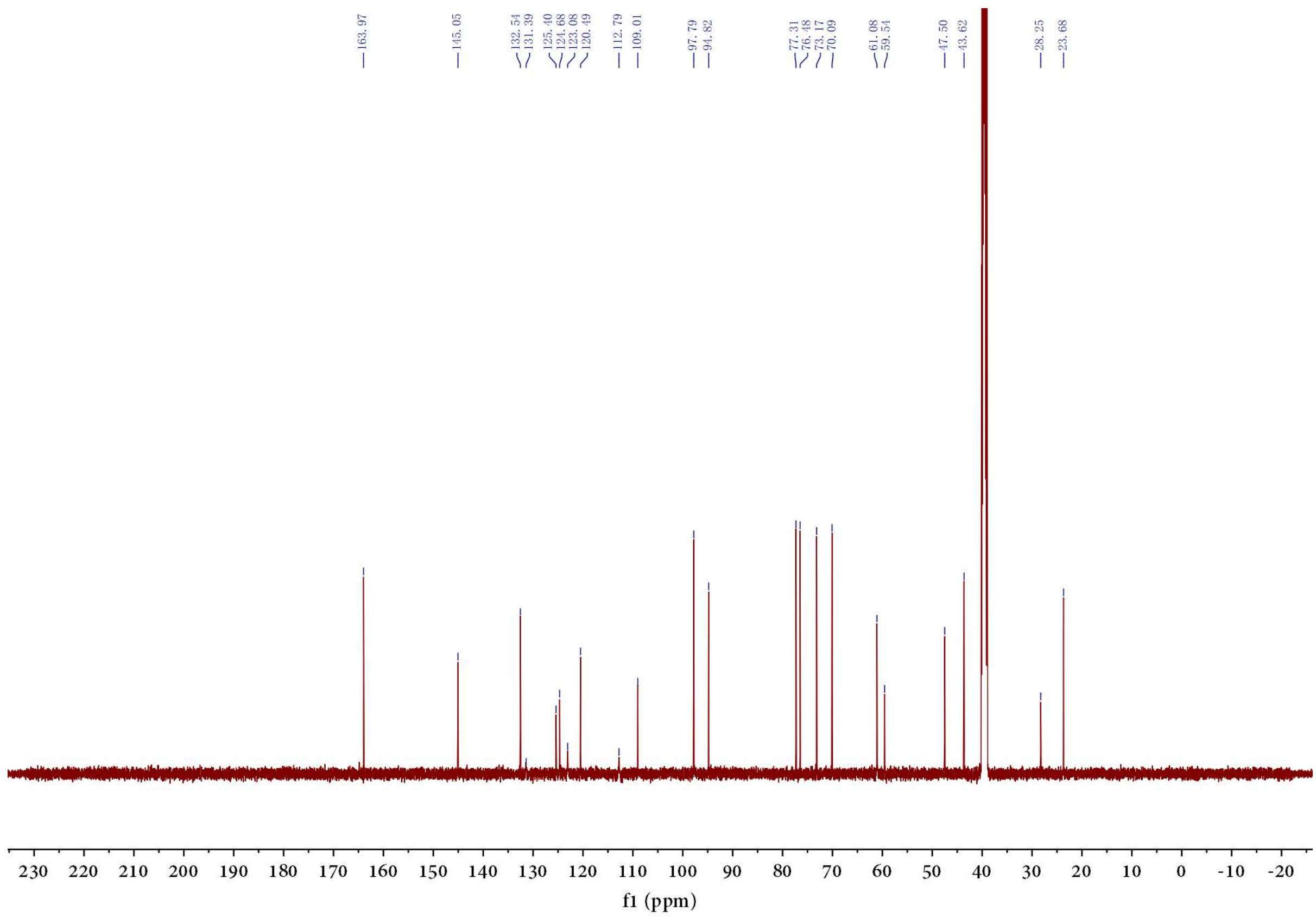

Figure S16. ^{13}C spectrum of (3*S*)-pumiloside (**6**) (DMSO-d_6, 126 MHz, 298 K).

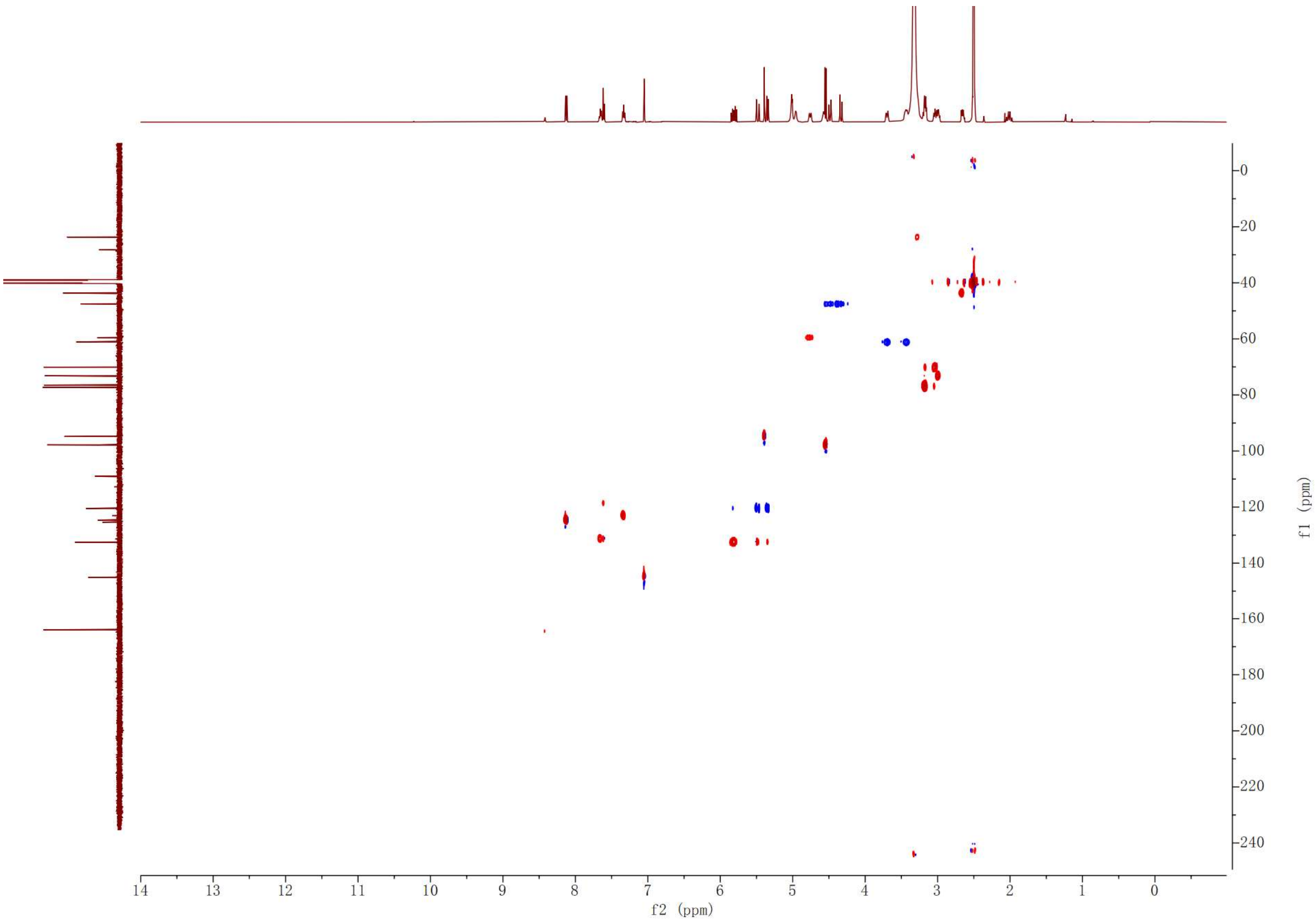

Figure S17. HSQC spectrum of (3*S*)-pumiloside (**6**) (DMSO-d$_6$, 500 MHz, 298 K).

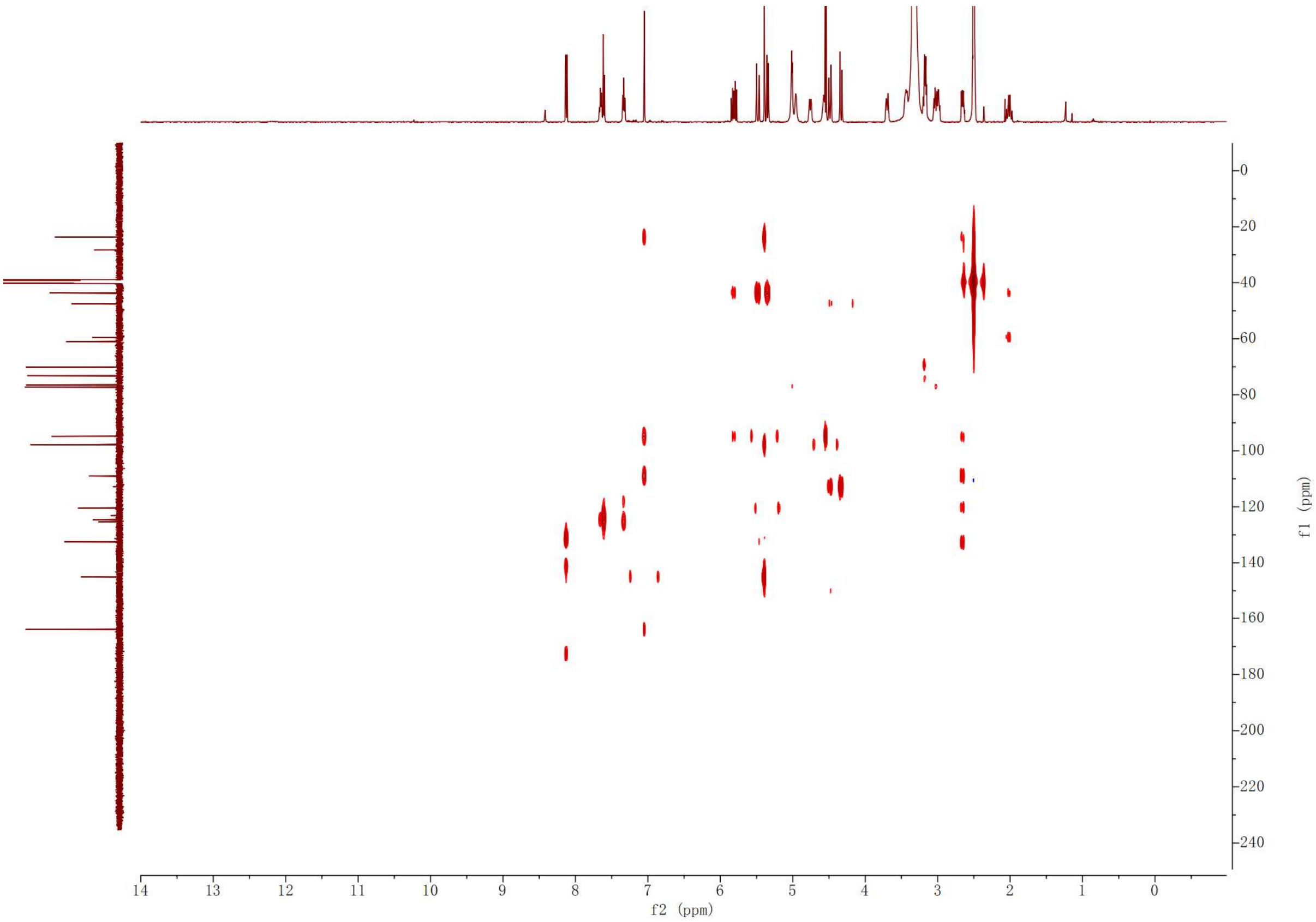

Figure S18. HMBC spectrum of (3*S*)-pumiloside (**6**) (DMSO-d$_6$, 500 MHz, 298 K).

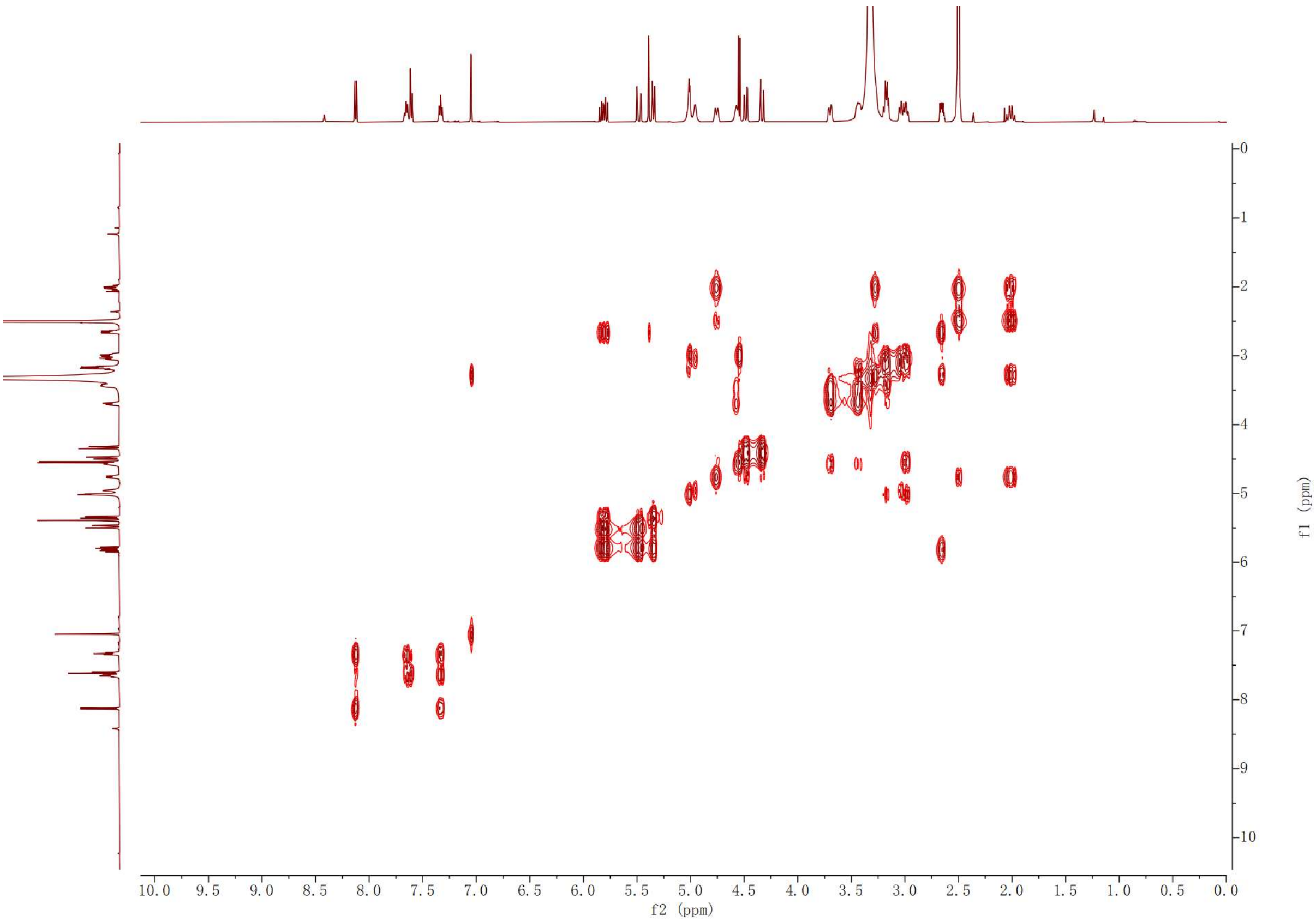

Figure S19. COSY spectrum of (3*S*)-pumiloside (**6**) (DMSO-d6, 500 MHz, 298 K).

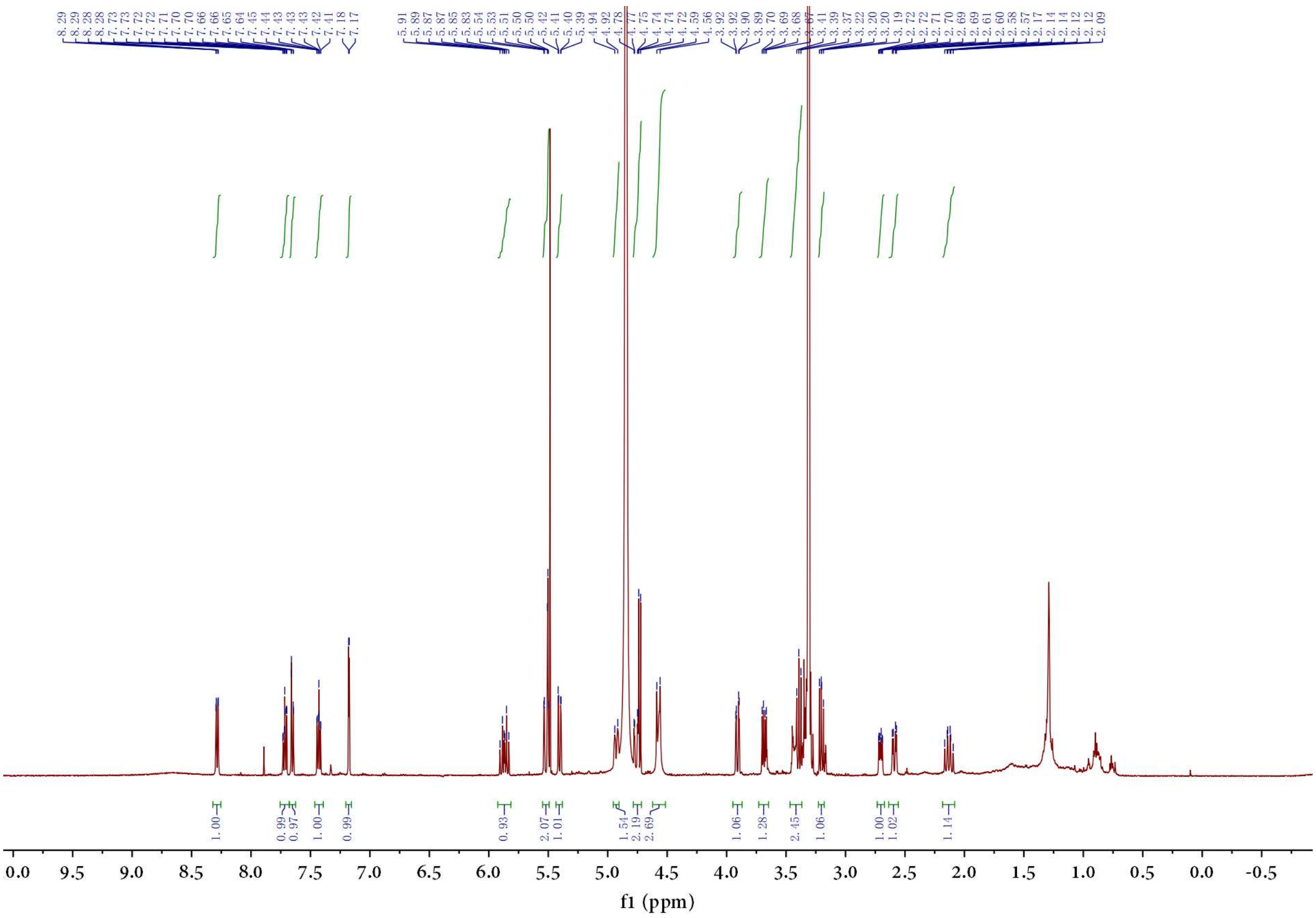

Figure S20. ^{1}H spectrum of (3*S*)-pumiloside (**6**) (MeOD-d_4, 500 MHz, 298 K).

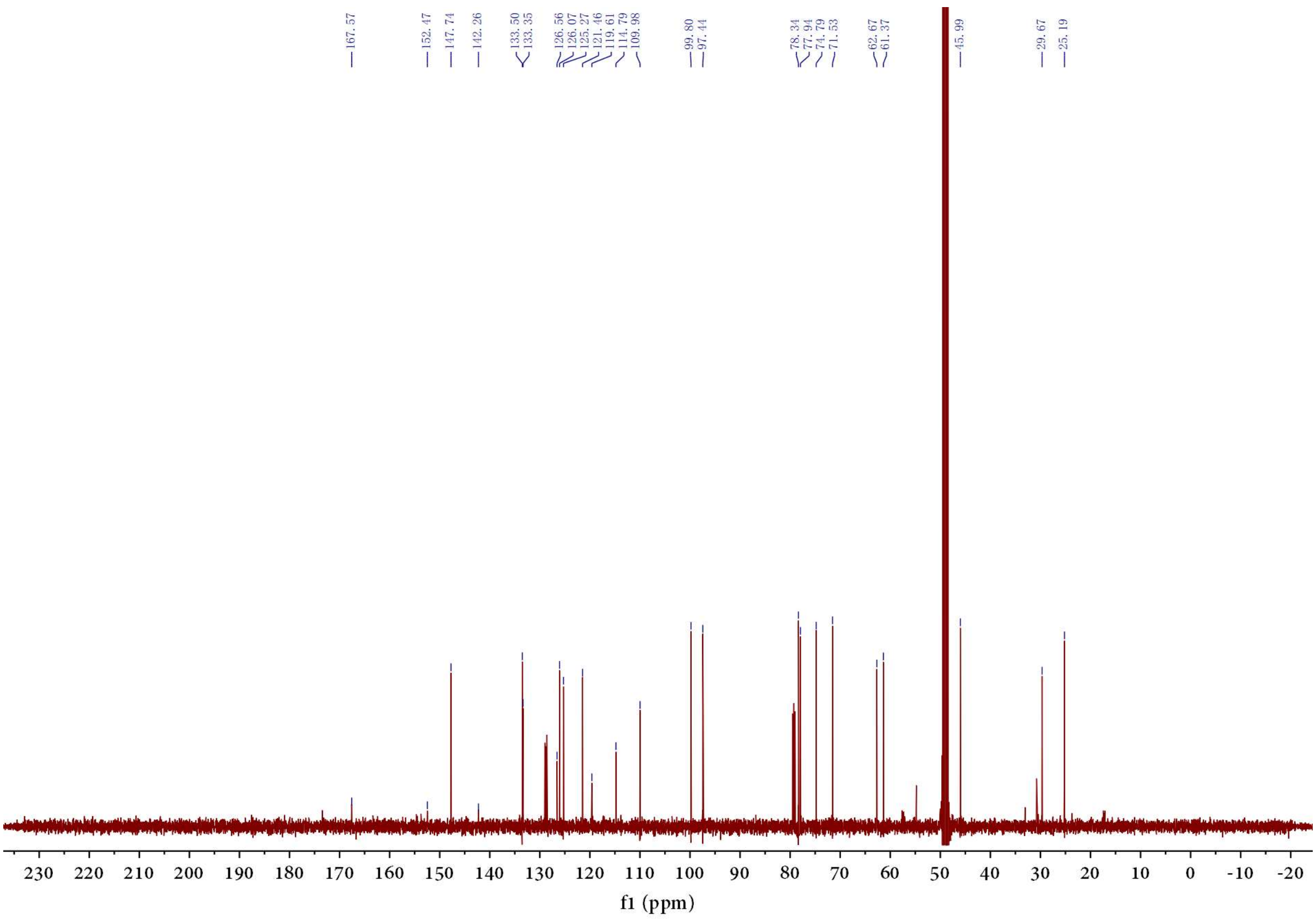

Figure S21. ^{13}C spectrum of (3*S*)-pumiloside (**6**) (MeOD-d_4, 126 MHz, 298 K).

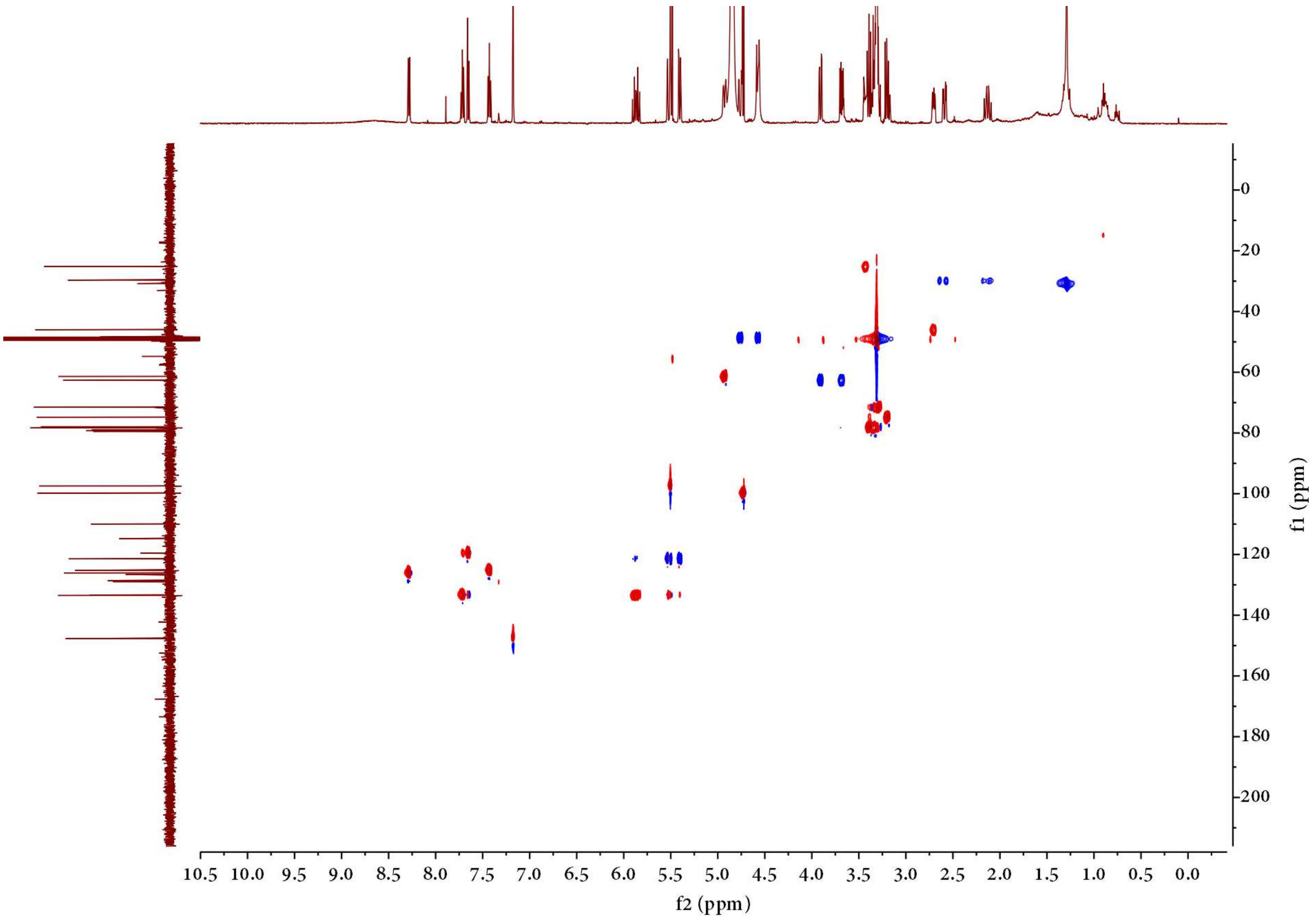

Figure S22. HSQC spectrum of (3*S*)-pumiloside (**6**) (MeOD-d_4, 500 MHz, 298 K).

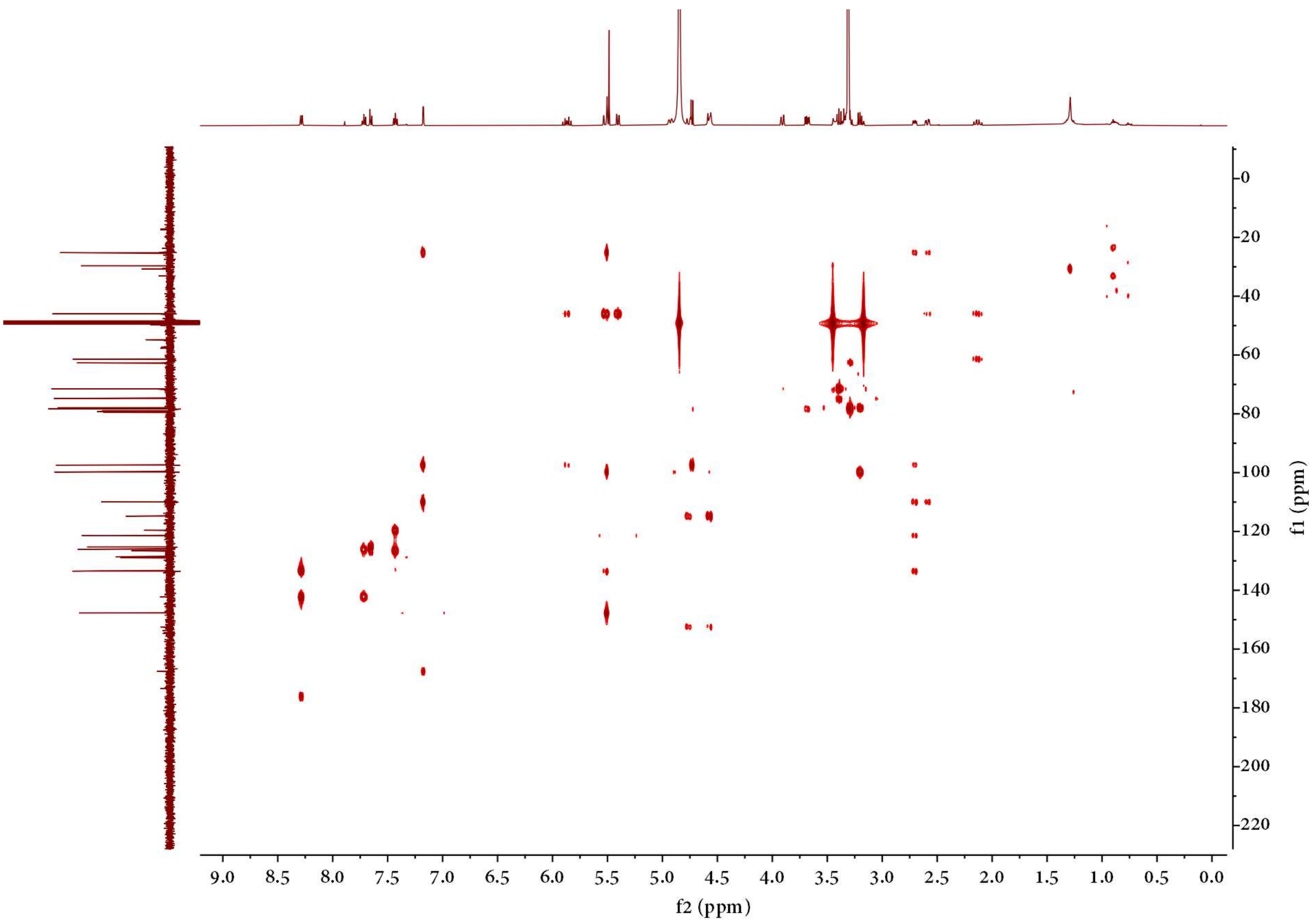

Figure S23. HMBC spectrum of (3*S*)-pumiloside (**6**) (MeOD-d_4, 500 MHz, 298 K).

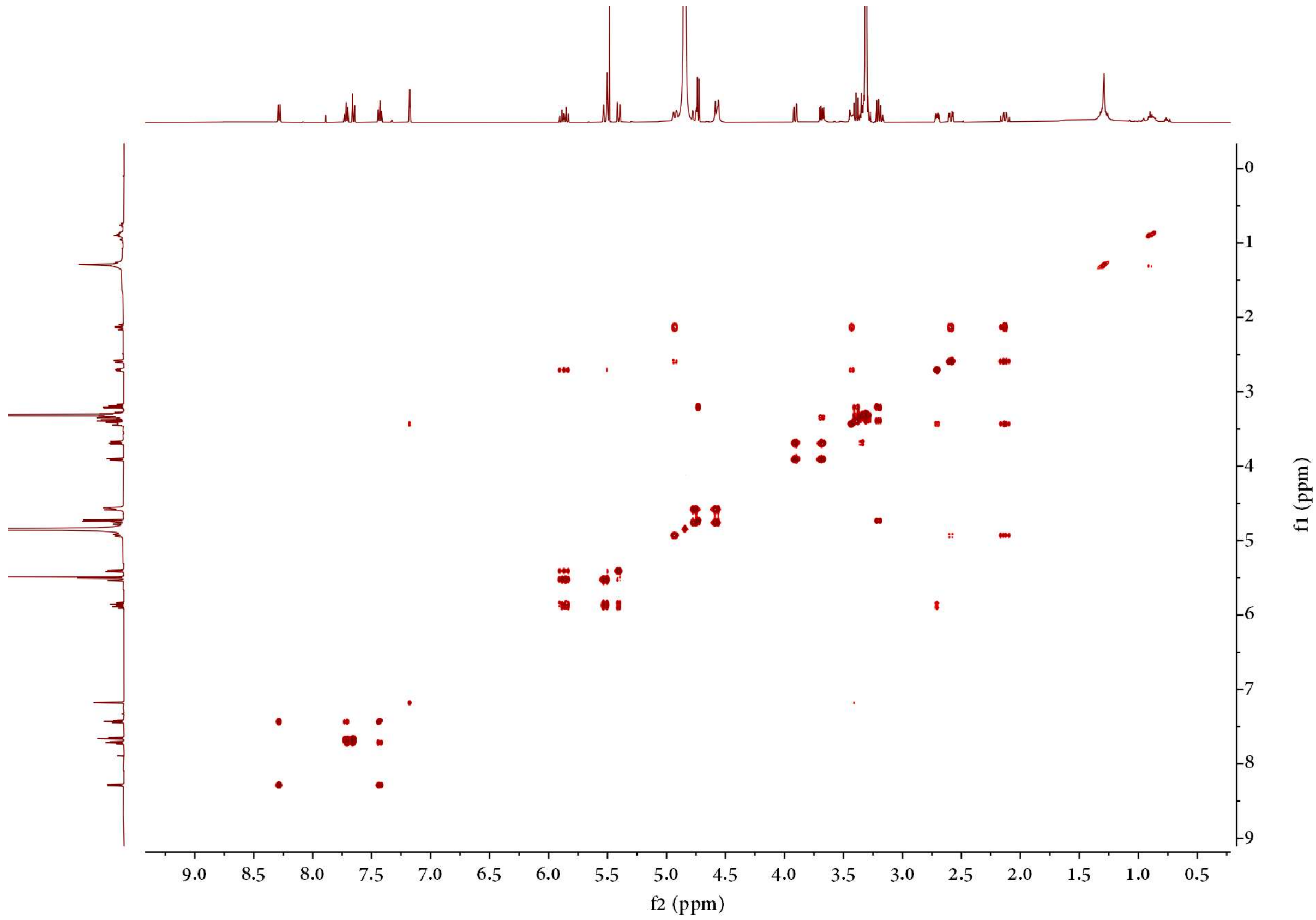

Figure S24. COSY spectrum of (3*S*)-pumiloside (**6**) (MeOD-d_4, 500 MHz, 298 K).

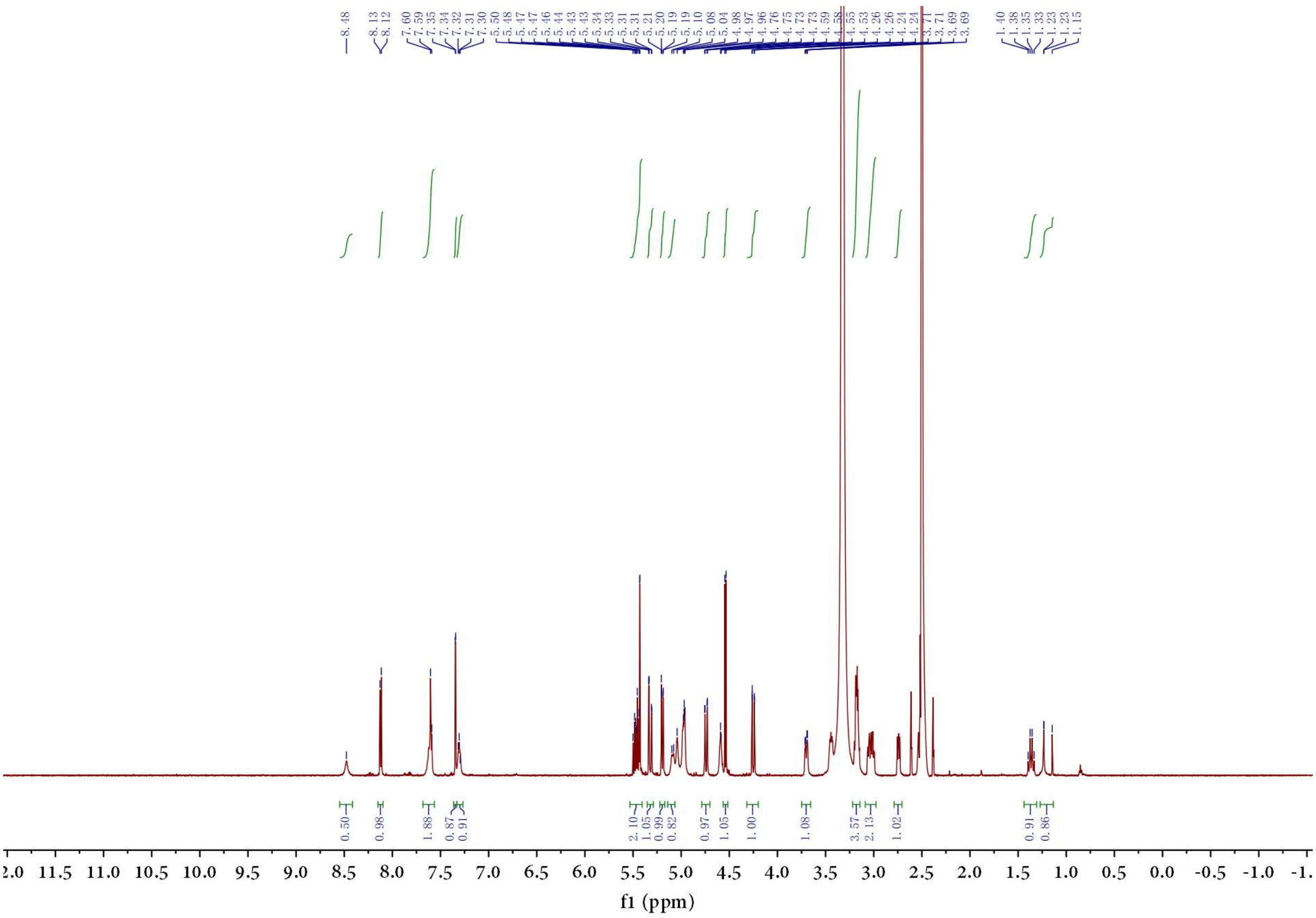

Figure S25. ^{1}H spectrum of (3*R*)-pumiloside (**15**) (DMSO-d_6, 600 MHz, 298 K).

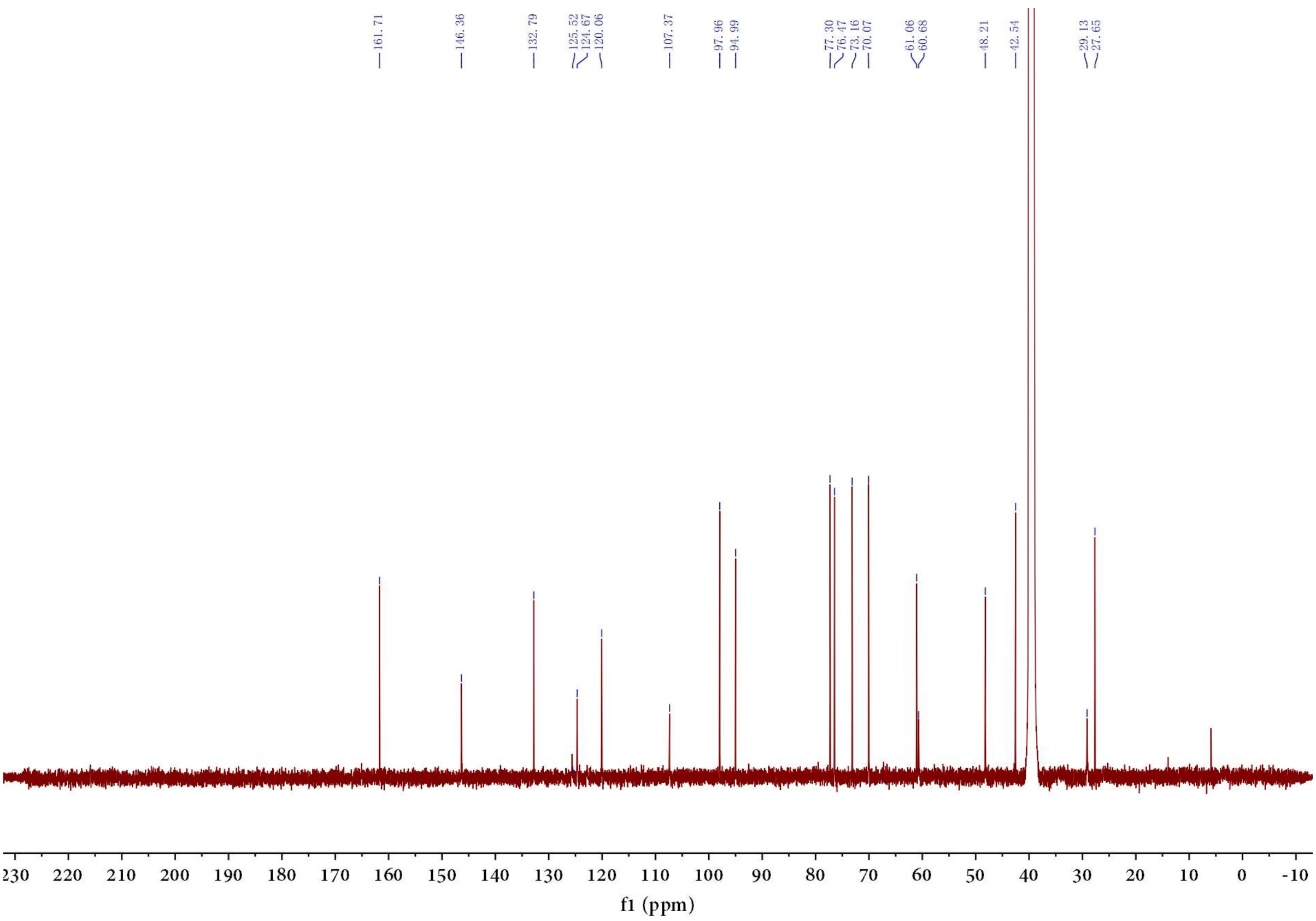

Figure S26. ^{13}C spectrum of (3*R*)-pumiloside (**15**) (DMSO-d_6, 151 MHz, 298 K).

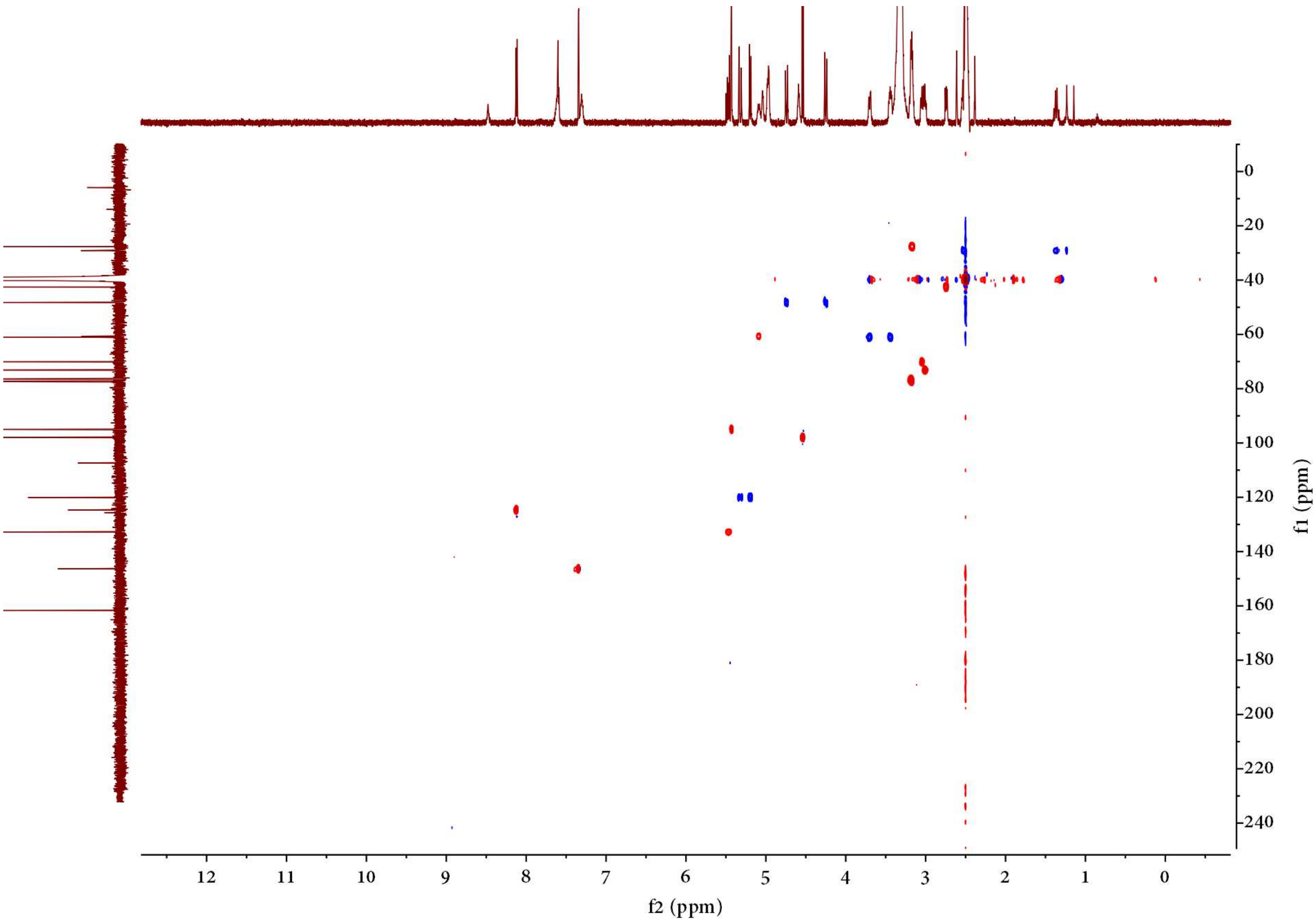

Figure S27. HSQC spectrum of (3*R*)-pumiloside (**15**) (DMSO-d_6, 600 MHz, 298 K).

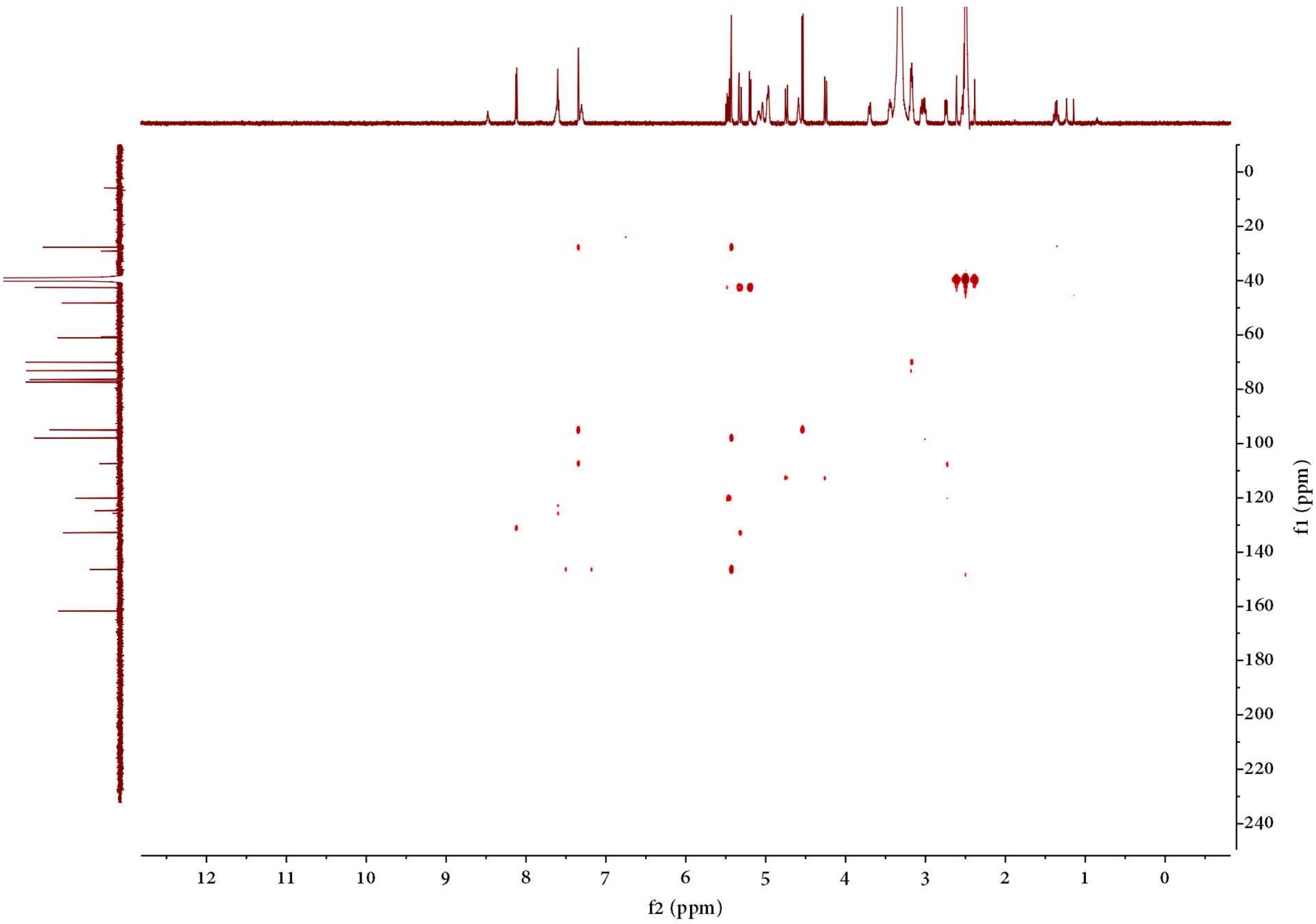

Figure S28. HMBC spectrum of (3*R*)-pumiloside (**15**) (DMSO-d_6, 600 MHz, 298 K).

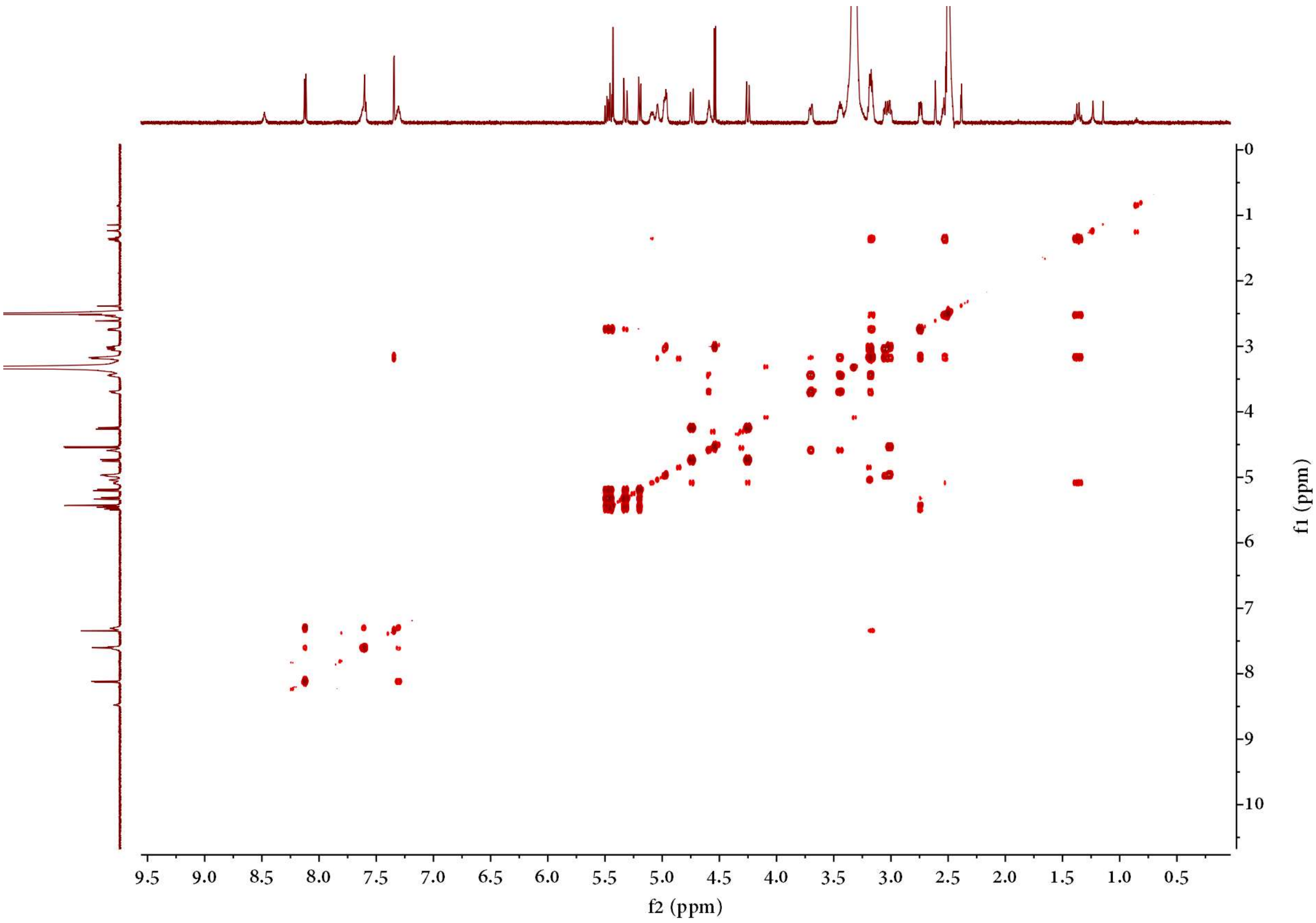

Figure S29. COSY spectrum of (3*R*)-pumiloside (**15**) (DMSO-d_6, 600 MHz, 298 K).

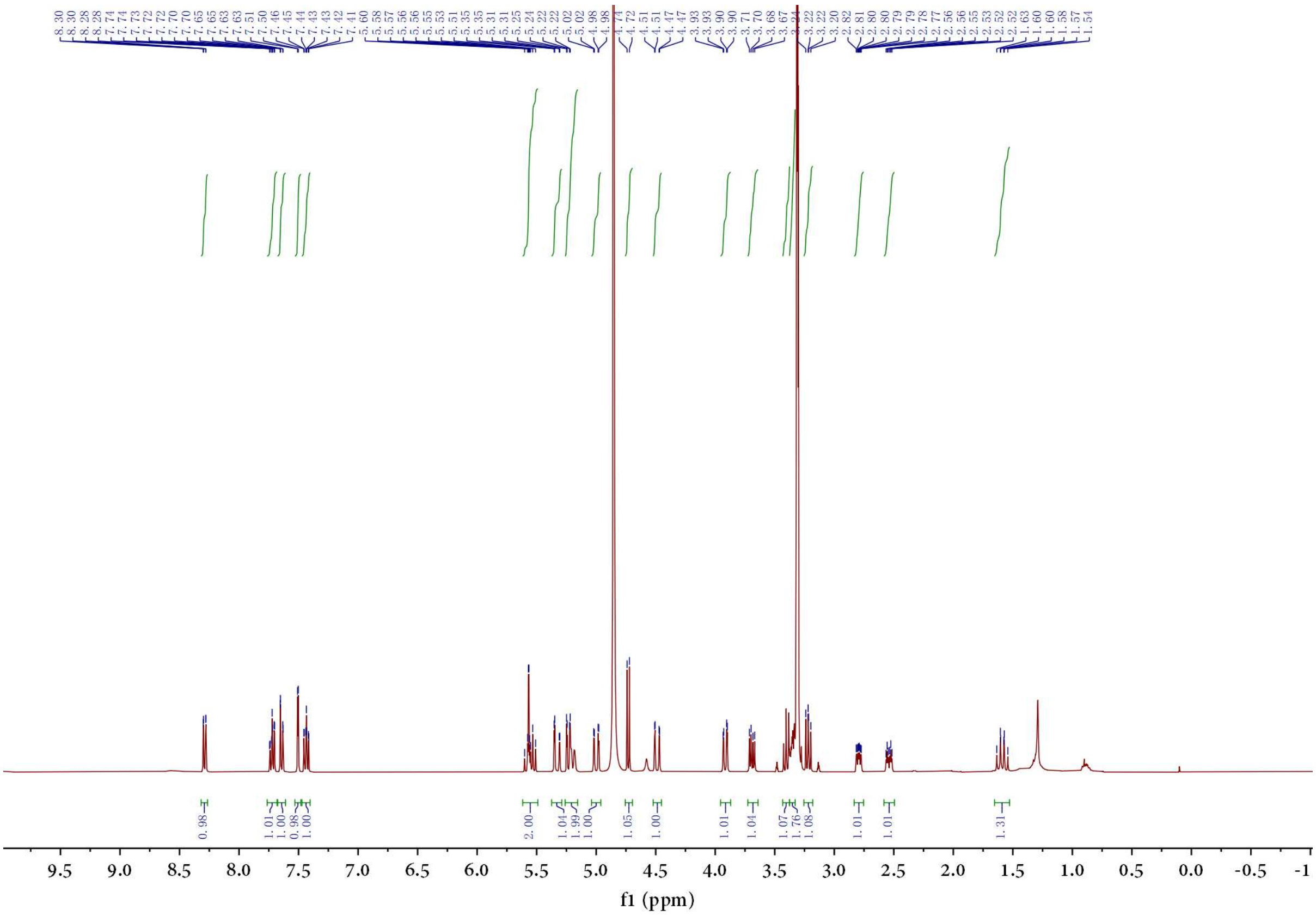

Figure S30. ^{1}H spectrum of (3*R*)-pumiloside (**15**) (MeOD-d$_4$, 400 MHz, 298 K).

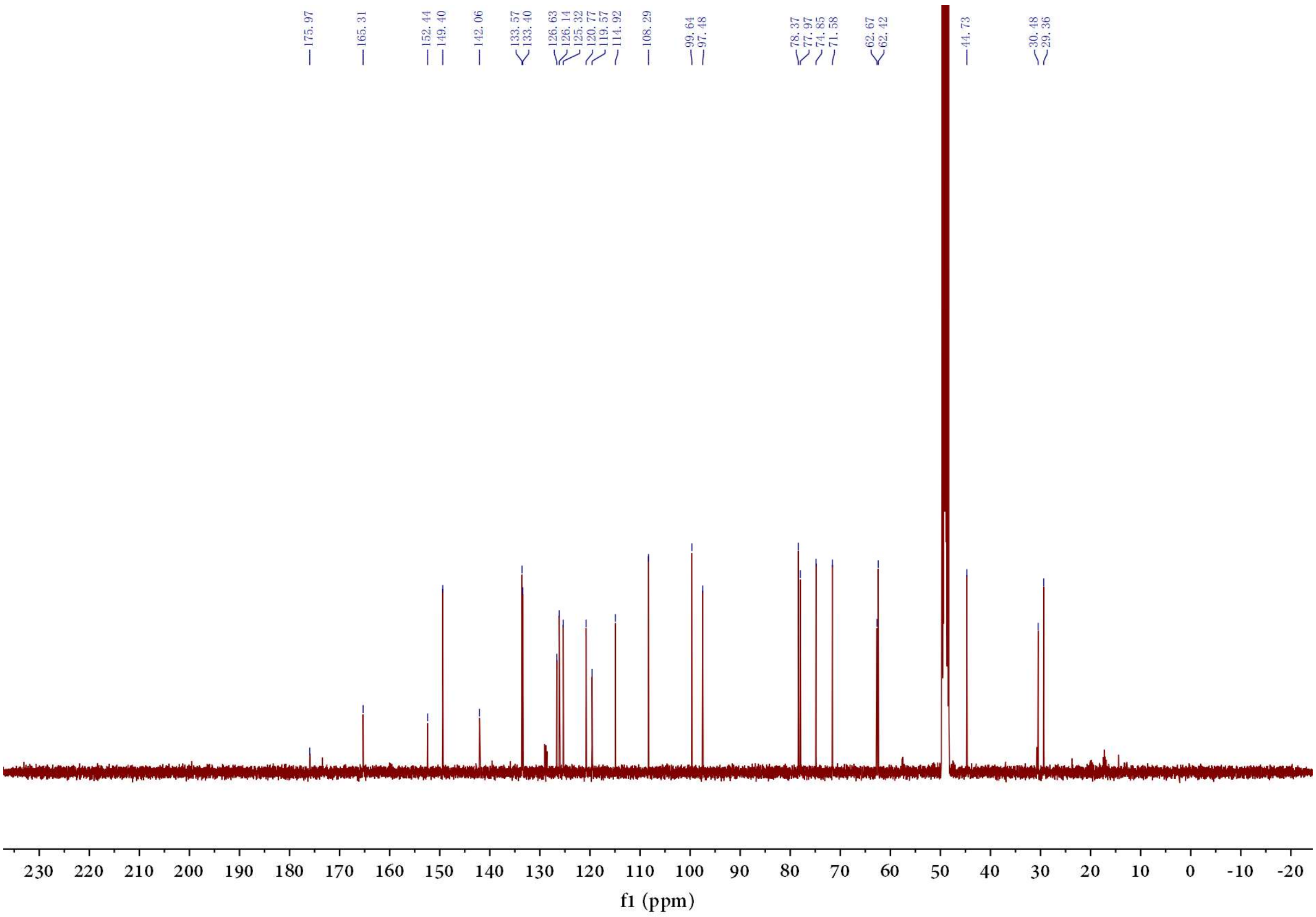

Figure S31. ^{13}C spectrum of (3*R*)-pumiloside (**15**) (MeOD-d$_4$, 101 MHz, 298 K).

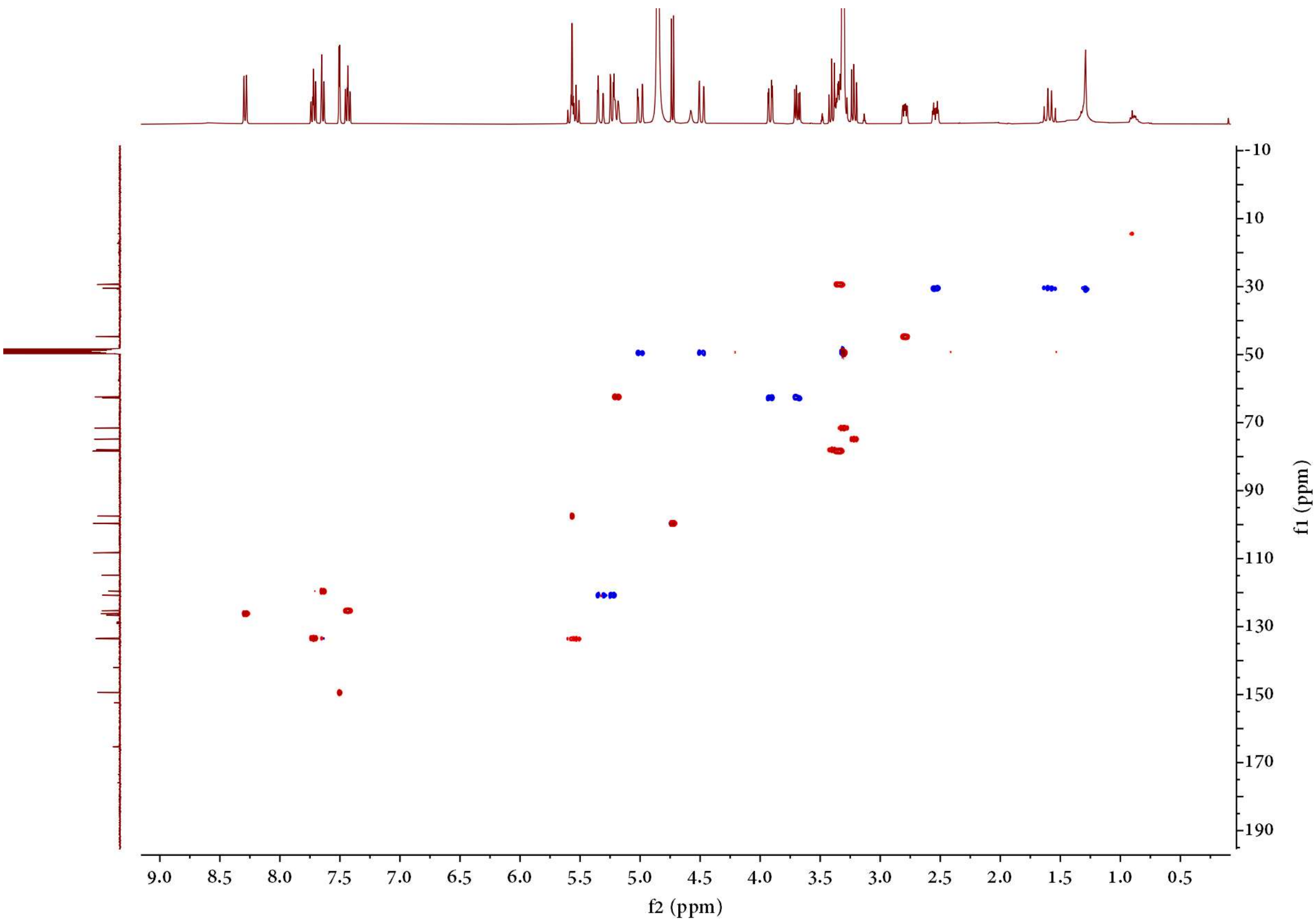

Figure S32. HSQC spectrum of (3*R*)-pumiloside (**15**) (MeOD-d$_4$, 400 MHz, 298 K).

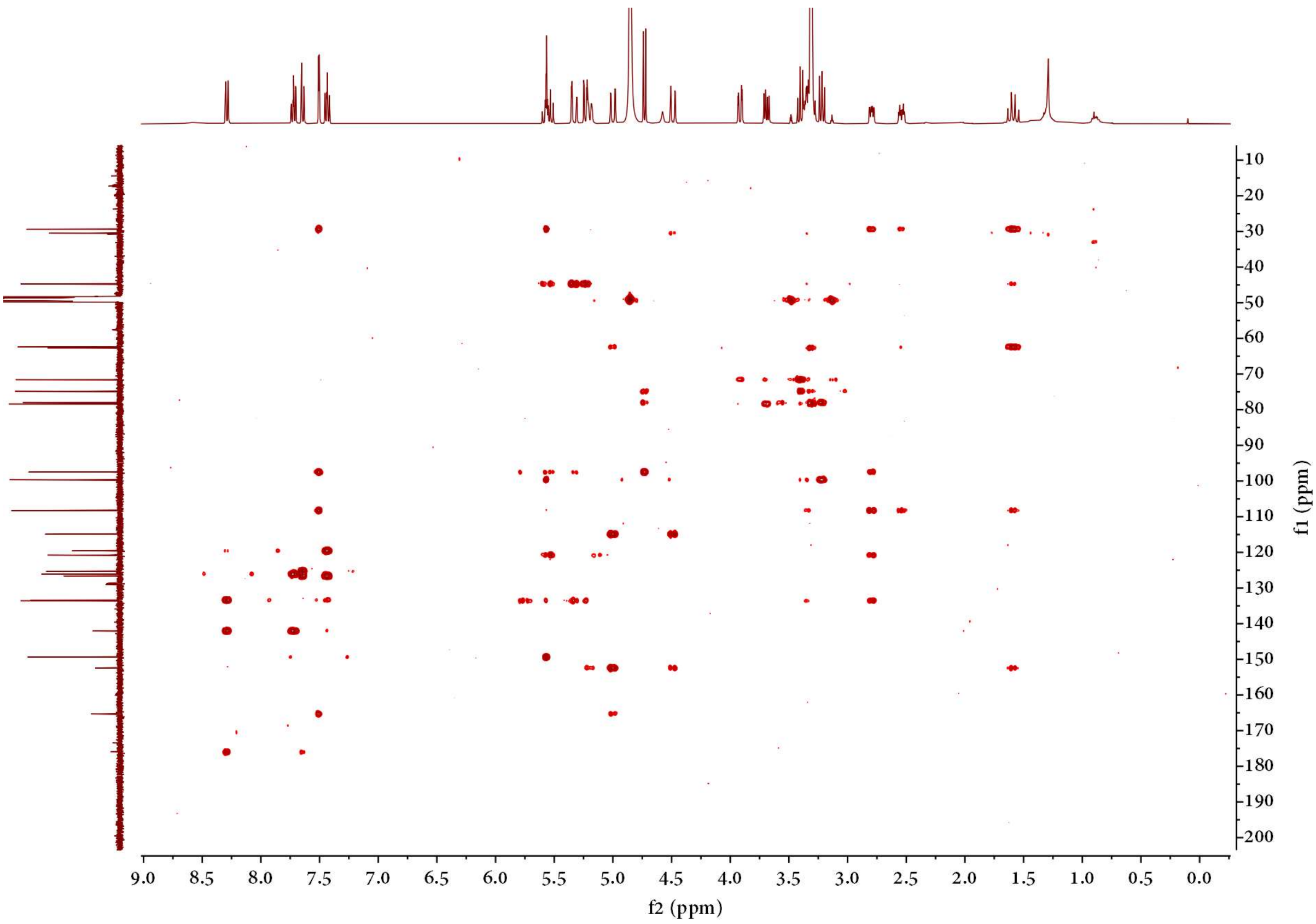

Figure S33. HMBC spectrum of (3*R*)-pumiloside (**15**) (MeOD-d$_4$, 400 MHz, 298 K).

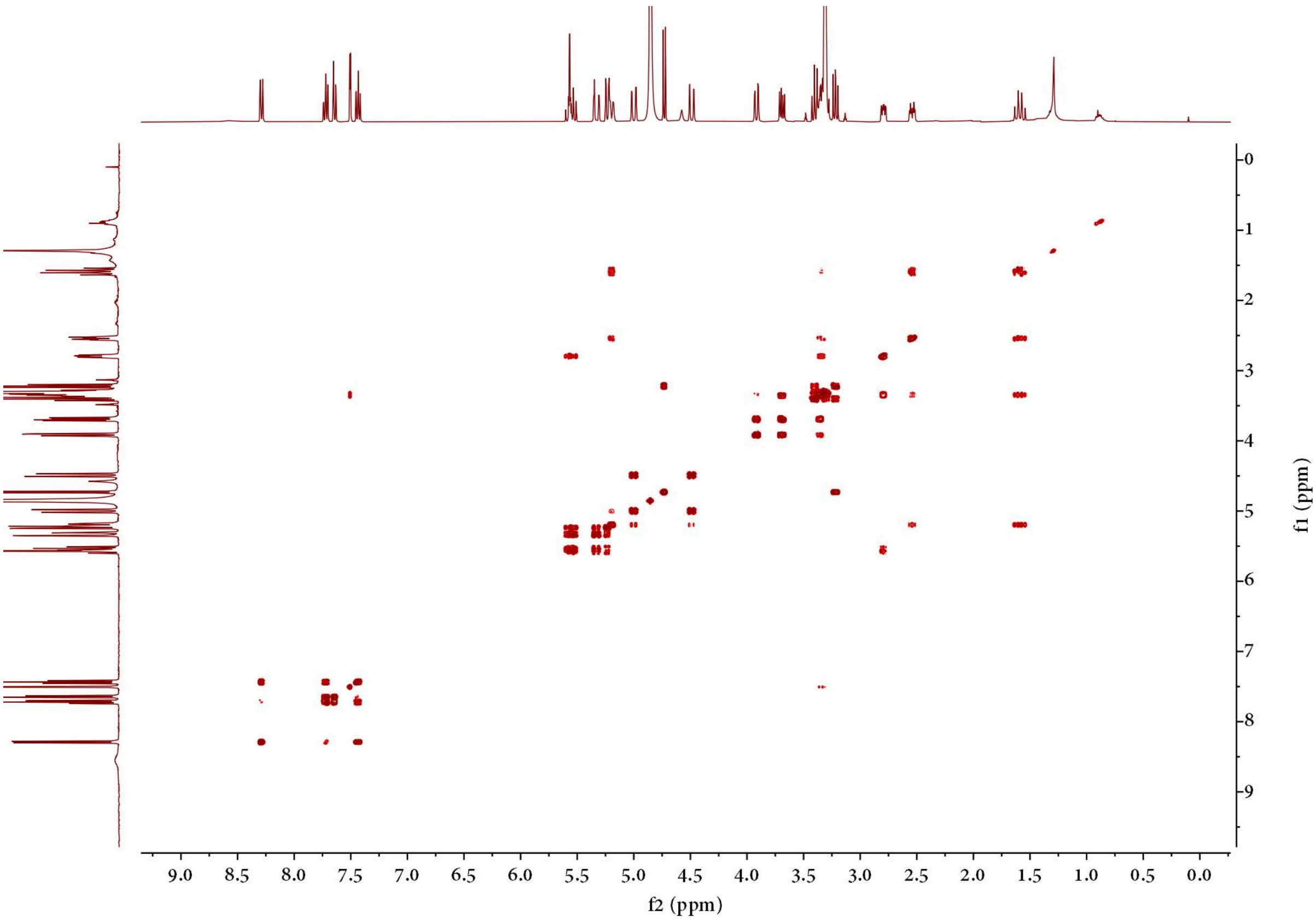

Figure S34. COSY spectrum of (3*R*)-pumiloside (**15**) (MeOD-d_4, 400 MHz, 298 K).

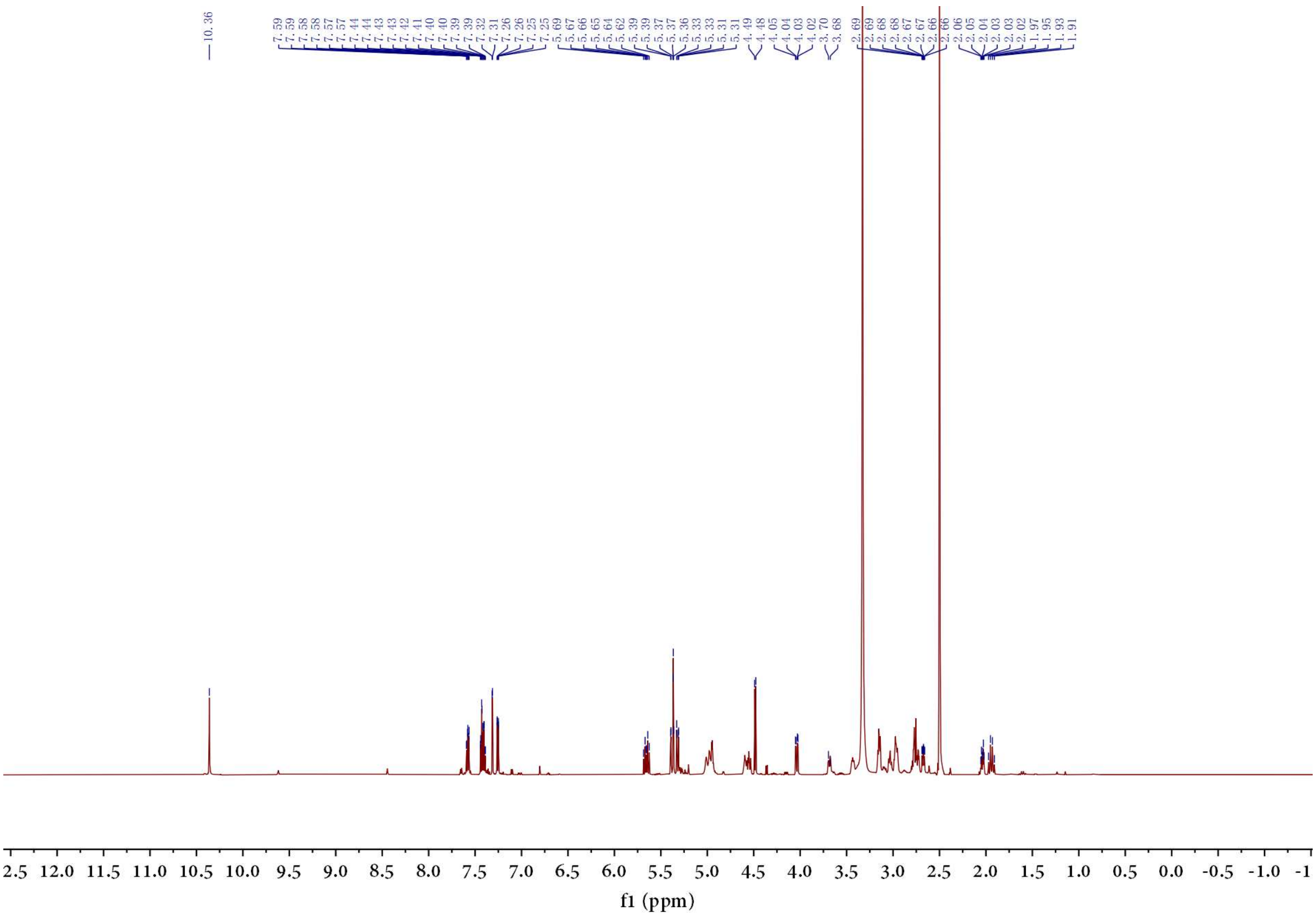

Figure S35. ^{1}H spectrum of vincosamide ketolactam (**13**) (DMSO-d_6, 600 MHz, 298 K).

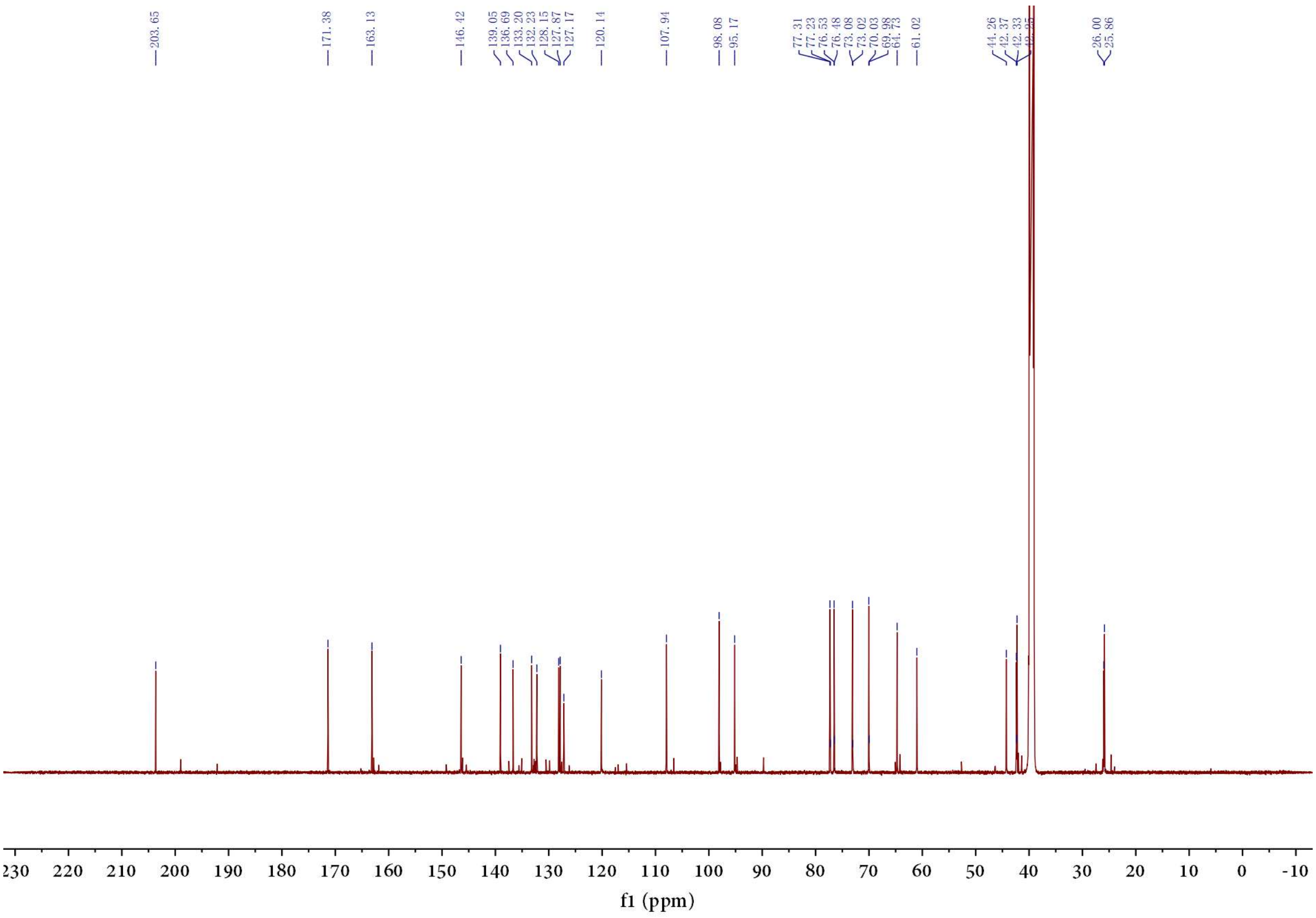

Figure S36. ^{13}C spectrum of vincosamide ketolactam (**13**) (DMSO-d_6, 151 MHz, 298 K).

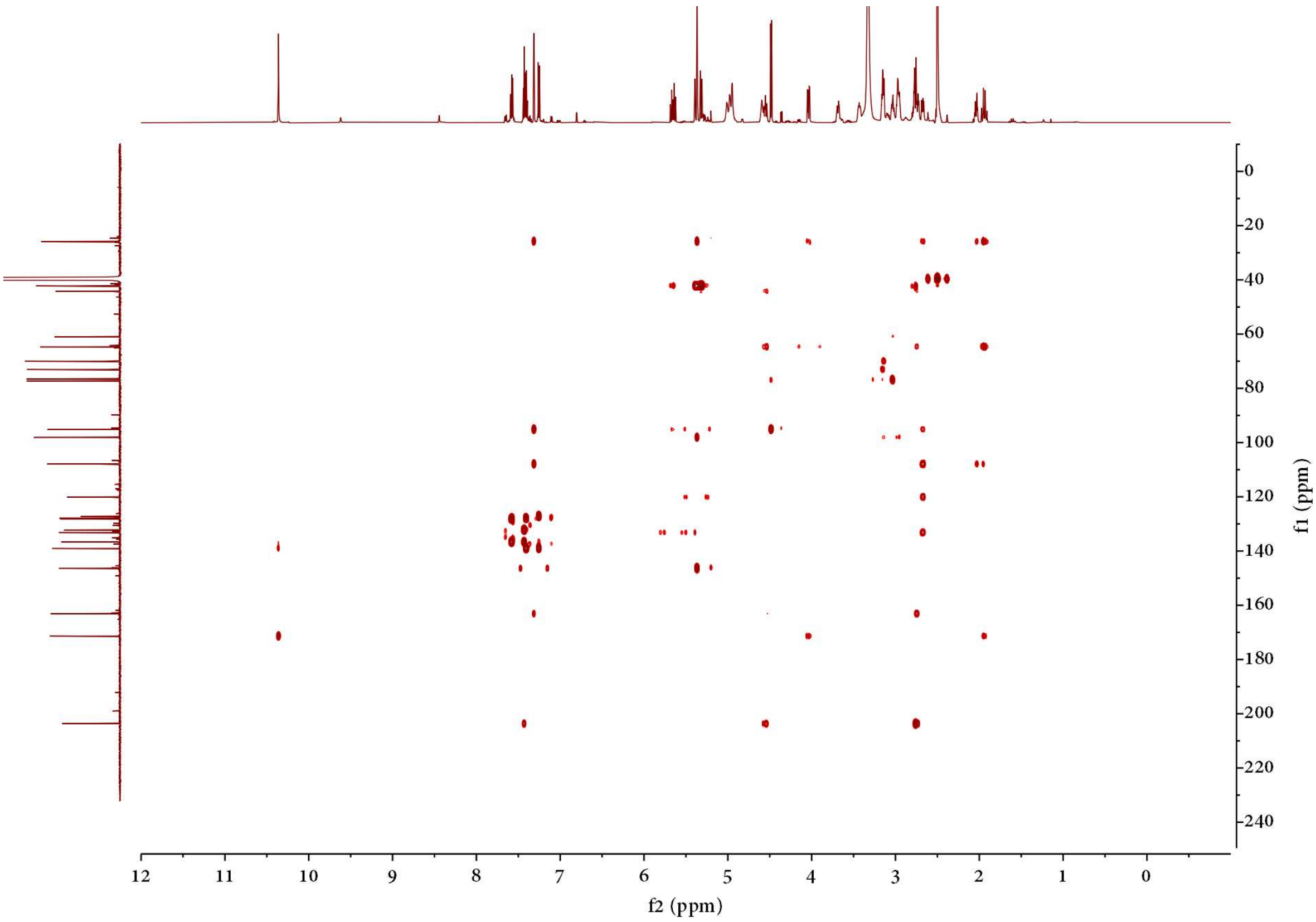

Figure S37. HSQC spectrum of vincosamide ketolactam (**13**) (DMSO-d$_6$, 600 MHz, 298 K).

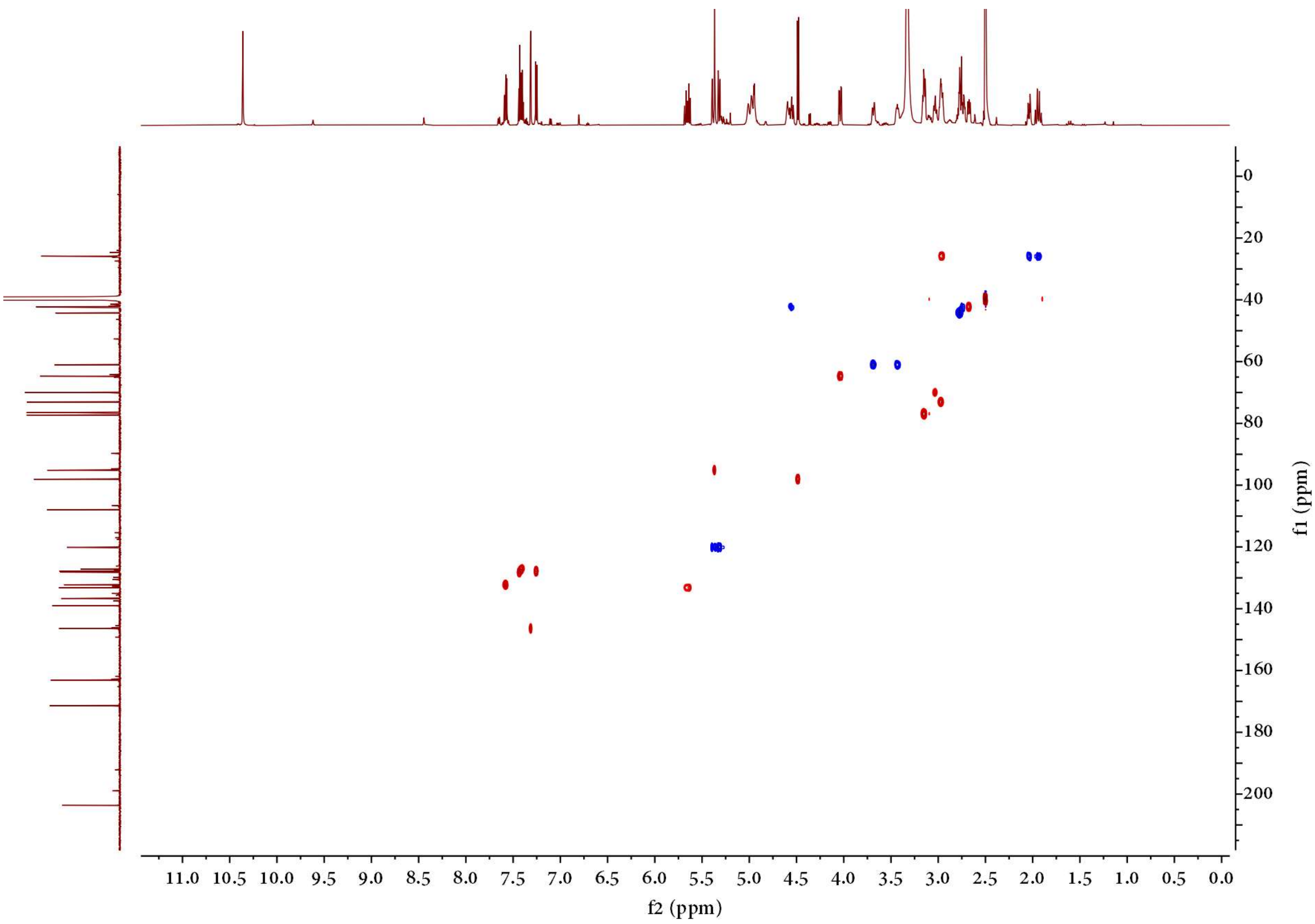

Figure S38. HMBC spectrum of vincosamide ketolactam (**13**) (DMSO-d$_6$, 600 MHz, 298 K).

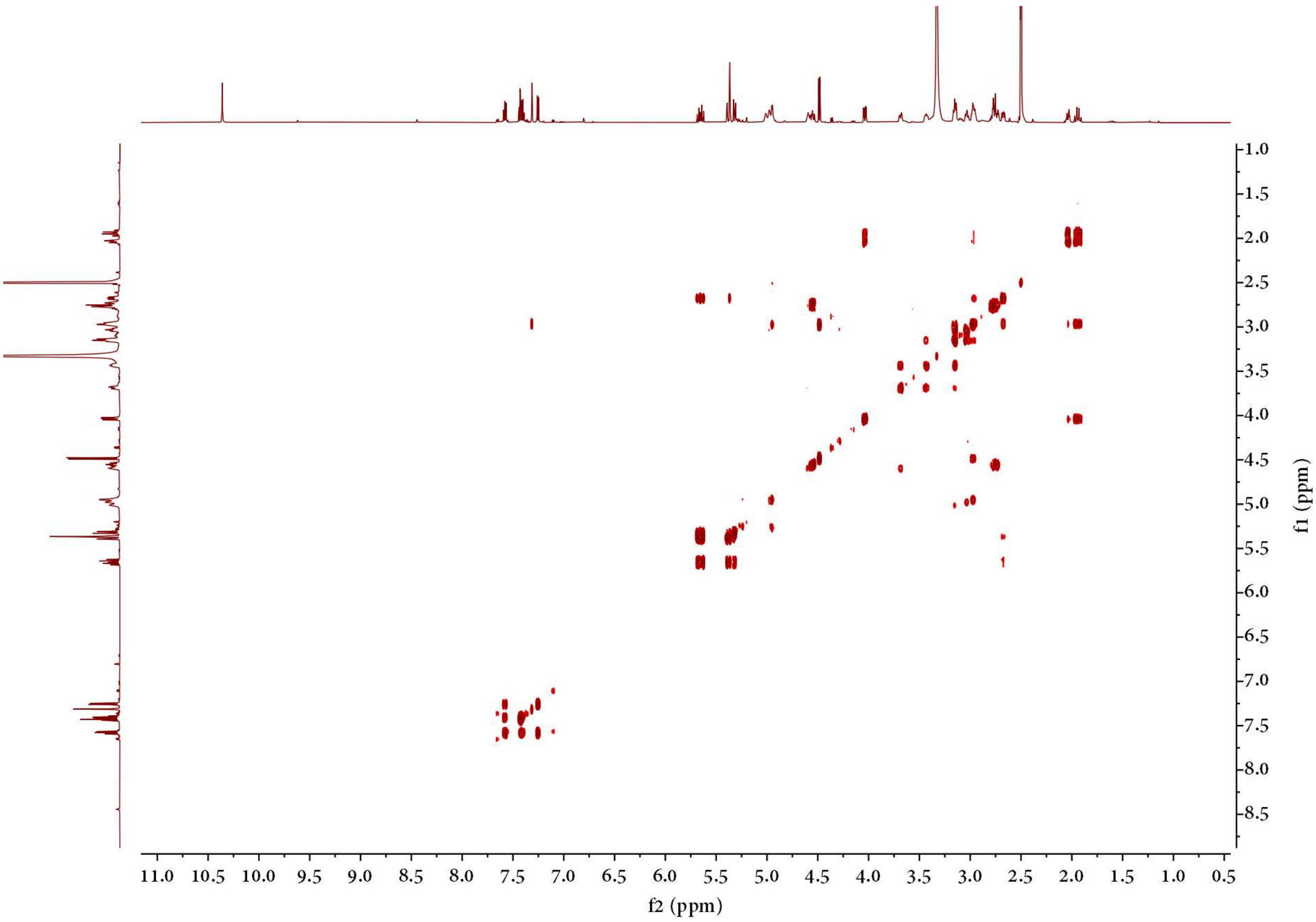

Figure S39. COSY spectrum of vincosamide ketolactam (**13**) (DMSO-d_6, 600 MHz, 298 K).

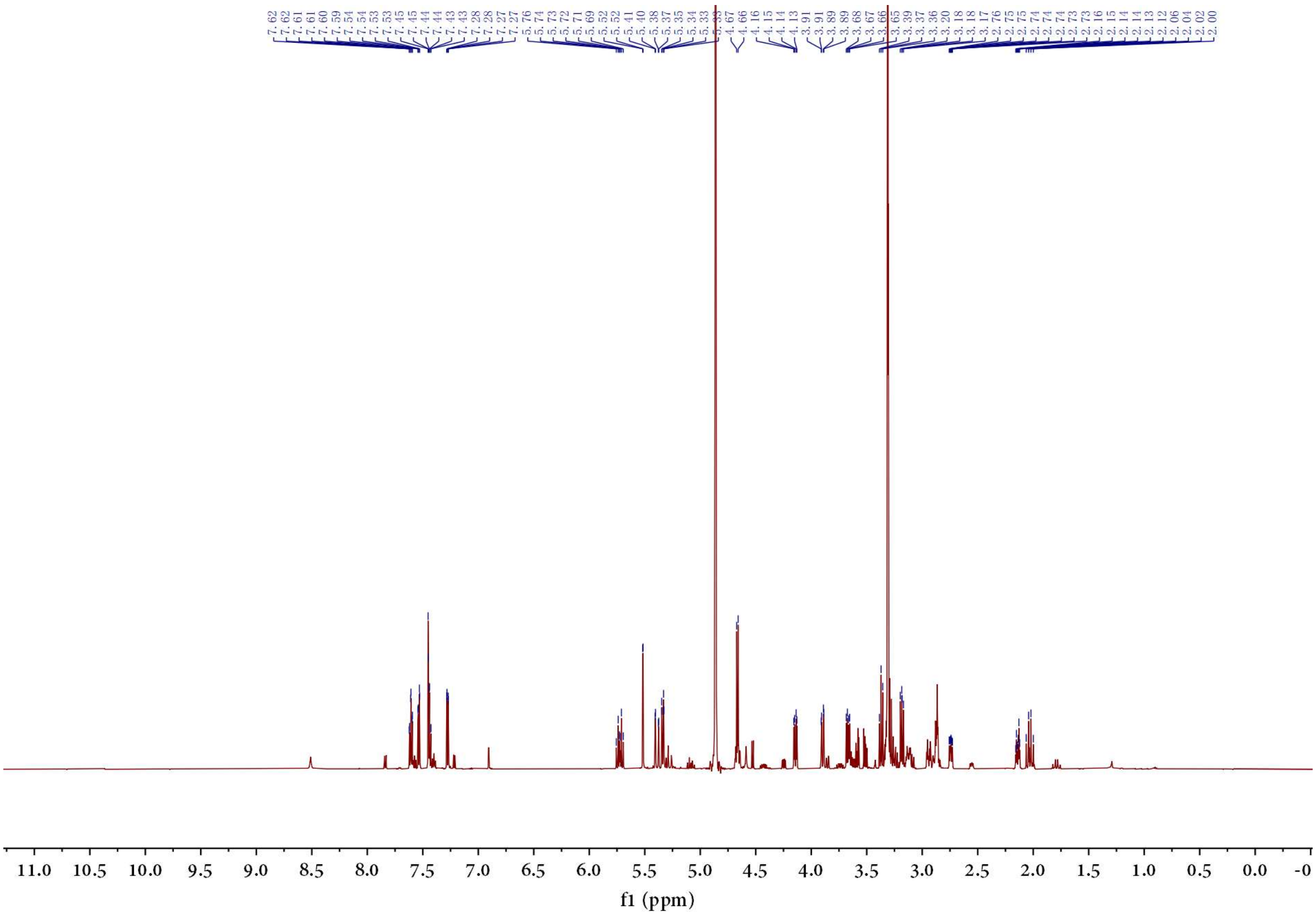

Figure S40. ^{1}H spectrum of vincosamide ketolactam (**13**) (MeOD-d_4, 600 MHz, 298 K).

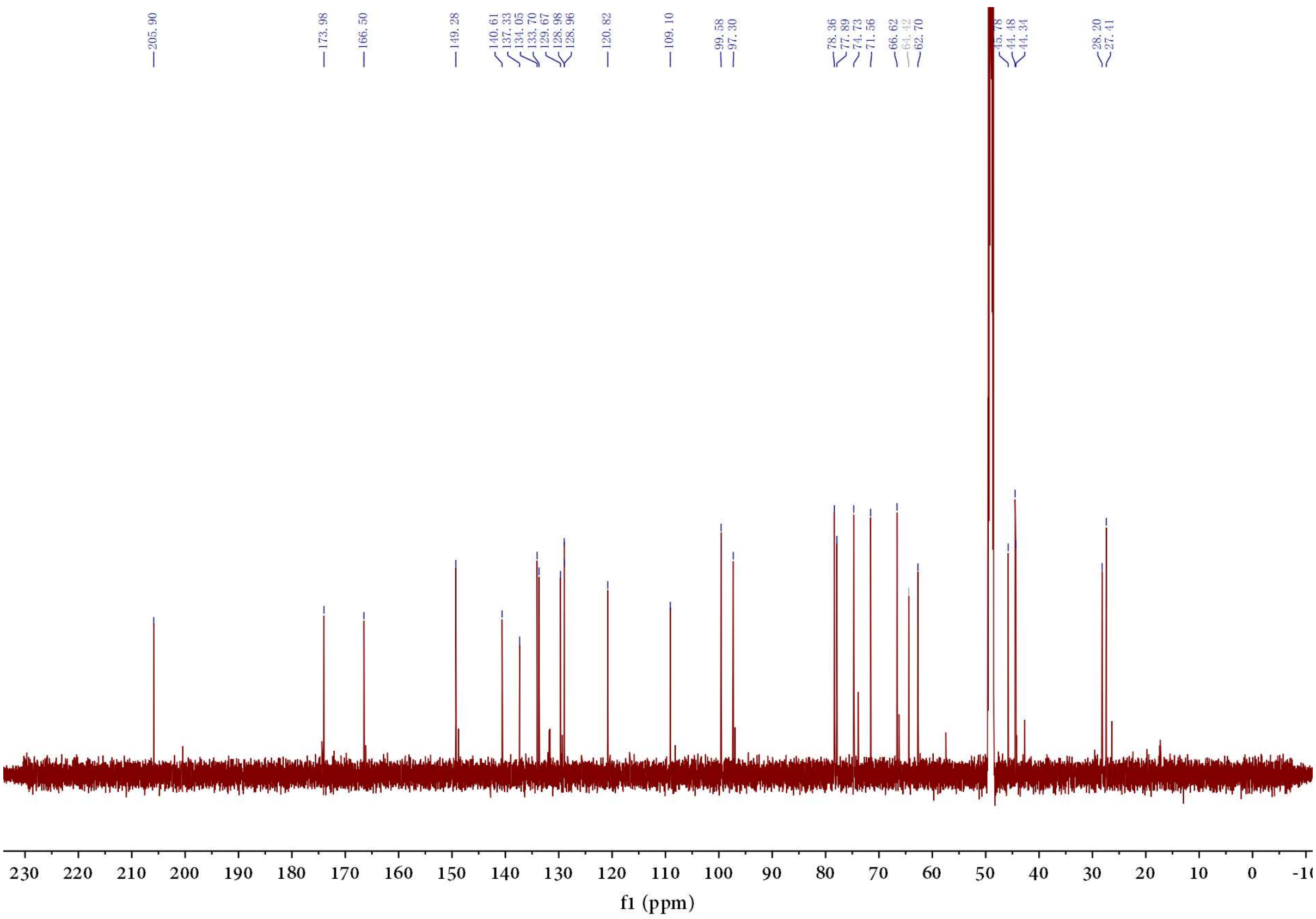

Figure S41. ^{13}C spectrum of vincosamide ketolactam (**13**) (MeOD-d_4, 151 MHz, 298 K).

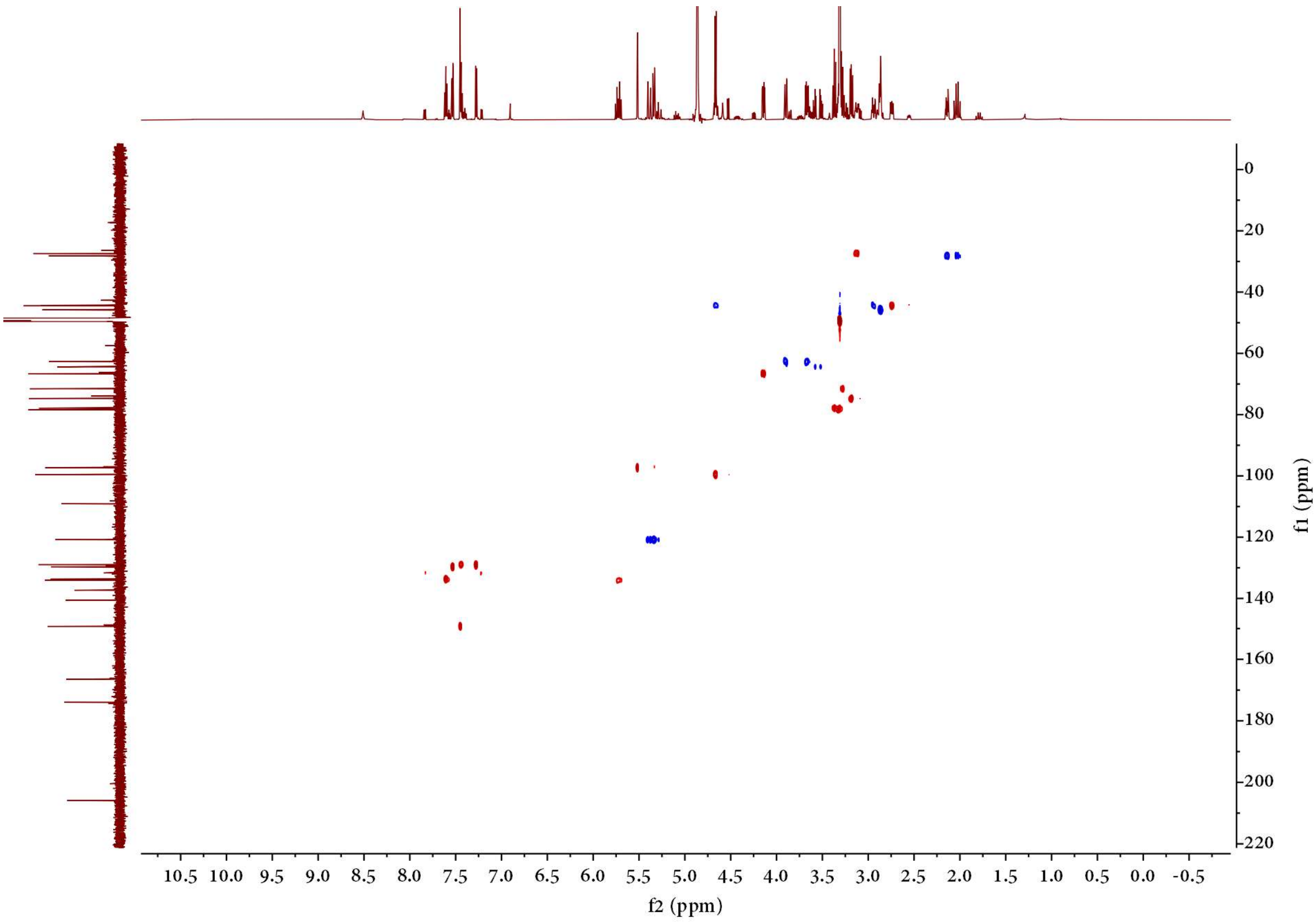

Figure S42. HSQC spectrum of vincosamide ketolactam (**13**) (MeOD-d_4, 600 MHz, 298 K).

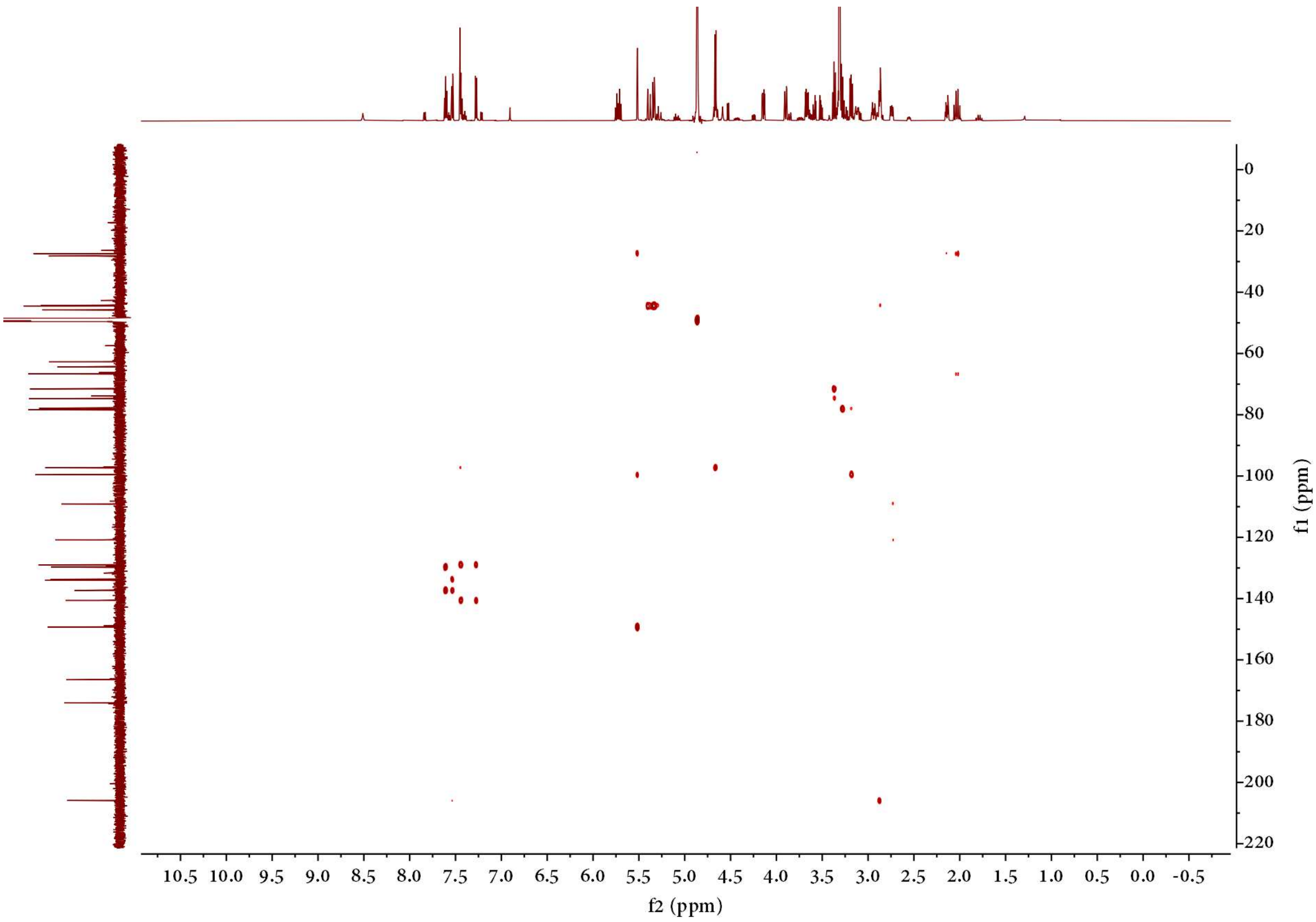

Figure S43. HMBC spectrum of vincosamide ketolactam (**13**) (MeOD-d_4, 600 MHz, 298 K).

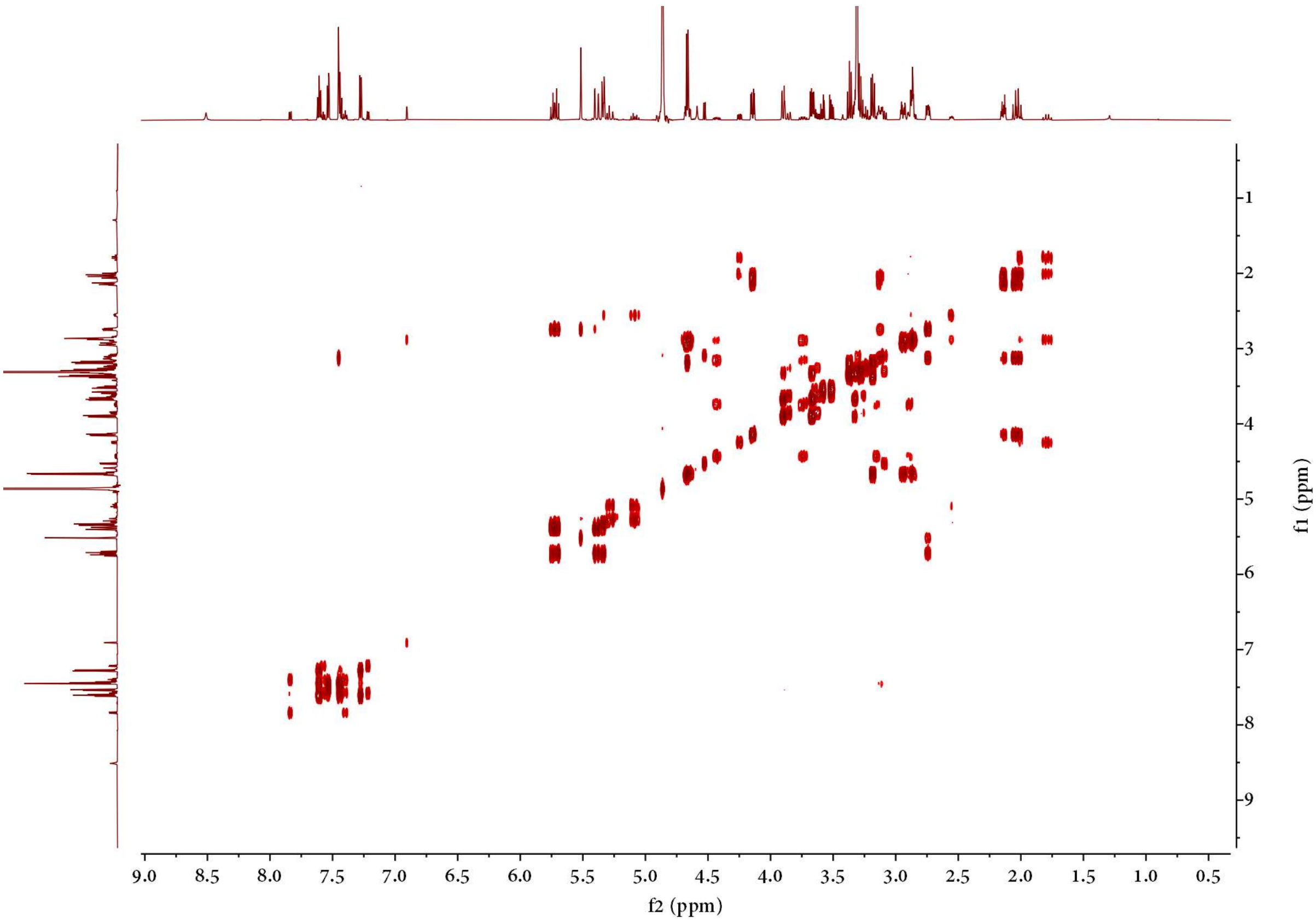

Figure S44. COSY spectrum of vincosamide ketolactam (**13**) (MeOD-d_4, 600 MHz, 298 K).

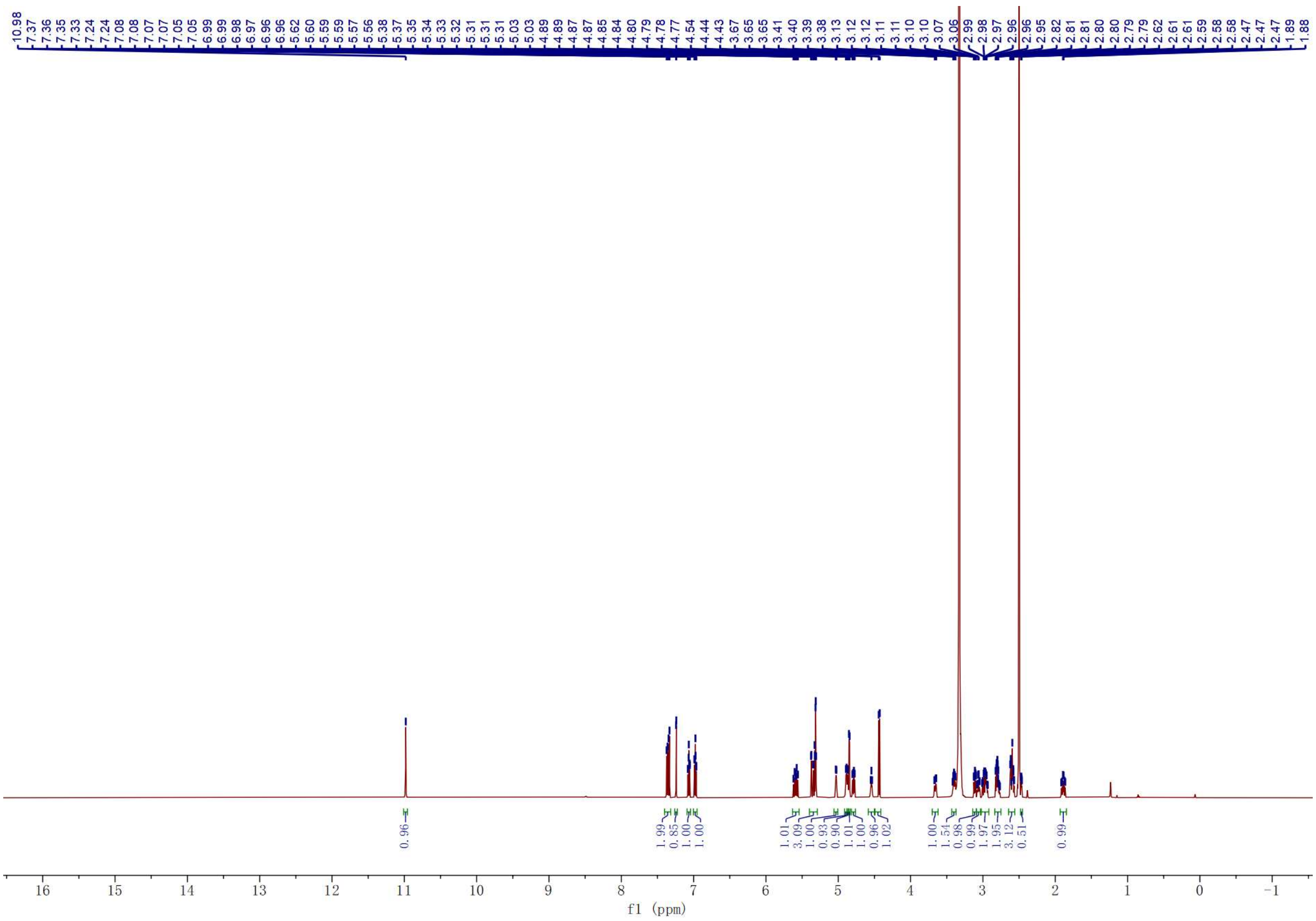

Figure S45. ^{1}H spectrum of strictosamide (**5**) (DMSO-d_6, 600 MHz, 298 K).

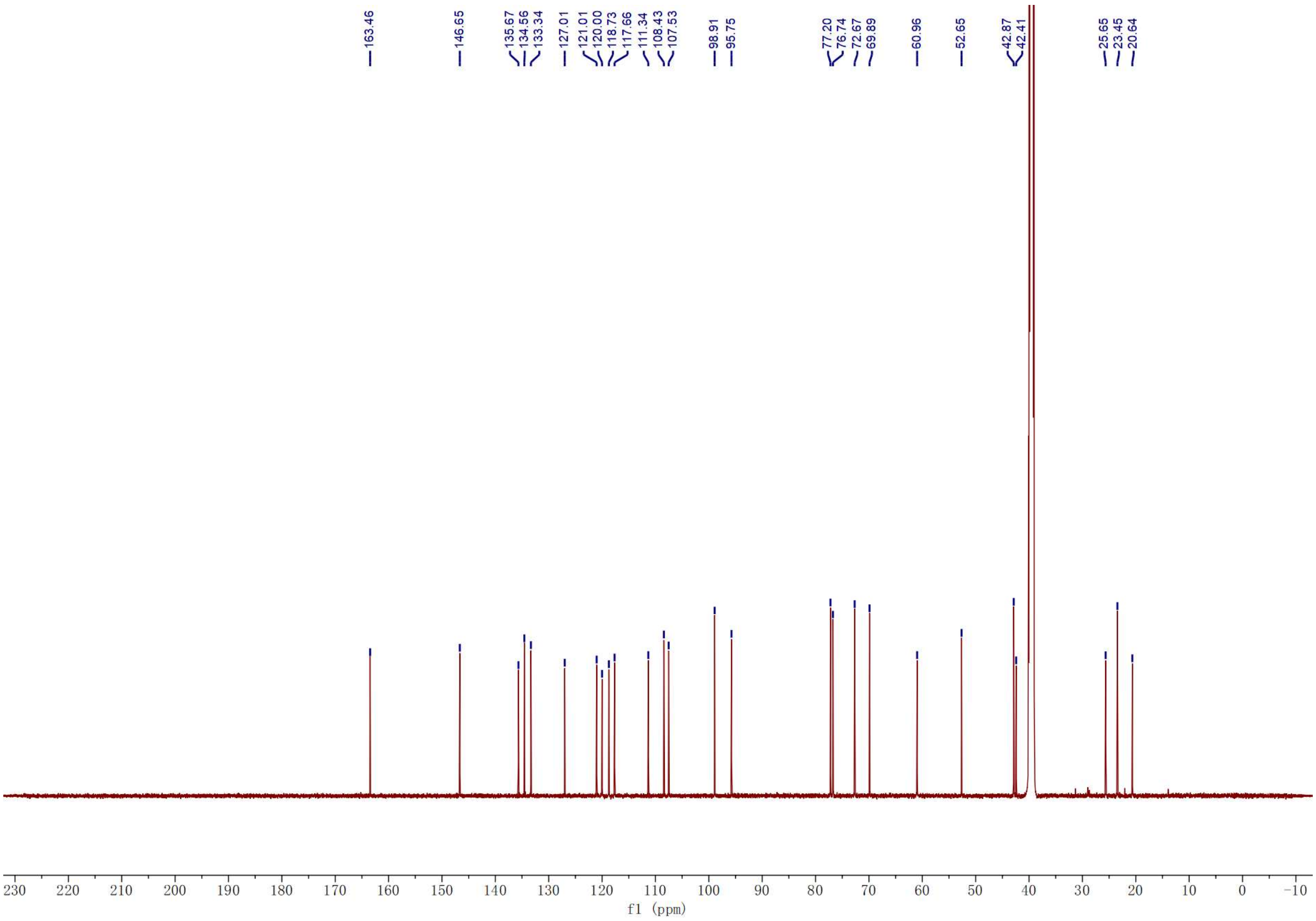

Figure S46. ^{13}C spectrum of strictosamide (**5**) (DMSO-d_6, 151 MHz, 298 K).

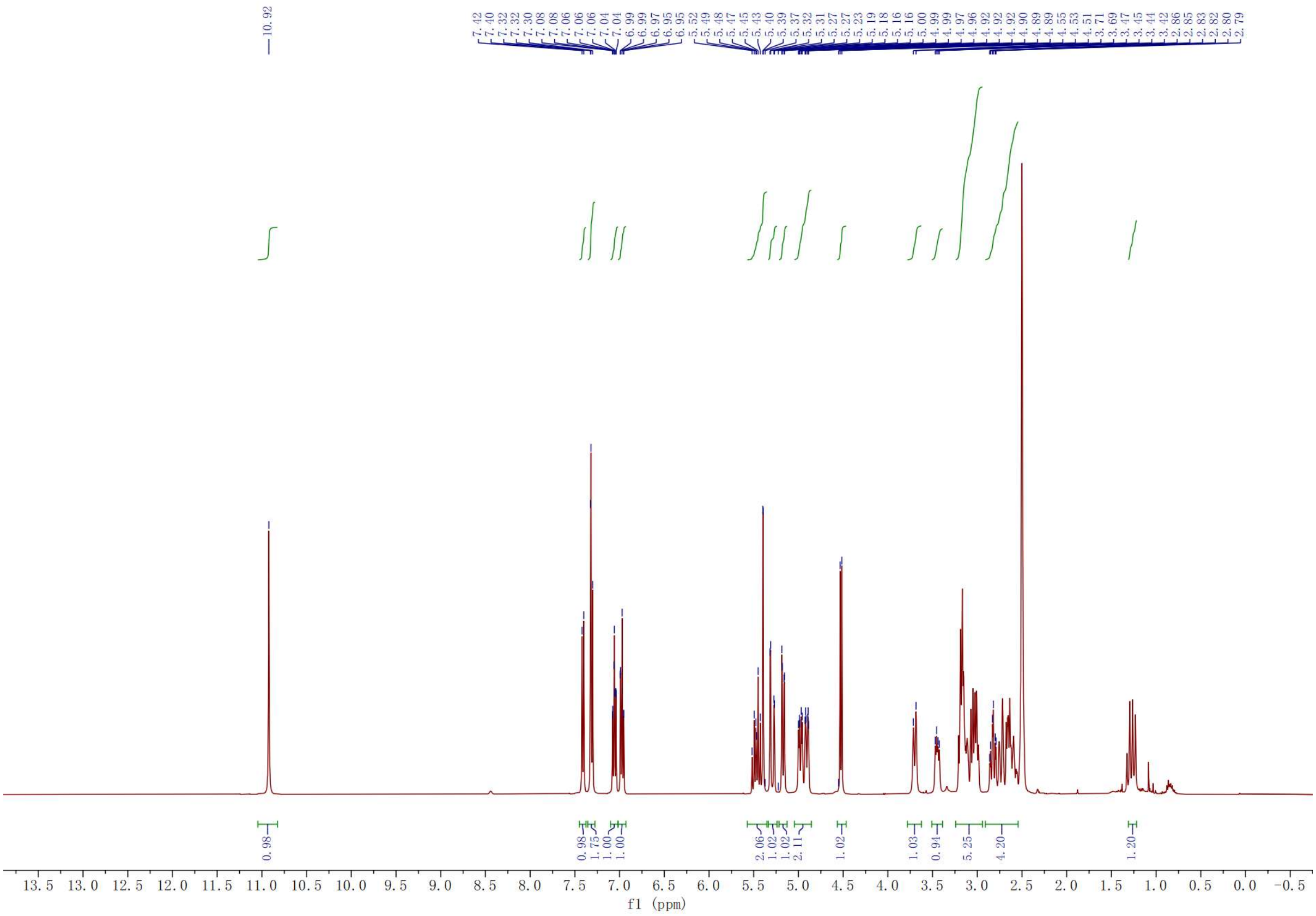

Figure S47. ^{1}H spectrum of vincosamide (**12**) (DMSO-d_6, 400 MHz, 298 K).

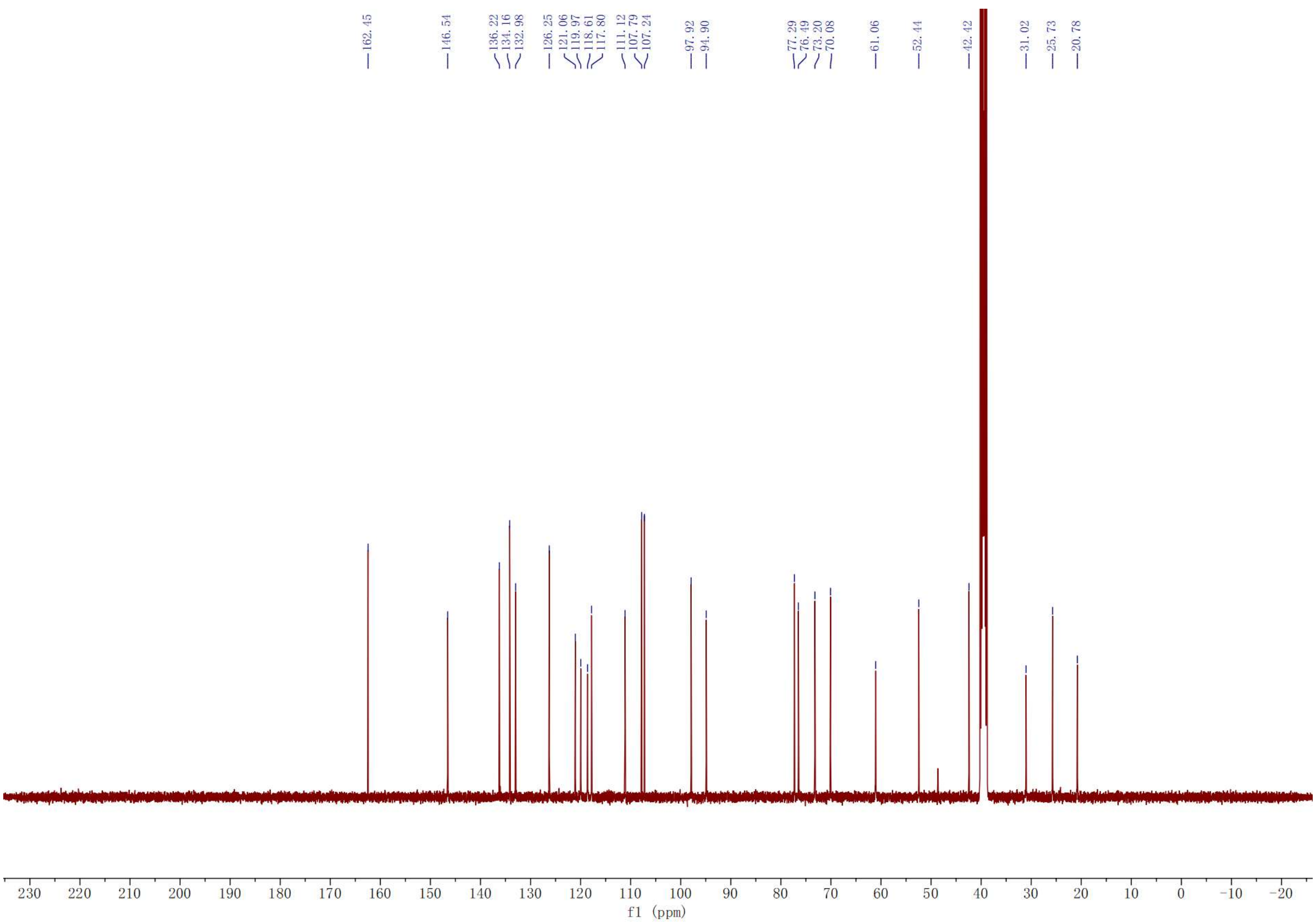

Figure S48. ^{13}C spectrum of vincosamide (**12**) (DMSO-d_6, 101 MHz, 298 K).

Supporting Tables

Table S1. ^{1}H and ^{13}C NMR assignments of (3*S*)-pumiloside (**6**) (DMSO-d$_6$, 500 MHz, 298 K).

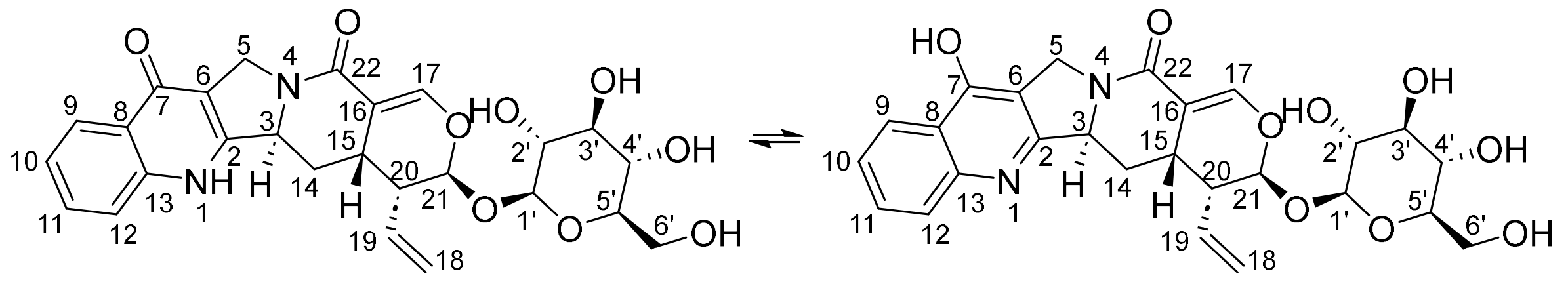

(3*S*)-Pumiloside (**6**)

Atom	^{13}C ppm	^{1}H ppm (m, Hz)	^{13}C ppm	^{1}H ppm (m, Hz)
	DMSO-d$_6$		MeOD-d$_4$	
1	N*H*	8.42 (s, 1H)	N*H*	-
2	149.6[a]	-	152.47	-
3	59.54	4.76 (d, *J* = 11.7 Hz, 1H)	61.37	4.93 (d, *J* = 12.5 Hz, 1H)
4	-	-	-	-
5	47.50	4.49 (dd, *J* = 14.2, 2.6 Hz, 1H)<br>4.33 (dd, *J* = 14.2, 1.3 Hz, 1H)	48.73[b]	4.76 (dd, *J* = 14.6, 2.4 Hz, 1H)<br>4.57 (d, *J* = 13.8 Hz, 1H)
6	112.79	-	114.79	-
7	172.5[a]	-	176.11[a]	-
8	125.40	-	126.56	-
9	124.68	8.13 (dd, *J* = 8.1, 1.1 Hz, 1H)	126.07	8.28 (dd, *J* = 8.3, 1.4 Hz, 1H)
10	123.07	7.33 (dd, *J* = 7.3, 7.3 Hz, 1H)	125.27	7.43 (ddd, *J* = 8.1, 6.9, 1.2 Hz, 1H)
11	131.39	7.65 (dd, *J* = 7.5, 7.5 Hz, 1H)	133.35	7.72 (ddd, *J* = 8.4, 6.9, 1.4 Hz, 1H)
12	118.5[a]	7.61 (d, *J* = 7.9 Hz, 1H)	119.61	7.65 (dd, *J* = 8.5, 1.1 Hz, 1H)
13	141.0[a]	-	142.26	-
14	28.25	2.54-2.47 (m, 1H)<br>2.01 (ddd, *J* = 12.8, 12.8, 10.5 Hz, 1H)	29.67	2.59 (dd, *J* = 13.3, 3.8 Hz, 1H)<br>2.19-2.07 (m, 1H)
15	23.68	3.30-3.25 (m, 1H)	25.19	3.46-3.40 (m, 1H)
16	109.01	-	109.98	-
17	145.05	7.05 (d, *J* = 2.7 Hz, 1H)	147.74	7.18 (d, *J* = 2.6 Hz, 1H)
18	120.49	5.48 (dd, *J* = 17.1, 1.7 Hz, 1H)<br>5.35 (dd, *J* = 10.3, 2.0 Hz, 1H)	121.46	5.52 (dd, *J* = 17.0, 1.8 Hz, 1H)<br>5.40 (dd, *J* = 10.3, 2.0 Hz, 1H)
19	132.54	5.81 (dt, *J* = 17.2, 9.8 Hz, 1H)	133.50	5.87 (dt, *J* = 17.2, 10.1 Hz, 1H)
20	43.62	2.69-2.62 (m, 1H)	45.99	2.73-2.68 (m, 1H)
21	94.82	5.39 (d, *J* = 1.5 Hz, 1H)	97.44	5.51 (d, *J* = 1.7 Hz, 1H)
22	163.97	-	167.57	-
1‘	97.79	4.55 (d, *J* = 7.9 Hz, 1H)	99.80	4.73 (d, *J* = 7.9 Hz, 1H)
2‘	73.17	3.03-2.96 (m, 1H)	74.79	3.20 (dd, *J* = 9.2, 7.9 Hz, 1H)
3‘	76.48	3.21-3.14 (m, 1H)	77.94	3.42-3.37 (m, 1H)
4‘	70.09	3.07-3.00 (m, 1H)	71.53	3.34-3.26 (m, 1H)
5‘	77.31	3.21-3.14 (m, 1H)	78.34	3.38-3.29 (m, 1H)
6‘	61.08	3.70 (d, *J* = 10.5 Hz, 1H)<br>3.48-3.37 (m, 1H)	62.67	3.91 (dd, *J* = 12.0, 2.2 Hz, 1H)<br>3.68 (dd, *J* = 12.0, 5.8 Hz, 1H)

[a] These signals were not observed in the ^{13}C{^{1}H} NMR spectrum most likely due to signal broadening caused by tautomerization. However, they could be assigned based on the ^{1}H-^{13}C HSQC and ^{1}H-^{13}C HMBC NMR spectra.

[b] Overlap with residual solvent signal.

Table S2. ^{1}H and ^{13}C NMR assignments of (3*R*)-pumiloside (**15**) (DMSO-d_6, 600 MHz, 298 K).

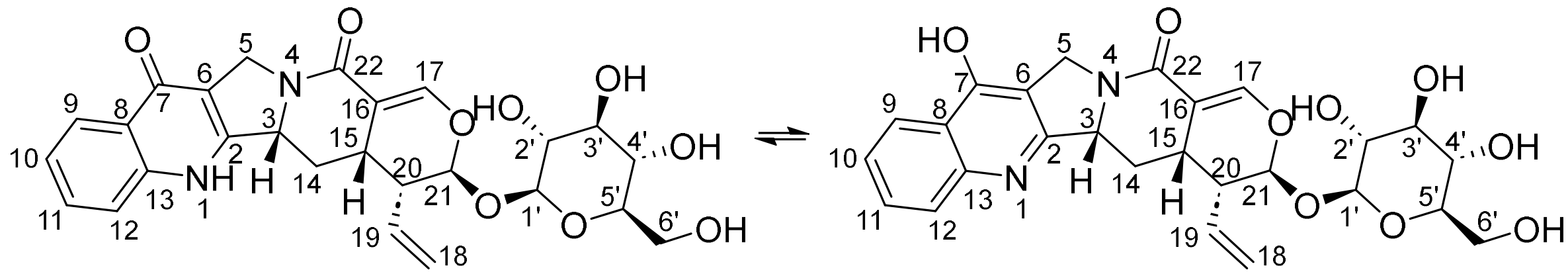

(3*R*)-Pumiloside (**15**)

Atom	[13]C ppm	[1]H ppm (m, Hz)	[13]C ppm	[1]H ppm (m, Hz)
	DMSO-d_6		MeOD-d_4	
1	N*H*	8.48 (s, 1H)	N*H*	-
2	-[a]	-	152.44	-
3	60.68	5.13-5.07 (m, 1H)	62.42	5.23-5.16 (m, 1H)
4	-[a]	-	-	-
5	48.21	4.74 (dd, *J* = 14.5, 2.1 Hz, 1H)<br>4.25 (dd, *J* = 14.5, 1.7 Hz, 1H)	49.49[b]	5.00 (dd, *J* = 14.9, 2.0 Hz, 1H)<br>4.49 (dd, *J* = 14.9, 1.5 Hz, 1H)
6	-[a]	-	114.92	-
7	-[a]	-	175.97	-
8	125.52	-	126.63	-
9	124.67	8.12 (d, *J* = 8.0 Hz, 1H)	126.14	8.29 (dd, *J* = 8.3, 0.9 Hz, 1H)
10	-[a]	7.31 (dd, *J* = 6.2, 6.2 Hz, 1H)	125.32	7.43 (ddd, *J* = 8.2, 6.9, 1.2 Hz, 1H)
11	-[a]	7.67-7.56 (m, 1H)	133.40	7.72 (ddd, *J* = 8.4, 6.9, 1.5 Hz, 1H)
12	-[a]	7.60 (d, *J* = 7.8 Hz, 1H)	119.57	7.64 (dd, *J* = 8.4, 0.7 Hz, 1H)
13	-[a]	-	142.06	-
14	29.13	2.56-2.49 (m, 1H),<br>1.37 (ddd, *J* = 12.3, 12.3, 12.3 Hz, 1H)	30.48	2.54 (ddd, *J* = 12.3, 3.7, 3.7 Hz, 1H)<br>1.59 (ddd, *J* = 13.2, 12.0, 12.0 Hz, 1H)
15	27.65	3.20-3.14 (m, 1H)	29.36	3.39-3.29 (m, 1H)
16	107.37	-	108.29	-
17	146.36	7.34 (d, *J* = 2.5 Hz, 1H)	149.40	7.51 (d, *J* = 2.5 Hz, 1H)
18	120.06	5.32 (dd, *J* = 17.1, 2.2 Hz, 1H)<br>5.20 (dd, *J* = 10.2, 2.2 Hz, 1H)	120.77	5.33 (dd, *J* = 17.1, 1.9 Hz, 1H)<br>5.23 (dd, *J* = 10.3, 2.0 Hz, 1H)
19	132.70	5.47 (dt, *J* = 17.1, 9.9 Hz, 1H)	133.57	5.55 (dt, *J* = 17.1, 10.0 Hz, 1H)
20	42.54	2.74 (dd, *J* = 9.6, 5.7 Hz, 1H)	44.73	2.79 (ddd, *J* = 9.7, 5.4, 1.8 Hz, 1H)
21	94.99	5.43 (d, *J* = 1.8 Hz, 1H)	97.48	5.57 (d, *J* = 1.8 Hz, 1H)
22	161.71	-	165.31	-
1‘	97.96	4.54 (d, *J* = 7.9 Hz, 1H)	99.64	4.73 (d, *J* = 7.9 Hz, 1H)
2‘	73.16	3.04-2.98 (m, 1H)	74.85	3.22 (dd, *J* = 9.2, 7.9 Hz, 1H)
3‘	76.47	3.21-3.15 (m, 1H)	77.97	3.43-3.37 (m, 1H)
4‘	70.07	3.07-3.02 (m, 1H)	71.58	3.35-3.26 (m, 1H)
5‘	77.30	3.21-3.15 (m, 1H)	78.37	3.38-3.30 (m, 1H)
6‘	61.06	3.70 (dd, *J* = 11.8, 3.5 Hz, 1H)<br>3.47-3.41 (m, 1H)	62.67	3.92 (dd, *J* = 12.0, 2.1 Hz, 1H)<br>3.69 (dd, *J* = 11.9, 5.7 Hz, 1H)

[a] These signals were not observed in the ^{13}C{^{1}H} NMR spectrum most likely due to signal broadening caused by tautomerization.

[b] Overlap with residual solvent signal.

Table S3. ^{1}H and ^{13}C NMR assignments of vincosamide ketolactam (**13**) (DMSO-d_6, 600 MHz, 298 K, or MeOD-d_4, 600 MHz, 298 K).

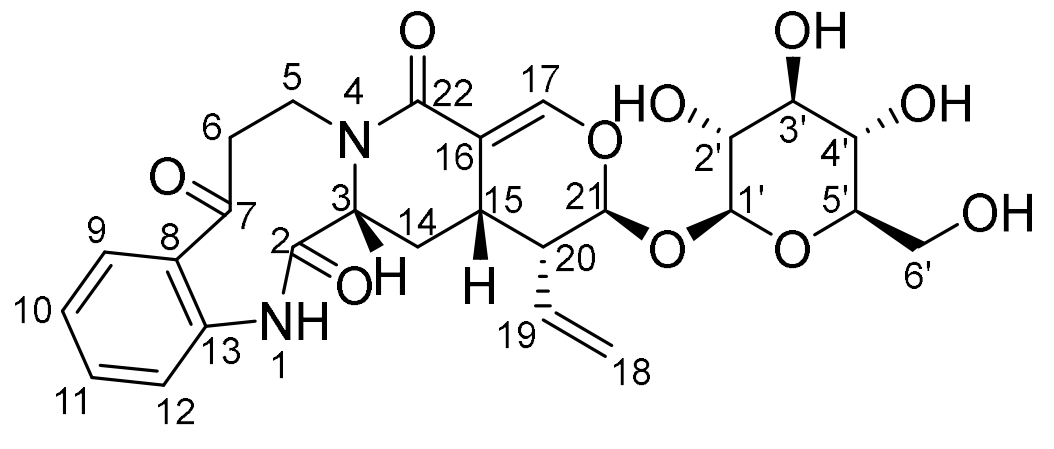

Vincosamide ketolactam (**13**)

Atom	^{13}C ppm	^{1}H ppm (m, Hz)	^{13}C ppm	^{1}H ppm (m, Hz)
	DMSO-d_6		MeOD-d_4	
1	N*H*	10.36 (s, 1H)	N*H*	-
2	171.38	-	173.98	-
3	64.73	4.04 (dd, *J* = 12.1, 4.1 Hz, 1H)	66.62	4.14 (dd, *J* = 12.2, 4.1 Hz, 1H)
4	-	-	-	-
5	42.37	4.59-4.52 (m, 1H)<br>2.77-2.72 (m, 1H)	44.34	4.69-4.64 (m, 1H)<br>2.97-2.92 (m, 1H)
6	44.26	2.81-2.73 (m, 2H)	45.78	2.89-2.84 (m, 2H)
**7**	203.65	-	205.90	-
8	139.05	-	140.61	-
9	128.15	7.43 (dd, *J* = 7.7, 1.9 Hz, 1H)	129.67	7.54 (dd, *J* = 7.7, 1.5 Hz, 1H)
10	127.17	7.40 (ddd, *J* = 7.4, 7.4, 1.1 Hz, 1H)	128.96[a]	7.44 (ddd, *J* = 7.6, 7.6, 1.2 Hz, 1H)
11	132.23	7.58 (ddd, *J* = 7.5, 7.5, 1.9 Hz, 1H)	133.70	7.61 (ddd, *J* = 7.6, 7.6, 1.6 Hz, 1H)
12	127.87	7.25 (dd, *J* = 7.8, 1.1 Hz, 1H)	128.98[a]	7.28 (dd, *J* = 7.9, 1.1 Hz, 1H)
13	136.69	-	137.33	-
14	26.00	2.04 (ddd, *J* = 12.6, 4.1, 4.1 Hz, 1H)<br>1.94 (ddd, *J* = 12.7, 12.7, 12.7 Hz, 1H)	28.20	2.14 (ddd, *J* = 12.9, 4.1, 4.1 Hz, 1H)<br>2.03 (ddd, *J* = 12.9, 12.9, 12.9 Hz, 1H)
15	25.86	2.99-2.93 (m, 1H)	27.40	3.16-3.10 (m, 1H)
16	107.94	-	109.10	-
17	146.42	7.31 (d, *J* = 2.4 Hz, 1H)	149.28	7.45 (d, *J* = 2.3 Hz, 1H)
18	120.14	5.38 (dd, *J* = 16.8, 1.9 Hz, 1H)<br>5.32 (dd, *J* = 10.2, 2.1 Hz, 1H)	120.82	5.39 (dd, *J* = 17.1, 1.8 Hz, 1H)<br>5.34 (dd, *J* = 10.2, 2.0 Hz, 1H)
19	133.20	5.66 (dt, *J* = 17.2, 10.0 Hz, 1H)	134.05	5.72 (dt, *J* = 17.2, 10.1 Hz, 1H)
20	42.26	2.67 (ddd, *J* = 9.8, 5.7, 1.8 Hz, 1H)	44.48	2.74 (ddd, *J* = 9.9, 5.8, 1.9 Hz, 1H)
21	95.17	5.37 (d, *J* = 1.9 Hz, 1H)	97.30	5.52 (d, *J* = 1.9 Hz, 1H)
22	163.13	-	166.50	-
1‘	98.08	4.48 (d, *J* = 7.9 Hz, 1H)	99.58	4.67 (d, *J* = 7.9 Hz, 1H)
2‘	73.08	3.00-2.95 (m, 1H)	74.73	3.18 (dd, *J* = 9.2, 7.9 Hz, 1H)
3‘	76.53	3.18-3.12 (m, 1H)	77.89	3.37 (dd, *J* = 9.0, 9.0 Hz, 1H)
4‘	70.03	3.06-3.01 (m, 1H)	71.56	3.30-3.26 (m, 1H)
5‘	77.31	3.18-3.12 (m, 1H)	78.36	3.35-3.28 (m, 1H)
6‘	61.02	3.69 (d, *J* = 11.8 Hz, 1H)<br>3.46-3.41 (m, 1H)	62.70	3.90 (dd, *J* = 12.0, 2.2 Hz, 1H)<br>3.67 (dd, *J* = 12.0, 5.9 Hz, 1H)

[a] Signals are interchangeable.

Table S4. ^{1}H and ^{13}C NMR assignment comparison between vincosamide ketolactam (**13**) and reported data for turpiniside[21] (MeOD-d_4).

Atom	^{13}C ppm	^{1}H ppm (m, Hz)	^{13}C ppm	^{1}H ppm (m, Hz)
	**Vincosamide ketolactam**		**Turpiniside reported data[21]**	
1	N*H*	-	N*H*	-
2	173.98	-	173.9	-
3	66.62	4.14 (dd, *J* = 12.2, 4.1 Hz, 1H)	66.5	4.12 (dd, *J* = 12.7, 4.1 Hz, 1H)
4	-	-	-	-
5	44.34	4.69-4.64 (m, 1H)<br>2.97-2.92 (m, 1H)	44.2	4.61 (m, 1H)<br>2.91 (m, 1H)
6	45.78	2.89-2.84 (m, 2H)	45.7	2.85 (m, 2H)
**7**	205.90	-	206.0	-
8	140.61	-	140.1	-
9	129.67	7.54 (dd, *J* = 7.7, 1.5 Hz, 1H)	129.6	7.52 (d, *J* = 7.6 Hz, 1H)
10	128.96[a]	7.44 (ddd, *J* = 7.6, 7.6, 1.2 Hz, 1H)	128.9	7.42 (t, *J* = 7.6 Hz, 1H)
11	133.70	7.61 (ddd, *J* = 7.6, 7.6, 1.6 Hz, 1H)	133.7	7.59 (t, *J* = 7.6 Hz, 1H)
12	128.98[a]	7.28 (dd, *J* = 7.9, 1.1 Hz, 1H)	128.9	7.26 (d, *J* = 7.6 Hz, 1H)
13	137.33	-	137.4	-
14	28.20	2.14 (ddd, *J* = 12.9, 4.1, 4.1 Hz, 1H)<br>2.03 (ddd, *J* = 12.9, 12.9, 12.9 Hz, 1H)	28.1	2.12 (dt, *J* = 12.7, 4.1 Hz, 1H)<br>2.00 (q, *J* = 12.7 Hz, 1H)
15	27.40	3.16-3.10 (m, 1H)	27.3 [a]	3.10 (m, 1H)
16	109.10	-	109.0 [a]	-
17	149.28	7.45 (d, *J* = 2.3 Hz, 1H)	149.3 [a]	7.44 (brs, 1H)
18	120.82	5.39 (dd, *J* = 17.1, 1.8 Hz, 1H)<br>5.34 (dd, *J* = 10.2, 2.0 Hz, 1H)	120.9 [a]	5.38 (dd, *J* = 17.3, 1.8 Hz, 1H)<br>5.32 (dd, *J* = 10.0, 1.8 Hz, 1H)
19	134.05	5.72 (dt, *J* = 17.2, 10.1 Hz, 1H)	133.9 [a]	5.69 (dt, *J* = 17.3, 10.0 Hz, 1H)
20	44.48	2.74 (ddd, *J* = 9.9, 5.8, 1.9 Hz, 1H)	44.2 [a]	2.74 (ddd, *J* = 10.0, 5.8, 1.8 Hz, 1H)
21	97.30	5.52 (d, *J* = 1.9 Hz, 1H)	97.3	5.50 (brs, 1H)
22	166.50	-	166.4[a]	-
1‘	99.58	4.67 (d, *J* = 7.9 Hz, 1H)	99.5	4.67 (d, *J* = 7.9 Hz, 1H)
2‘	74.73	3.18 (dd, *J* = 9.2, 7.9 Hz, 1H)	74.6	3.20 (t, *J* = 8.5 Hz, 1H)
3‘	77.89	3.37 (dd, *J* = 9.0, 9.0 Hz, 1H)	77.8	3.39 (m, 1H)
4‘	71.56	3.30-3.26 (m, 1H)	71.5	3.29 (m, 1H)
5‘	78.36	3.35-3.28 (m, 1H)	78.2	3.36 (m, 1H)
6‘	62.70	3.90 (dd, *J* = 12.0, 2.2 Hz, 1H)<br>3.67 (dd, *J* = 12.0, 5.9 Hz, 1H)	62.6	3.89 (dd, *J* = 11.8, 2.0 Hz, 1H)<br>3.67 (dd, *J* = 11.8, 5.6 Hz, 1H)

[a] For the reported data of turpiniside,[21] a different carbon numbering system was used. The data shown here was adjusted to match our carbon numbering scheme to ensure consistency for the comparison.

Table S5. Comparison of MS/MS fragments of the C-3 epimers strictosamide ketolactam (**7**) and vincosamide ketolactam (**13**) under multiple collision energies with literature.[7]

Measured *m*/*z* values by us were rounded to the nearest integer from original high-resolution data for better comparability with literature data.[7] Original high-resolution data is provided in Table S6. Spectra are shown in Figure S10. Values in parentheses represent relative intensities. Signals below 5% are omitted. CE, collision energy.

**Strictosamide ketolactam (7)**				**Vincosamide ketolactam (13)**				**Ref. [7] Isomer 1**	**Ref. [7] Isomer 2**
m/*z* (rel. int.)				*m*/*z* (rel. int.)				*m*/*z* (rel. int.)	
CE 10	CE 20	CE 40	CE 60	CE 10	CE 20	CE 40	CE 60	CE 10, 20, 40 and 60	
531 (7)				531 (100)	531 (8)			531 (56)	531 (62)
369 (100)	369 (100)			369 (44)	369 (86)			369 (56)	369 (58)
	351 (6)				351 (21)			351 (3)	351 (9)
	341 (8)							341 (4)	341 (3)
					323 (6)				
	299 (16)			299 (15)	299 (100)	299 (7)		299 (9)	299 (18)
	271 (20)				271 (6)			271 (13)	271 (13)
								253 (2)	253 (3)
		194 (8)				194 (7)		194 (5)	194 (10)
		176 (21)	176 (6)			176 (18)	176 (5)	176 (17)	176 (16)
		158 (6)	158 (6)			158 (12)	158 (12)	158 (4)	158 (5)
	148 (9)	148 (100)	148 (71)		148 (12)	148 (84)	148 (55)	148 (75)	148 (88)
		130 (6)	130 (53)			130 (7)	130 (49)	130 (58)	130 (54)
	124 (11)	124 (97)	124 (100)		124 (17)	124 (100)	124 (100)	124 (100)	124 (100)
			106 (10)				106 (10)	106 (8)	106 (13)
				59 (5)					

Table S6. High-resolution MS/MS data of the C-3 epimers strictosamide ketolactam (**7**) and vincosamide ketolactam (**13**) under multiple collision energies.

Signals below 1% are omitted. CE, collision energy.

**Strictosamide ketolactam (7)**		**Vincosamide ketolactam (13)**	
CE	*m/z* (%)	CE	*m/z* (%)
10	369.1439 (100.00%), 531.1965 (7.07%)	10	531.1959 (100.00%), 369.1436 (44.17%), 299.1020 (14.59%), 59.0224 (5.38%), 351.1333 (2.69%), 59.0190 (1.57%)
20	369.1437 (100.00%), 271.1073 (20.44%), 299.1021 (15.64%), 124.0393 (10.57%), 148.0755 (8.94%), 341.1491 (8.24%), 351.1334 (5.79%), 176.0704 (4.24%), 194.0812 (1.53%)	20	299.1021 (100.00%), 369.1438 (86.25%), 351.1334 (21.37%), 124.0394 (17.46%), 148.0755 (12.12%), 531.1967 (8.20%), 271.1075 (6.33%), 323.1386 (6.32%), 176.0704 (3.49%), 194.0811 (1.76%), 341.1486 (1.42%)
25	369.1438 (100.00%), 124.0393 (63.35%), 148.0755 (52.45%), 271.1072 (48.47%), 299.1021 (36.06%), 176.0704 (21.99%), 351.1333 (14.89%), 341.1490 (12.00%), 194.0809 (9.68%), 158.0600 (2.28%), 323.1390 (2.03%), 253.0966 (2.00%), 281.0919 (1.98%), 333.1232 (1.76%), 325.1183 (1.20%), 178.0502 (1.05%)	25	299.1020 (100.00%), 124.0393 (56.64%), 369.1438 (53.45%), 148.0755 (41.13%), 351.1333 (22.43%), 176.0704 (11.85%), 271.1074 (9.08%), 323.1385 (8.61%), 194.0810 (5.99%), 158.0598 (2.88%), 281.0919 (2.27%), 333.1227 (2.21%), 85.0288 (1.81%), 341.1491 (1.26%), 200.1069 (1.24%), 145.0492 (1.08%)
30	124.0394 (100.00%), 148.0755 (95.17%), 369.1439 (33.06%), 176.0704 (32.72%), 271.1073 (29.80%), 299.1021 (25.82%), 194.0810 (15.15%), 351.1333 (11.17%), 158.0599 (4.60%), 341.1489 (3.92%), 253.0964 (3.56%), 281.0918 (3.47%), 178.0495 (1.82%), 333.1235 (1.77%), 323.1389 (1.61%), 130.0651 (1.31%), 85.0288 (1.29%), 243.0761 (1.24%), 146.0598 (1.04%), 325.1170 (1.03%)	30	124.0393 (100.00%), 148.0754 (77.55%), 299.1020 (61.29%), 369.1438 (21.30%), 176.0704 (21.08%), 351.1332 (14.64%), 194.0810 (9.63%), 158.0598 (7.40%), 323.1385 (7.30%), 271.1073 (7.24%), 281.0916 (3.86%), 85.0288 (3.04%), 333.1227 (2.25%), 200.1067 (1.65%), 226.0495 (1.41%), 243.0762 (1.34%), 248.0911 (1.29%), 130.0649 (1.26%), 127.0387 (1.10%), 230.0805 (1.09%)
40	148.0755 (100.00%), 124.0393 (97.30%), 176.0705 (20.76%), 194.0811 (8.24%), 130.0651 (6.40%), 158.0599 (5.67%), 299.1021 (4.38%), 281.0917 (4.04%), 271.1075 (2.82%), 253.0966 (2.28%), 106.0653 (2.11%), 146.0599 (2.06%), 160.0754 (1.83%), 178.0501 (1.73%), 351.1335 (1.70%), 85.0288 (1.55%), 96.0448 (1.48%), 369.1436 (1.37%), 226.0497 (1.34%), 126.0549 (1.05%)	40	124.0393 (100.00%), 148.0755 (83.57%), 176.0704 (18.01%), 158.0598 (11.74%), 299.1021 (7.27%), 130.0650 (6.58%), 194.0810 (6.56%), 281.0917 (3.52%), 226.0496 (3.17%), 85.0288 (2.92%), 106.0653 (2.38%), 126.0550 (2.01%), 351.1329 (1.89%), 323.1385 (1.60%), 133.0647 (1.18%), 206.0808 (1.06%), 200.1070 (1.05%), 369.1440 (1.01%)
60	124.0394 (100.00%), 148.0755 (71.13%), 130.0652 (53.18%), 106.0654 (10.42%), 158.0599 (6.29%), 176.0705 (5.89%), 96.0448 (3.98%), 146.0600 (3.14%), 160.0756 (2.80%), 55.0186 (2.64%), 120.0809 (2.51%), 140.0341 (2.44%), 85.0288 (2.13%), 120.0444 (2.01%), 126.0551 (1.88%), 122.0238 (1.62%), 198.0550 (1.45%), 108.0446 (1.21%), 69.0341 (1.18%), 132.0808 (1.11%)	60	124.0393 (100.00%), 148.0755 (55.28%), 130.0651 (49.45%), 158.0599 (12.41%), 106.0654 (9.89%), 176.0704 (4.88%), 126.0550 (3.28%), 85.0288 (3.14%), 122.0238 (2.96%), 120.0809 (2.71%), 55.0186 (2.48%), 140.0340 (2.27%), 120.0446 (2.18%), 146.0599 (2.16%), 105.0701 (1.87%), 69.0341 (1.83%), 96.0447 (1.82%), 198.0546 (1.79%), 162.0548 (1.47%), 97.0287 (1.37%), 226.0500 (1.23%), 133.0646 (1.19%), 108.0446 (1.18%)

Table S7. ^{1}H and ^{13}C NMR assignments of strictosamide (**5**) (DMSO-d_6, 600 MHz, 298 K).

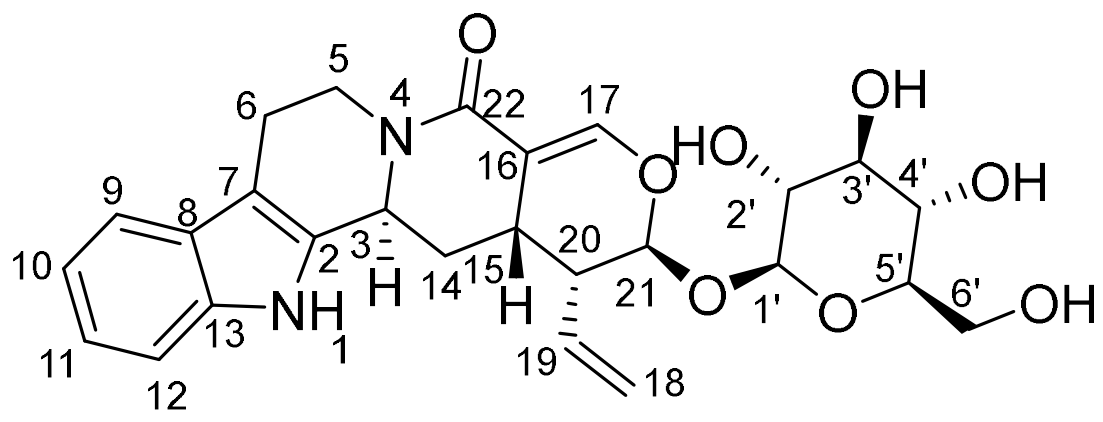

Strictosamide (**5**)

Atom	^{13}C ppm	^{1}H ppm (m, Hz)
1	N*H*	10.98 (s, 1H)
2	134.56	-
3	52.65	5.03 (d, *J* = 4.0 Hz, 1H)
4	-	-
5	42.41	4.79 (dd, *J* = 12.6, 6.0 Hz, 1H)<br>3.02-2.96 (m, 1H)
6	20.64	2.84-2.76 (m, 1H)<br>2.64-2.58 (m, 1H)
7	108.43	-
8	127.01	-
9	117.66	7.37 (d, *J* = 7.8 Hz, 1H)
10	118.73	7.00-6.96 (m, 1H)
11	121.01	7.09-7.05 (m, 1H)
12	111.34	7.34 (d, *J* = 8.1 Hz, 1H)
13	135.67	-
14	25.65	2.51-2.46 (m, 1H)<br>1.89 (ddd, *J* = 13.8, 13.8, 5.9 Hz, 1H)
15	23.45	2.64-2.59 (m, 1H)
16	107.53	-
17	146.65	7.24 (d, *J* = 2.2 Hz, 1H)
18	120.00	5.36 (dd, *J* = 17.1, 1.9 Hz, 1H)<br>5.32 (dd, *J* = 10.2, 1.9 Hz, 1H)
19	133.34	5.63-5.55 (m, 1H)
20	42.87	2.61-2.56 (m, 1H)
21	95.75	5.31 (d, *J* = 1.5 Hz, 1H)
22	163.46	-
1‘	98.91	4.43 (d, *J* = 8.0 Hz, 1H)
2‘	72.67	2.84-2.78 (m, 1H)
3‘	76.74	3.06 (ddd, *J* = 8.9, 8.9, 4.0 Hz, 1H)
4‘	69.89	2.98-2.91 (m, 1H)
5‘	77.20	3.13-3.09 (m, 1H)
6‘	60.96	3.66 (dd, *J* = 11.6, 3.6 Hz, 1H)<br>3.44-3.37 (m, 1H)
2’-O*H*	-	4.85 (d, *J* = 5.4 Hz, 1H)
3’-O*H*	-	4.87 (d, *J* = 4.7 Hz, 1H)
4’-O*H*	-	4.89 (d, *J* = 5.3 Hz, 1H)
6’-O*H*	-	4.54 (t, *J* = 5.4 Hz, 1H)

Table S8. ^{1}H and ^{13}C NMR assignments of vincosamide (**12**) (DMSO-d_6, 400 MHz, 298 K).

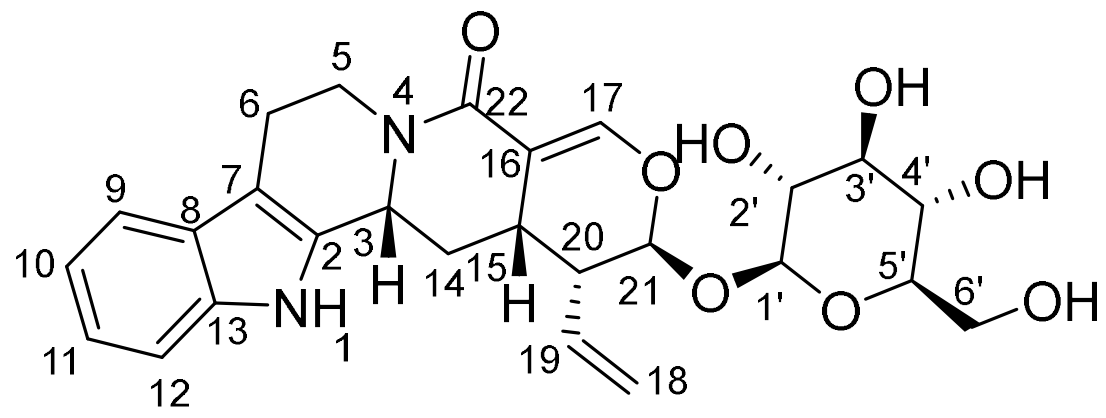

Vincosamide (**12**)

Atom	^{13}C ppm	^{1}H ppm (m, Hz)
1	N*H*	10.92 (s, 1H)
2	134.16	-
3	52.44	4.91 (d, *J* = 11.4 Hz, 1H)
4	-	-
5	38.60	4.98 (dd, *J* = 12.3, 4.6 Hz, 1H)<br>2.83 (td, *J* = 12.3, 3.8 Hz, 1H)
6	20.78	2.78-2.69 (m, 1H)<br>2.65-2.55 (m, 1H)
7	107.24	-
8	126.25	-
9	117.80	7.41 (d, *J* = 7.8 Hz, 1H)
10	118.61	7.00-6.94 (m, 1H)
11	121.06	7.09-7.03 (m, 1H)
12	111.12	7.31 (d, *J* = 7.4 Hz, 1H)
13	136.22	-
14	31.02	2.54-2.46 (m, 1H)<br>1.31-1.22 (m, 1H)
15	25.73	3.17-3.09 (m, 1H)
16	107.79	-
17	146.54	7.32 (s, 1H)
18	119.97	5.29 (dd, *J* = 17.2, 2.2 Hz, 1H)<br>5.17 (dd, *J* = 10.2, 2.2 Hz, 1H)
19	132.98	5.54-5.41 (m, 1H)
20	42.42	2.70-2.62 (m, 1H)
21	94.90	5.40 (d, *J* = 1.7 Hz, 1H)
22	162.45	-
1‘	97.92	4.52 (d, *J* = 7.9 Hz, 1H)
2‘	73.20	3.05-2.97 (m, 1H)
3‘	76.49	3.12-3.22 (m, 1H)
4‘	70.08	3.09-3.01 (m, 1H)
5‘	77.26	3.21-3.13 (m, 1H)
6‘	61.06	3.70 (d, *J* = 11.7 Hz, 1H)<br>3.45 (dd, *J* = 11.9, 6.3 Hz, 1H)
